# Chan–Evans–Lam *N*1-(het)arylation and *N*1-alkеnylation of 4-fluoroalkylpyrimidin-2(1*H*)-ones

**DOI:** 10.3762/bjoc.16.191

**Published:** 2020-09-17

**Authors:** Viktor M Tkachuk, Oleh O Lukianov, Mykhailo V Vovk, Isabelle Gillaizeau, Volodymyr A Sukach

**Affiliations:** 1 Institute of Organic Chemistry, National Academy of Sciences of Ukraine; 2 Institute of Organic and Analytical Chemistry, ICOA UMR 7311 CNRS, Université d’Orléans, rue de Chartres, 45100 Orléans, France; 3 Enamine LTD, 78 Chervonotkats‘ka str., Kyiv 02094, Ukraine

**Keywords:** C–N cross-coupling, Chan–Evans–Lam reaction, pyrimidin-2(1*Н*)-ones, fluoroalkyl group, boronic acids

## Abstract

The Chan–Evans–Lam reaction of 1-unsubstituted 4-fluoroalkylpyrimidin-2(1*Н*)-ones with arylboronic acids is reported as a facile synthetic route to hitherto unavailable *N*1-(het)aryl and *N*1-alkenyl derivatives of the corresponding pyrimidines. An efficient C–N bond-forming process is also observed by using boronic acid pinacol esters as coupling partners in the presence of Cu(II) acetate and boric acid. The 4-fluoroalkyl group on the pyrimidine ring significantly assists in the formation of the target *N*1-substituted products, in contrast to the 4-methyl and 4-unsubstituted substrates which do not undergo *N*1-arylation under similar reaction conditions.

## Introduction

The catalytic formation of C–N bonds in the presence of transition metal salts is an essential transformation that permits the preparation *N*-(het)aryl-substituted amines and their derivatives including various nitrogen-containing heterocycles [1–5], an important class of compounds throughout chemical research. The copper-catalyzed arylation of the nucleophilic nitrogen atom, known as the Ullmann [6–7] reaction, and its modification by Chan and Lam [8] are favored due to the several advantages they offer versus the Pd counterpart (i.e., the Buchwald–Hartwig reaction) such as the lower cost and lower toxicity of the metal as well as their tolerance of aerobic conditions [2,9]. The efficiency of the Ullmann arylation has recently been greatly improved through extensive mechanistic investigation, ligand/precatalyst design and optimization studies [10–13]. As a result, the reaction has become feasible under milder conditions, whereas originally it required very high temperatures, and some limitations including mostly low yields and intolerance of sensitive functional groups have been partially overcome [14]. A search for new efficient reagents for copper-catalyzed *N*-arylation has led to the recognition of arylboronic acids as uniquely advantageous means to perform C–N cross-coupling reactions [15–17]. The corresponding Ullmann-type reaction currently known as Chan–Evans–Lam (CEL) coupling is characterized by the combination of two nucleophilic reactants which implies that oxidative processes with atmospheric oxygen play a significant role in the generation of active copper-organic intermediates from boron-organic precursors [18–20].

**Figure 1:**
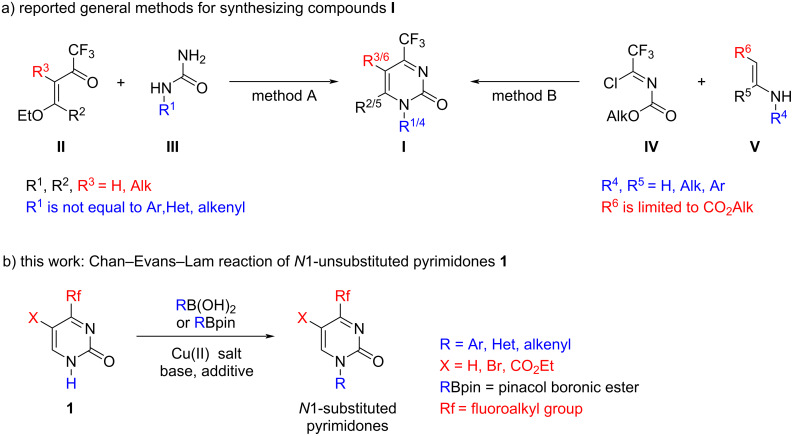
Summary of the previous and present studies.

Our interest in the development of *N*-arylation methods resonated with recent studies focused on the addition of various C-nucleophilic reagents to 4-trifluoromethylpyrimidin-2(1*H*)-ones I, heterocyclic analogues of activated ketimines (Figure 1), thus offering potential applications in the design of new heterocyclic chemotypes [21–25]. Compounds **I** are precursors of trifluoromethyl-substituted dihydropyrimidine derivatives which appear as original and potent scaffolds in medicinal chemistry, given the great importance of fluorinated groups in drug discovery [26–29]. On the strength of these results, herein we aim at extending the range of available compounds **I** by the introduction of (het)aryl and alkenyl substituents at the N1 position of the pyrimidine ring. Such derivatives have been hitherto unknown due to their synthetic inaccessibility by the conventional approach based on the formation of the pyrimidine moiety. For example, the attempted heterocyclization of ethoxyenones **II** (e.g., 4-ethoxy-1,1,1-trifluorobut-3-en-2-one) with *N*-arylureas, unlike the reaction with most *N*-alkylureas **III** (see Figure 1a, method A) [30], led to a complex mixture of products (unpublished data), which may be attributed to the decreased nucleophilicity of the nitrogen atom bound to the aryl group. On the other hand, a previously developed synthetic non-trivial method involving the cyclocondensation of *N-*(trifluorochloroethylidene) carbamates **IV** with β-amino crotonates **V** led only to 4-trifluoromethylpyrimidin-2(1*H*)-ones **I** bearing a 5-alkoxycarbonyl substituent (R^3/6^ = CO_2_Alk) due to the strong structural limitations of the nucleophilic enamine component (see Figure 1а, method В) [31]. The access to other *C5*-substituted and *C5*-unsubstituted pyrimidin-2(1*H*)-ones is beyond the synthetic scope of this approach.

At the same time, simple *N*1-unsubstituted pyrimidin-2(1*H*)-ones **1** (see Figure 1b) are readily available on a multigram scale from inexpensive reagents [32–34] and can serve as promising building blocks for further functionalization, including (het)aryl or alkenyl substitution at the N1 atom. However, direct arylation of **1** with aryl halides under Ullmann reaction conditions is a low-efficiency process giving only complex mixtures. It is likely that the harsh thermal conditions required for the coupling are not tolerated with the highly electrophilic ketimine moiety of 4-trifluoromethylpyrimidin-2(1*H*)-ones. The CEL reaction was successfully applied in the *N*-arylation of many heterocyclic systems [35–37] including non-fluorinated pyrimidin-2(1*H*)-ones [38–39] under mild conditions. There are a few thorough studies on this reaction with pyrimidine and purine nucleoside bases and their derivatives which are most closely related to fluorine-containing substrates **1** [40–42]. However, considering the high sensitivity and capricious nature of CEL reaction, we wish to report herein a general set that promotes the effective coupling of *N*1-unsubstituted 4-fluoroalkylpyrimidin-2(1*H*)-ones **1** with (het)aryl- and alkenylboronic acids as well as with their pinacol esters. As the main synthetic result of the study, we have obtained the first representatives of hitherto unavailable 4-fluoroalkylpyrimidin-2(1*H*)-ones bearing (het)aryl and alkenyl substituents, respectively, at the N1 position (see Figure 1b).

## Results and Discussion

Our investigation started with 4-trifluoromethylpyrimidin-2(1*H*)-one (**1а**) as a model substrate in the presence of phenylboronic acid (**2a**) to study the effect of solvent, base, and temperature on the course of the CEL arylation in the presence of copper(II) acetate monohydrate (Table 1). As observed for dichloromethane medium, the reaction proceeded very efficiently, with a product **3a** yield of 88%, and reached completion within 48 h upon the addition of 2 equiv of pyridine (as a base and ligand), and 1 equiv of copper acetate at room temperature (see Table 1, entry 1). The product yield decreased slightly to 80% with 1 equiv of pyridine (Table 1, entry 2) and dropped dramatically to below 5% under pyridine-free conditions (Table 1, entry 3). With decreased amounts of both pyridine and copper acetate (0.4 and 0.2 equiv, respectively), the yield was reduced insignificantly to 81% suggesting, however, the catalytic nature of the conversion (Table 1, entry 4). Boiling the reaction mixture for 10 h led to a markedly reduced yield of 45% (Table 1, entry 5). The replacement of pyridine by other organic bases/ligands (4-DMAP, 2,2’-bipy, Et_3_N, TMEDA, 8-hydroxyquinoline) resulted in poorer yields of the target product in all cases (Table 1, entries 6–10). The use of copper(II) fluoride (in contrast to triflate) instead of acetate had practically no effect on the reaction course under the same conditions (Table 1, entries 11 and 12). Therefore, further solvent screening was carried out using pyridine (2 equiv) and copper acetate (1 equiv) as the efficient and most available system. The conversion under study proved to be sensitive to the nature of the solvent. Average product yields of 38–69% were observed for the reaction run in methanol, dichloroethane, tetrahydrofuran, and ethyl acetate (Table 1, entries 13–15, 20 and 21). In dimethyl carbonate and DMSO solutions, the yields were reduced to 4 and 11%, respectively (Table 1, entries 22 and 23). In contrast, with acetonitrile used as a solvent, the yield of product **3a** reached 83%. When the reaction in acetonitrile was prolonged to 96 h, the yield improved to 92% (Table 1, entries 16 and 17). Unlike the experiment in dichloromethane at 40 °С (Table 1, entry 5), heating to 80 °С and reduction of the reaction time to 8 h did not reduce the yield, which remained essentially the same, about 90% (Table 1, entry 18). Heating the reaction mixture also allowed the copper salt and pyridine to be used in substantially lower (catalytic) amounts, though sacrificing the yield to some extent (79%, Table 1, entry 19). Structural determination of compound **3а** was performed by IR and ^1^Н, ^13^С, and ^19^F NMR spectroscopy as well as by LCMS and HRMS analysis.

**Table 1:** Effect of the solvent, base, and temperature on the CEL reaction of 4-trifluoromethylpyrimidin-2(1*H*)-one (**1а**) with phenylboronic acid (**2a**).

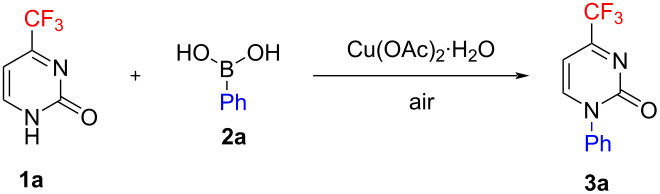						

Entry	Solvent^a^	Additive, equiv	Cu(OAc)_2_·H_2_O, equiv	*t*, °С	Time, h	Yield **3a**,^b^ %

1	CH_2_Cl_2_	Py, 2	1	rt	48	88
2	CH_2_Cl_2_	Py,1	1	rt	48	80
3	CH_2_Cl_2_	–	1	rt	48	<5
4	CH_2_Cl_2_	Py, 0.4	0.2	rt	48	81
5	CH_2_Cl_2_	Py, 2	1	40	10	45
6	CH_2_Cl_2_	4-DMAP, 2	0.2	rt	48	19
7	CH_2_Cl_2_	2,2’-bipy, 2	0.2	rt	48	15
8	CH_2_Cl_2_	Et_3_N, 2	1	rt	48	61
9	CH_2_Cl_2_	TMEDA, 2	1	rt	48	44
10	CH_2_Cl_2_	8-hydroxy-quinoline, 2	1	rt	48	18
11	CH_2_Cl_2_	Py, 2	CuF_2_ (1 equiv)	rt	48	86
12	CH_2_Cl_2_	Py, 2	Cu(OTf)_2_ (1 equiv)	rt	48	27
13	MeOH	Py, 2	1	rt	48	40
14	MeOH/H_2_O 9:1	Py, 2	1	rt	16	38
15	DCE	Py, 2	1	rt	48	69
16	MeCN	Py, 2	1	rt	48	83
**17**	**MeCN**	**Py, 2**	**1**	**rt**	**96**	**92**
**18**	**MeCN**	**Py, 2**	**1**	**80**	**8**	**90**
19	MeCN	Py, 0.4	0.2	80	8	79
20	THF	Py, 2	1	rt	48	38
21	EtOAc	Py, 2	1	rt	48	65
22	DMC	Py, 2	1	rt	48	4
23	DMSO	Py, 2	1	rt	48	11

^a^The reaction was conducted in an open flask (with an air condenser) and was vigorously stirred; ^b^yields monitored by ^1^H NMR analysis of the isolated crude samples.

**Scheme 1:**
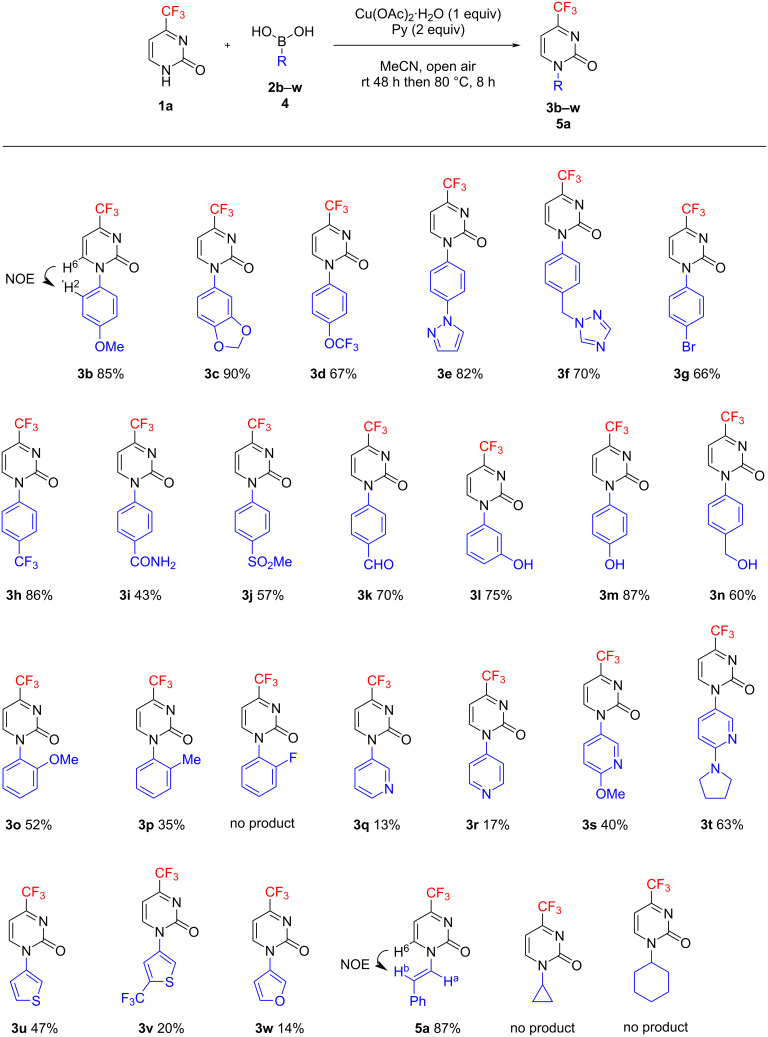
Chan–Evans–Lam reaction of 4-trifluoromethylpyrimidin-2(1*H*)-one **1а** with (het)aryl boronic acid **2b**–**w** and β-styrylboronic acid **4**.

Performing the reaction with substrate **1а** under the optimum conditions (see Table 1, entry 18), we examined a variety of (het)aryl- and alkenylboronic acids **2b**–**w** as coupling partners. As established in the first experiments, stirring the reaction mixture at room temperature for 24 h followed by heating at 80 °С in acetonitrile results in a considerably increased yield of the corresponding pyrimidones **3**. The thus optimized reaction conditions were applied to obtain a series of otherwise difficult to access and hitherto unknown *N*1-aryl-substituted 4-trifluoromethylpyrimidin-2(1*H*)-ones **3b**–**w** (Scheme 1). The product yields were found to depend on the electronic nature of the substituents on the phenyl ring. A number of commercially available phenylboronic acids with electron-donating or electron-withdrawing *para*-substituents on the aromatic ring were reacted with substrate **1a** to give pyrimidones **3** in satisfactory and high yields of 66–90%. Boronic acids containing *p*-CONH_2_, *p*-SO_2_Me and *p*-CH_2_OH substituents, respectively, provided the correspondingly *N*1-substituted pyrimidones **3i**,**j**,**n** in moderate to satisfactory yields of 43–60%.

It is notable that in our case, the Chan–Evans–Lam arylation is tolerant to many sensitive functional groups contained in boronic acids, thereby providing a synthetic entry to products **3** with the aldehyde (in compound **3k**), phenolic *meta*- and *para*-hydroxy (**3l**,**m**), and hydroxymethyl (**3n**) substituents. At the same time, the reaction with *para*-*N,N*-dimethylaminomethylphenylboronic acid failed to produce the desired arylated product due to the oxidation of the substituent to the aldehyde group and, probably, some other related side processes. As might be expected, the *ortho*-substituent on the phenyl ring of the boronic acid impeded *N*1-arylation by steric hindrance. However, the methoxy and methyl groups at the *ortho* position did not prevent the formation of the corresponding products **3o** and **3p**, which were isolated in moderate yields of 52 and 35%, respectively. In contrast, the fluorine *ortho*-substituent drastically inhibited the reaction, which is evidently attributed more to electronic than to steric effects.

**Scheme 2:**
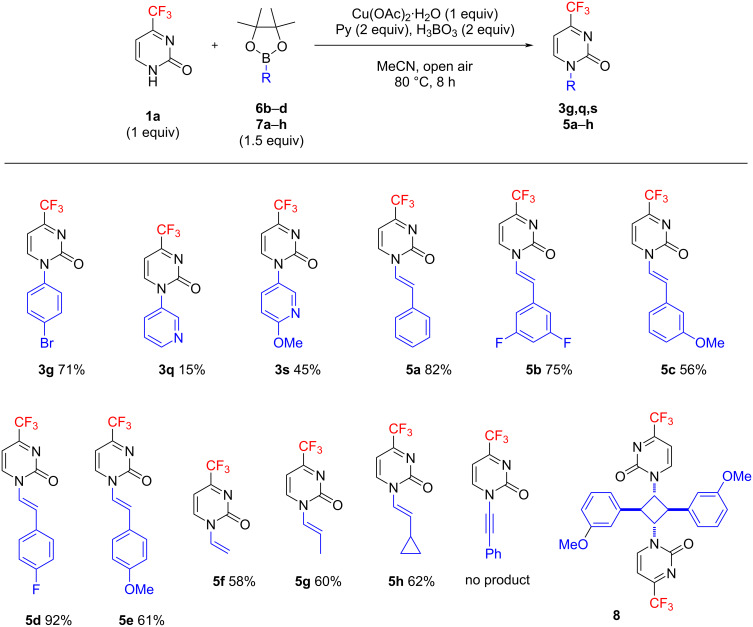
Chan–Evans–Lam reaction of 4-trifluoromethylpyrimidin-2(1*H*)-one (**1а**) with (het)aryl- and alkenylboronic acid pinacol esters **4b**–**d** and **7a**–**h**.

The formation of alternative *O*-arylated products was ruled out by conducting a NOE NMR experiment with compound **3b** as an example. Saturation of the signal from the pyrimidine H^6^ proton resulted in a significantly enhanced resonance of the *ortho* protons in the *para*-methoxyphenyl group (see Scheme 2 and Supporting Information File 1).

As the next step of the study, substrate **1a** was reacted with hetarylboronic acids containing various heterocyclic moieties to obtain the first representatives of hetaryl-substituted 4-trifluoromethylpyrimidin-2(1*H*)-ones **3**. The simplest 3- and 4-pyridylboronic acids provided the corresponding products **3q**,**r**, which were isolated and completely characterized in spite of tthe low yields (13–17%). Electron-donating substituents (a methoxy or 1-pyrrolidinyl group) at position 4 of 3-pyridylboronic acid enabled increased yields of the target products **3s** and **3t** thus opening a preparative pathway to a wide variety of 4-alkoxy-3-pyridyl and 4-dialkylamino-3-pyridyl derivatives. Hetarylboronic acids containing a more electron-deficient pyrimidyl instead of the pyridyl residue was completely unreactive to *N*1-hetarylation of substrate **1a** under the conditions used. Contrary to this case, the method developed allowed electron-rich heterocyclic nuclei including the 3-thienyl (but not isomeric 2-thienyl), 5-trifluoromethyl-3-thienyl, and 3-furyl residues, respectively, to be introduced at the N1 position of the pyrimidone ring, affording compounds **3u**–**w**.

Stimulated by the reported examples of the copper-catalyzed *N*-alkenylation of heterocycles [40,43–48], we extended the reaction scope to β-styrylboronic acid (**4**) as a reagent; the thus obtained *N*1-styryl-substituted 4-trifluoromethylpyrimidin-2(1*H*)-one **5a** obviously has considerable synthetic potential [49–50]. The stereochemistry of compound **5a** was confirmed by a NOE NMR experiment, which demonstrated significant spatial interaction between the H^6^ and H^b^ protons (see Scheme 1) and therefore suggested the preferred *s-trans* conformation of the β-styryl substituent in a CDCl_3_ solution (see Supporting Information File 1). It should be noted that the attempted *N*1-cycloalkylation did not proceed with cyclopropyl- and cyclohexylboronic acids.

Aiming at extending further the range of reagents, we subjected phenylboronic acid pinacol ester (**6а**) to a similar model conversion. Boronic acid pinacol esters are generally known to be much less reactive than the corresponding boronic acids in the Chan–Evans–Lam reaction having, on the other hand, the important advantage of being more available and stable [20,51–52]. A recently reported combined synthetic and spectroscopic study optimized the conditions for the C–N-coupling of NH compounds of various kinds (amines and NH-heterocycles) with boronic acid pinacol esters [20]. The use of acetonitrile as a solvent, with the addition of boric acid as a promoter, was shown to be most efficient. As found by us, starting from pyrimidone **1а**, when reacted with pinacol boronate **6а** (1.5 equiv), provided only a minor amount of product **3а** (about 9%) under the optimum conditions for the reactions with boronic acids (Table 2, entry 1). However, the addition of 2 equiv of boric acid drastically increased the arylation efficiency, with the yield of pyrimidone **3а** reaching 86% (Table 2, entry 2). We assume that the boric acid added triggers the transesterification of phenylboronic acid pinacol ester **6а** thereby leading to the in situ generation of reactive phenylboronic acid (**2а**). The reaction attempted at the lower (room) temperature and with lower amounts of copper acetate and pyridine practically failed to occur (Table 2, entries 3–5).

**Table 2:** Effect of boric acid and pyridine additives on the Chan–Evans–Lam reaction of 4-trifluoromethylpyrimidin-2(1*H*)-one (**1а**) with phenylboronic acid pinacol ester (**6а**).

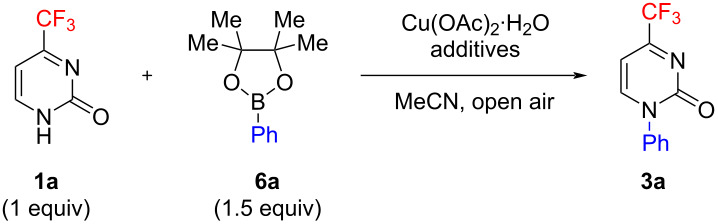					

Entry^b^	Additive, equiv	Cu(OAc)_2_·H_2_O equiv	*t*, °C	Time, h	Yield **3a**, %

1	Py, 2	1	80	8	9
2	Py, 2 H_3_BO_3_, 2	1	80	8	86
3	Py, 2 H_3_BO_3_, 2	1	20	48	7
4	Py, 0.4 H_3_BO_3_, 2	0.2	80	8	9
5	H_3_BO_3_, 2	1	80	8	<5

^a^The reaction was conducted in an open flask (with an air condenser) and was vigorously stirred; ^b^yields monitored by ^1^H NMR analysis of the isolated crude samples.

In view of the availability of boronic acid pinacol esters, and particularly of synthetically attractive pinacol alkenylboronates [53–54], we studied the reaction of **1а** with a series of such reagents **6b**–**d** and **7a**–**h** (Scheme 2). The yields of (het)aryl-substituted products **3g**,**q**,**s** obtained from **6b**–**d** were found to be much the same as in the analogous reactions of substrate **1а** with the corresponding boronic acids **2g**,**q**,**s**. Pinacol alkenyl boronates **7a**–**h** smoothly furnished the corresponding *N*1-alkenyl-substituted pyrimidones **5a**–**h**. Product **5f** obtained from the simplest vinylboronic acid pinacol ester (**7f**) appears to be of special value [55–56]. However, we did not succeed in reacting substrate **1а** with pinacol 2-phenylethynyl boronate. The *meta*-methoxystyryl derivative **5c** was found to easily undergo photodimerization in solid state upon sunlight exposure, leading to the formation of compound **8** with a central cyclobutane ring (see Supporting Information File 1). As proved previously for analogous enamides [57], the facile [2 + 2] photocycloaddition process likely occurred due to favorable orientation of the interacting molecules and close contacts between alkene carbon atoms in the crystal structure. Consequently, the synthesis of product **5c** was performed in darkness. *N*1-Styryl-substituted pyrimidin-2(1*H*)-ones **5a**–**e** exhibit fluorescence properties with emission of λ_max_ = 490–532 nm in CH_2_Cl_2_ solution.

**Scheme 3:**
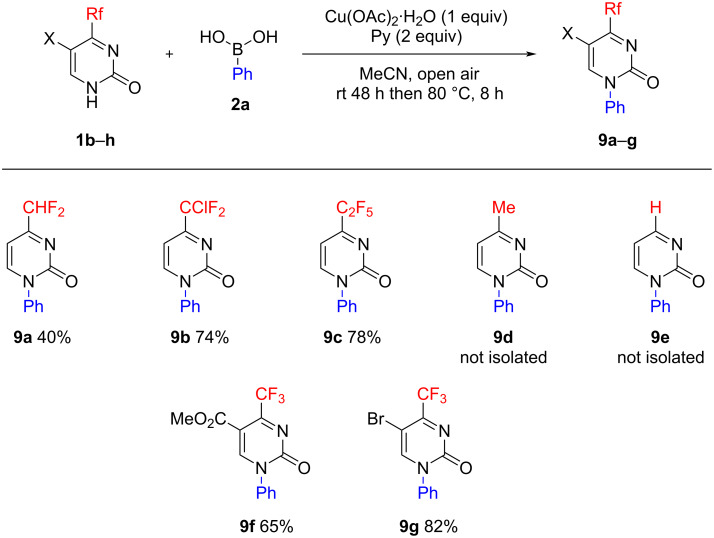
Chan–Evans–Lam reaction of pyrimidin-2(1*H*)-ones **1b**–**h** with phenylboronic acid (**2a**).

In order to analyze the effect exerted on the Chan–Evans–Lam arylation by 4-, 5-, and 6-substituents on the pyrimidine ring and also aiming at the further functionalization of pyrimidin-2(1*H*)-one, we studied various substrates **1b**–**h** in the reaction with phenylboronic acid (**2a**, Scheme 3). It was found that 4-difluoromethylpyrimidin-2(1*H*)-one (**1b**) produced the corresponding *N*1-phenyl derivative **9a** in a moderate yield of 40%, whereas its 4-difluorochloromethyl and 4-pentafluoroethyl-substituted analogues **1c,d** afforded products **9b,c** in high yields of 74 and 78%, respectively (see Supporting Information File 1). Unexpectedly, 4-methyl-substituted and 4-unsubstituted pyrimidin-2(1*H*)-ones **1e,f**, when reacted under similar conditions, gave complex mixtures containing no more than 5% of target products **9d**,**e**. Thus, the presence of the electron-withdrawing 4-fluoroalkyl group in pyrimidones **1** is a significant structural factor playing a major role in the overall success of the Chan–Evans–Lam reaction, most likely due to related increase in NH-acidity of the heterocyclic system. The ester group and the bromine atom at position 5 of the 4-trifluoromethylpyrimidin-2(1*H*)-ones **1g**,**h** did not disturb the course of the reaction and the products **9f**,**g** bearing the corresponding functionalities are applicable as building blocks in further syntheses. The 6-methyl substituent had a negative effect on the *N*-arylation process. As evidenced by LCMS analysis, the reaction with 6-methyl-4-(trifluoromethyl)pyrimidin-2(1*H*)-one provided a mixture of three cross-coupling products (not separated), with a total yield of no more than 18%, which suggests a poor regioselectivity.

## Conclusion

We have optimized the reaction conditions for the efficient and facile Chan–Evans–Lam *N*1-(het)arylation and *N*1-alkеnylation of 4-fluoroalkylpyrimidin-2(1*H*)-ones. It has been shown that with the addition of boric acid, high yields of the target products are also obtained when the reaction is carried out with boronic acid pinacol esters as an organoboron component, instead of the conventionally used boronic acids. Among the newly-synthesized compounds are the first representatives of *N*1-(het)aryl-4-fluoroalkylpyrimidin-2(1*H*)-ones as well as their synthetically promising *N*1-alkеnyl-substituted analogues. The success of the reaction has been found to depend on the presence of the 4-fluoroalkyl group in the starting pyrimidone.

## Supporting Information

File 1 Experimental procedures, characterization data, copies of the ^1^H and ^13^C NMR spectra.

## References

[1] Bariwal J, Van der Eycken E (2013). Chem Soc Rev.

[2] Ruiz-Castillo P, Buchwald S L (2016). Chem Rev.

[3] Monnier F, Taillefer M (2008). Angew Chem, Int Ed.

[4] Senra J D, Aguiar L C S, Simas A B C (2011). Curr Org Synth.

[5] Ricci A (2000). Modern Amination Methods.

[6] Monnier F, Taillefer M (2009). Angew Chem, Int Ed.

[7] Sambiagio C, Marsden S P, Blacker A J, McGowan P C (2014). Chem Soc Rev.

[8] Qiao J X, Lam P Y S, Hall D G (2011). Recent Advances in Chan–Lam Coupling Reaction: Copper‐Promoted C–Heteroatom Bond Cross‐Coupling Reactions with Boronic Acids and Derivatives. Boronic Acids: Preparation and Applications in Organic Synthesis, Medicine and Materials.

[9] Hartwig J F (1998). Acc Chem Res.

[10] Altman R A, Buchwald S L (2007). Nat Protoc.

[11] Jia X, Peng P (2019). Asian J Org Chem.

[12] Lo Q A, Sale D, Braddock D C, Davies R P (2018). ACS Catal.

[13] Surry D S, Buchwald S L (2010). Chem Sci.

[14] Quivelli A F, Vitale P, Perna F M, Capriati V (2019). Front Chem (Lausanne, Switz).

[15] Chan D M T, Monaco K L, Wang R-P, Winters M P (1998). Tetrahedron Lett.

[16] Evans D A, Katz J L, West T R (1998). Tetrahedron Lett.

[17] Lam P Y S, Clark C G, Saubern S, Adams J, Winters M P, Chan D M T, Combs A (1998). Tetrahedron Lett.

[18] Qiao J, Lam P (2011). Synthesis.

[19] Lam P Y S (2016). Chan–Lam Coupling Reaction: Copper-promoted C–Element Bond Oxidative Coupling Reaction with Boronic Acids. Synthetic Methods in Drug Discovery.

[20] Vantourout J C, Miras H N, Isidro-Llobet A, Sproules S, Watson A J B (2017). J Am Chem Soc.

[21] Sukach V A, Tkachuk V M, Shoba V M, Pirozhenko V V, Rusanov E B, Chekotilo A A, Röschenthaler G-V, Vovk M V (2014). Eur J Org Chem.

[22] Sukach V A, Resetnic A A, Tkachuk V M, Lin Z, Kortz U, Vovk M V, Röschenthaler G-V (2015). Eur J Org Chem.

[23] Tkachuk V M, Sukach V A, Kovalchuk K V, Vovk M V, Nenajdenko V G (2015). Org Biomol Chem.

[24] Melnykov S V, Pataman A S, Dmytriv Y V, Shishkina S V, Vovk M V, Sukach V A (2017). Beilstein J Org Chem.

[25] Tkachuk V M, Melnykov S V, Vorobei A V, Sukach V A, Vovk M V (2019). Chem Heterocycl Compd.

[26] Zhou Y, Wang J, Gu Z, Wang S, Zhu W, Aceña J L, Soloshonok V A, Izawa K, Liu H (2016). Chem Rev.

[27] Wang J, Sánchez-Roselló M, Aceña J L, del Pozo C, Sorochinsky A E, Fustero S, Soloshonok V A, Liu H (2014). Chem Rev.

[28] Bassetto M, Ferla S, Pertusati F (2015). Future Med Chem.

[29] Gillis E P, Eastman K J, Hill M D, Donnelly D J, Meanwell N A (2015). J Med Chem.

[30] Zanatta N, Faoro D, da S. Fernandes L, Brondani P B, Flores D C, Flores A F C, Bonacorso H G, Martins M A P (2008). Eur J Org Chem.

[31] Sukach V A, Tkachuk V M, Rusanov E B, Röschenthaler G-V, Vovk M V (2012). Tetrahedron.

[32] Gerus I I, Vdovenko S I, Gorbunova M G, Kukhar' V P (1991). Chem Heterocycl Compd.

[33] Bonacorso H G, Martins M A P, Bittencourt S R T, Lourega R V, Zanatta N, Flores A F C (1999). J Fluorine Chem.

[34] Gorbunova M G, Gerus I I, Kukhar V P (2000). Synthesis.

[35] Farahat A A, Boykin D W (2015). Synth Commun.

[36] Hardouin Duparc V, Bano G L, Schaper F (2018). ACS Catal.

[37] Sanjeeva Rao K, Wu T-S (2012). Tetrahedron.

[38] Chua G N L, Wassarman K L, Sun H, Alp J A, Jarczyk E I, Kuzio N J, Bennett M J, Malachowsky B G, Kruse M, Kennedy A J (2019). ACS Med Chem Lett.

[39] James C A, DeRoy P, Duplessis M, Edwards P J, Halmos T, Minville J, Morency L, Morin S, Simoneau B, Tremblay M (2013). Bioorg Med Chem Lett.

[40] Jacobsen M F, Knudsen M M, Gothelf K V (2006). J Org Chem.

[41] Yue Y, Zheng Z-G, Wu B, Xia C-Q, Yu X-Q (2005). Eur J Org Chem.

[42] Tao L, Yue Y, Zhang J, Chen S-Y, Yu X-Q (2008). Helv Chim Acta.

[43] Bolshan Y, Batey R A (2010). Tetrahedron.

[44] Janíková K, Jedinák L, Volná T, Cankař P (2018). Tetrahedron.

[45] Bolshan Y, Batey R A (2008). Angew Chem, Int Ed.

[46] Jiao J-W, Bi H-Y, Zou P-S, Wang Z-X, Liang C, Mo D-L (2018). Adv Synth Catal.

[47] Chen C-H, Liu Q-Q, Ma X-P, Feng Y, Liang C, Pan C-X, Su G-F, Mo D-L (2017). J Org Chem.

[48] Ohata J, Minus M B, Abernathy M E, Ball Z T (2016). J Am Chem Soc.

[49] Carbery D R (2008). Org Biomol Chem.

[50] Gopalaiah K, Kagan H B (2011). Chem Rev.

[51] Liu S, Xu L (2018). Asian J Org Chem.

[52] Vantourout J C, Law R P, Isidro-Llobet A, Atkinson S J, Watson A J B (2016). J Org Chem.

[53] Coombs J R, Zhang L, Morken J P (2015). Org Lett.

[54] Kovalenko M, Yarmoliuk D V, Serhiichuk D, Chernenko D, Smyrnov V, Breslavskyi A, Hryshchuk O V, Kleban I, Rassukana Y, Tymtsunik A V (2019). Eur J Org Chem.

[55] Hill M, Movassaghi M (2007). Synthesis.

[56] Hansen A L, Skrydstrup T (2005). J Org Chem.

[57] Song F, Snook J H, Foxman B M, Snider B B (1998). Tetrahedron.

