# Supplementary material for: Chan–Evans–Lam N1-(het)arylation and N1-alkеnylation of 4-fluoroalkylpyrimidin-2(1H)-ones
Source: Beilstein J Org Chem. 2020 Sep 17;16:2304–13. doi: 10.3762/bjoc.16.191 (PMC7509380; doi:10.3762/bjoc.16.191)

BEILSTEIN JOURNAL OF ORGANIC CHEMISTRY

Supporting Information

for

Chan–Evans–Lam *N*1-(het)arylation and *N*1-alkenylation of 4-fluoroalkylpyrimidin-2(1*H*)-ones

Viktor M. Tkachuk, Oleh O. Lukianov, Mykhailo V. Vovk, Isabelle Gillaizeau and Volodymyr A. Sukach

Beilstein J. Org. Chem. **2020,** *16,* 2304–2313. doi:10.3762/bjoc.16.191

Experimental procedures, characterization data, copies of the ^{1}H and ^{13}C NMR spectra

License and Terms: This is a supporting information file under the terms of the Creative Commons Attribution License (https://creativecommons.org/licenses/by/4.0). Please note that the reuse, redistribution and reproduction in particular requires that the authors and source are credited.
The license is subject to the *Beilstein Journal of Organic Chemistry* terms and conditions: (https://www.beilstein-journals.org/bjoc)

Content

1. General methods……………………………………………………………………………….S2

2. General procedure 1 (GP1) for the synthesis of compounds 3a–w, 5a and 9a–g by Chan–Evans–Lam reaction of pyrimidin-2(1*H*)-ones 1a–h with boronic acids 2a–w and 4………………………………………………………………………………………………… S2

3. General procedure 2 (GP2) for the synthesis of compounds 3a,g,q,s, 5a–h and 8 by Chan–Evans–Lam reaction of pyrimidin-2(1*H*)-one (1a) with boronic acid pinacol esters 6a–d and 7a–h……………………………………………………………………………..S13

4. References……………………………………………………………………………………...S19

5. Copies of the ^{1}H and ^{13}C NMR spectra………………………………………………...S19

1. General methods

All chemicals (including starting pyrimidin-2(1H)-ones **1a–c,e–h**, boronic acids **2a–w**, boronic acid pinacol esters **6a**–**d** and **7a–h**) were obtained from Enamine Ltd. and used without further purification. All solvents were purified by standard methods. Melting points are uncorrected. ^{19}F NMR, ^{1}H NMR and ^{13}C NMR spectra were recorded on a Varian VXR-300, Varian Mercury-400 or Bruker Avance DRX-500 spectrometers with TMS or CCl_3F as an internal standard. Multiplets were assigned as s (singlet), d (doublet), t (triplet), dd (doublet of doublet), q (quartet), m (multiplet) and br s (broad singlet). LC-MS spectra were recorded on an Agilent 1100 Series high performance liquid chromatograph equipped with a diode matrix with an Agilent LC\MSD SL mass selective detector. Mass spectrometric detections of samples were performed with an Infinity 1260 UHPLC system (Agilent Technologies, Waldbronn, Germany) coupled to an 6224 Accurate Mass TOF LC/MS system (Agilent Technologies, Singapore). Infrared (IR) spectra were recorded on a Bruker Vertex 70 (ATR) or FT-IR spectrometer. The samples were prepared as neat fine powders and the wave numbers are reported in cm^{-1}. UV absorbance data were measured on a Shimadzu UV-3100 spectrophotometer. Fluorescence spectra were determined using a Solar CM-2203 fluorescence spectrophotometer. Compound **1d** was prepared according to the literature procedure [1].

2. General procedure 1 (GP1) for the synthesis of compounds 3a–w, 5a and 9a–g by Chan–Evans–Lam reaction of pyrimidin-2(1*H*)-ones 1a–h with boronic acids 2a–w and 4

To a suspension of compound **1a–h**, corresponding boronic acid **2a–w, 4** and copper(II) acetate monohydrate (599 mg, 3 mmol, 1 equiv) in acetonitrile (20 mL) pyridine (0.475 g, 0.48 mL, 6 mmol, 2 equiv) was added. The mixture was vigorously stirred with an open air condenser at room temperature for 48 h and then heated at 80 °C for 8 h. After cooling to room temperature the mixture was filtered, the solid material was washed with acetonitrile (2 × 10 mL). The combined filtrates were concentrated under reduced pressure, the residue was treated with 2 N ammonium hydroxide solution (20 mL) and was extracted with ethyl acetate (2 × 30 mL). The organic layers were combined, washed with brine (2 × 30 mL), dried over anhydrous sodium sulfate, filtered and the solvent was evaporated under reduced pressure. The obtained residue was treated as specified below.

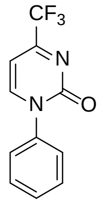

1-Phenyl-4-(trifluoromethyl)pyrimidin-2(1*H*)-one (3a). Following the GP1, using compound **1a** (492 mg, 3 mmol, 1 equiv) and phenylboronic acid **2a** (366 mg, 3 mmol, 1 equiv). The obtained residue was refluxed with methyl *tert*-butyl ether (20 mL), cooled and filtered off. White solid (658 mg, 90 %). Mp 162-164 °C. **IR** (neat): ν_{max} 3060, 1670, 1530, 1456, 1325, 1308, 1204, 1155, 1055, 790, 696. **^{1}H NMR** (400 MHz, DMSO-d_6): δ 8.60 (d, *J* = 6.6 Hz, 1H), 7.55 (bs, 5H), 6.95 (d, *J* = 6.6 Hz, 1H). **^{13}C NMR** (125 MHz, DMSO-d_6): δ 162.5 (q, *J* = 35.3 Hz), 155.1, 154.5, 140.3, 129.7, 129.6, 126.9, 120.0 (q, *J* = 277.6 Hz), 99.5. **^{19}F NMR** (376 MHz, DMSO-d_6): δ –70.97 (s). **HRMS** (ESI+): calcd for $C_{11}H_7F_3N_2O$ $[M+H]^+$: 241.0583, found 241.0582.

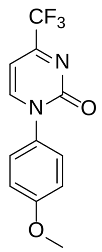

1-(4-Methoxyphenyl)-4-(trifluoromethyl)pyrimidin-2(1*H*)-one (3b). Following the GP1, using compound **1a** (492 mg, 3 mmol, 1 equiv) and 4-methoxyphenylboronic acid **2b** (456 mg, 3 mmol, 1 equiv). The obtained residue was refluxed in methyl *tert*-butyl ether (20 mL), cooled and filtered off. White solid (699 mg, 85 %). Mp 127-129 °C. **IR** (neat): ν_{max} 3030, 1670, 1513, 1464, 1316, 1262, 1208, 1170, 1027, 836, 798. **^{1}H NMR** (400 MHz, DMSO-d_6): δ 8.55 (d, *J* = 6.6 Hz, 1H), 7.46 (d, *J* = 8.8 Hz, 2H), 7.08 (d, *J* = 8.8 Hz, 2H), 6.92 (d, *J* = 6.6 Hz, 1H), 3.81 (s, 3H). **^{13}C NMR** (126 MHz, DMSO-d_6): δ 162.2 (q, *J* = 35.5 Hz), 160.0, 155.3, 154.7, 133.1, 128.1, 120.0 (q, *J* = 277.6 Hz), 114.8, 99.4, 56.0. **^{19}F NMR** (376 MHz, DMSO-d_6): δ –71.01 (s). **HRMS** (ESI+): calcd for $C_{12}H_9F_3N_2O_2$ $[M+H]^+$: 271.0689, found 271.0688.

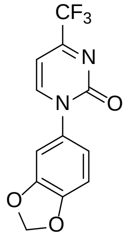

1-(Benzo[*d*][1,3]dioxol-5-yl)-4-(trifluoromethyl)pyrimidin-2(1*H*)-one (3c). Following the GP1, using compound **1a** (492 mg, 3 mmol, 1 equiv) and benzo[*d*][1,3]dioxol-5-ylboronic acid **2c** (498 mg, 3 mmol, 1 equiv). The obtained residue was refluxed in methyl *tert*-butyl ether (20 mL), cooled and filtered off. Light brown solid (767 mg, 90%). Mp 168-170 °C. **IR** (neat): ν_{max} 3102, 2905, 1669, 1527, 1494, 1450, 1199, 1145, 1038, 933, 806. **^{1}H NMR** (400 MHz, DMSO-d_6): δ 8.54 (d, *J* = 6.6 Hz, 1H), 7.18 (s, 1H), 7.06 (d, *J* = 8.2 Hz, 1H), 7.00 (d, *J* = 8.3 Hz, 1H), 6.92 (d, *J* = 6.7 Hz, 1H), 6.13 (s, 2H). **^{13}C NMR** (126 MHz, DMSO-d_6): δ 162.3 (d, *J* = 35.2 Hz), 155.4, 154.6,

148.2, 147.9, 134.1, 120.0 (d, J = 277.5 Hz), 120.6, 108.7, 108.2, 102.5, 99.3. **^{19}F NMR** (376 MHz, DMSO-d_6): δ –70.93 (s). **HRMS** (ESI+): calcd for $C_{12}H_7F_3N_2O_3$ [M+H]$^+$: 285.0482, found 285.0484.

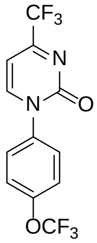

1-(4-(Trifluoromethoxy)phenyl)-4-(trifluoromethyl)pyrimidin-2(1*H*)-one (3d). Following the GP1, using compound **1a** (492 mg, 3 mmol, 1 equiv) and (4-(trifluoromethoxy)phenyl)boronic acid **2d** (618 mg, 3 mmol, 1 equiv). The obtained residue was refluxed in methyl *tert*-butyl ether (20 mL), cooled and filtered off. White solid (651 mg, 67%). Mp 147-148 °C. **IR** (neat): ν_{max} 3103, 3036, 1679, 1528, 1505, 1466, 1314, 1212, 1152, 1056, 803. **^{1}H NMR** (400 MHz, DMSO-d_6): δ 8.65 (d, J = 6.7 Hz, 1H), 7.72 (d, J = 8.7 Hz, 2H), 7.58 (d, J = 8.4 Hz, 2H), 6.99 (d, J = 6.7 Hz, 1H). **^{13}C NMR** (126 MHz, DMSO-d_6): δ 162.69 (q, J = 35.5 Hz), 155.08, 154.39, 148.84, 139.09, 129.35, 122.32, 120.49 (q, J = 257.1 Hz), 119.97 (q, J = 277.6 Hz), 99.65. **^{19}F NMR** (376 MHz, DMSO-d_6): δ –57.43 (s, 3F), -71.03 (s, 3F). **HRMS** (ESI+): calcd for $C_{12}H_6F_6N_2O_2$ [M+H]$^+$: 325.0406, found 325.0404.

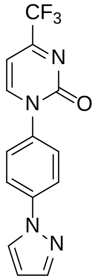

1-[4-(1*H*-Pyrazol-1-yl)phenyl]-4-(trifluoromethyl)pyrimidin-2(1*H*)-one (3e). Following the GP1, using compound **1a** (492 mg, 3 mmol, 1 equiv) and (4-(1*H*-pyrazol-1-yl)phenyl)boronic acid **2e** (564 mg, 3 mmol, 1 equiv). The obtained residue was refluxed in methyl *tert*-butyl ether, cooled and filtered off. White solid (753 mg, 82 %). Mp 226-227 °C. **IR** (neat): ν_{max} 3119, 3072, 1676, 1623, 1533, 1512, 1455, 1311, 1207, 1141, 1061, 936, 812, 744. **^{1}H NMR** (400 MHz, DMSO-d_6): δ 8.62 (d, J = 4.7 Hz, 1H), 8.56 (s, 1H), 8.01 (d, J = 6.6 Hz, 2H), 7.80 (s, 1H), 7.69 (d, J = 6.6 Hz, 2H), 6.95 (d, J = 4.9 Hz, 1H), 6.59 (s, 1H). **^{13}C NMR** (126 MHz, DMSO-d_6): δ 162.5 (q, J = 35.3 Hz), 155.10, 154.48, 142.07, 140.37, 137.82, 128.62, 128.26, 119.24, 120.00 (q, J = 277.8 Hz), 108.84, 99.62. **^{19}F NMR** (376 MHz, DMSO-d_6): δ –70.91 (s). **HRMS** (ESI+): calcd for $C_{14}H_9F_3N_4O$ [M+H]$^+$: 307.0801, found 307.0798.

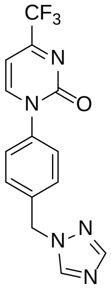

1-(4-((1*H*-1,2,4-Triazol-1-yl)methyl)phenyl)-4-(trifluoromethyl)pyrimidin-2(1*H*)-one (3f). Following the GP1, using compound **1a** (492 mg, 3 mmol, 1 equiv) and (4-((1*H*-1,2,4-triazol-1-yl)methyl)phenyl)boronic acid **2f** (619 mg, 3 mmol, 1 equiv). The obtained residue was refluxed in methyl *tert*-butyl ether (20 mL), cooled and filtered off. White solid (685 mg, 70 %). Mp 163-165 °C. **IR** (neat): ν_{max} 3092, 3035, 1675, 1509, 1465, 1316, 1215, 1151, 1059, 805. **^{1}H NMR** (400 MHz, DMSO-d_6): δ 8.72 (s, 1H), 8.59 (d, J = 6.6 Hz, 1H), 8.01 (s, 1H), 7.54 (d, J = 8.0 Hz, 2H), 7.44 (d, J = 8.0 Hz, 2H), 6.95 (d, J = 6.6 Hz, 1H), 5.51 (s, 2H). **^{13}C NMR** (150 MHz, DMSO-d_6): δ 162.5 (q, J = 35.4 Hz), 155.0, 154.4, 152.3, 144.9, 139.7, 137.9, 129.1, 127.2, 119.9 (q, J = 277.5 Hz), 99.5, 51.9. **^{19}F NMR** (376 MHz, DMSO-d_6): δ –70.98 (s). **HRMS** (ESI+): calcd for $C_{14}H_{10}F_3N_5O$ $[M+H]^+$: 322.0910, found 322.0909.

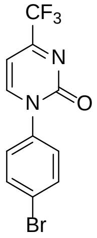

1-(4-Bromophenyl)-4-(trifluoromethyl)pyrimidin-2(1*H*)-one (3g). Following the GP1, using compound **1a** (492 mg, 3 mmol, 1 equiv) and (4-bromophenyl)boronic acid **2g** (600 mg, 3 mmol, 1 equiv). The obtained residue was refluxed in methyl *tert*-butyl ether (20 mL), cooled and filtered off. White solid (632 mg, 66 %). Mp 164-166 °C. **IR** (neat): ν_{max} 3081, 3065, 1672, 1516, 1433, 1304, 1152, 1063, 846. **^{1}H NMR** (400 MHz, DMSO-d_6): δ 8.57 (d, J = 6.6 Hz, 1H), 7.76 (d, J = 8.4 Hz, 2H), 7.53 (d, J = 8.6 Hz, 2H), 6.94 (d, J = 6.7 Hz, 1H). **^{13}C NMR** (126 MHz, DMSO-d_6): δ 162.6 (q, J = 35.4 Hz), 158.6, 154.9, 154.3, 139.5, 132.6, 129.3, 122.8, 120.0 (q, J = 277.6 Hz), 99.6. **^{19}F NMR** (376 MHz, DMSO-d_6): δ –70.59 (s). **HRMS** (ESI+): calcd for $C_{11}H_6BrF_3N_2O$ $[M+H]^+$: 318.9689, found 318.9693.

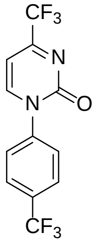

4-(Trifluoromethyl)-1-(4-(trifluoromethyl)phenyl)pyrimidin-2(1*H*)-one (3h). Following the GP1, using compound **1a** (492 mg, 3 mmol, 1 equiv) and (4-(trifluoromethyl)phenyl)boronic acid **2h** (570 mg, 3 mmol, 1 equiv). The obtained residue was refluxed in methyl *tert*-butyl ether (20 mL), cooled and filtered off. White solid (795 mg, 86%). Mp 165-166 °C. **IR** (neat): ν_{max} 3116, 3056, 3037, 1674, 1532, 1461, 1333, 1310, 1208, 1168, 1129, 1072, 856, 823, 790. **^{1}H NMR** (400 MHz, DMSO-d_6): δ 8.63 (d, J = 6.3 Hz, 1H), 7.95 (d, J = 8.2 Hz, 2H), 7.82 (d, J = 8.1 Hz, 2H), 6.99 (d, J = 6.4 Hz, 1H). **^{13}C NMR** (126 MHz, DMSO-d_6): δ 162.84 (q, J = 35.3 Hz), 154.9, 154.2, 143.5, 130.0 (q,

J = 32.2 Hz), 128.3, 126.9, 124.3 (q, *J* = 277.2 Hz), 120.0 (q, *J* = 289.8 Hz), 99.8. **^{19}F NMR** (376 MHz, DMSO-*d*$_6$): δ -61.70 (s, 3F), -71.02 (s, 3F). **HRMS** (ESI+): calcd for $C_{12}H_6F_6N_2O$ [M+H]$^+$: 309.0457, found 309.0462.

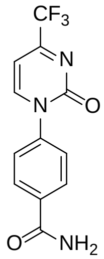

4-(2-Oxo-4-(trifluoromethyl)pyrimidin-1(2*H*)-yl)benzamide (3i). Following the GP1, using compound **1a** (492 mg, 3 mmol, 1 equiv) and (4-carbamoylphenyl)boronic acid **2i** (495 mg, 3 mmol, 1 equiv). The obtained residue was purified by reverse phase HPLC method (eluent CH_3CN/H_2O). White solid (365 mg, 43%). Mp >260 °C. **IR** (neat): ν_{max} 3385, 3183, 1681, 1645, 1459, 1305, 1208, 1152, 1123, 1047, 790. **^{1}H NMR** (400 MHz, DMSO-*d*$_6$): δ 8.62 (d, *J* = 6.7 Hz, 1H), 8.12 (s, 1H), 8.02 (d, *J* = 8.4 Hz, 2H), 7.64 (d, *J* = 8.4 Hz, 2H), 7.52 (s, 1H), 6.97 (d, *J* = 6.7 Hz, 1H). **^{13}C NMR** (126 MHz, DMSO-*d*$_6$): δ 167.4, 162.6 (q, *J* = 35.4 Hz), 154.9, 154.2, 142.3, 135.4, 128.8, 126.9, 119.9 (q, *J* = 277.6 Hz), 99.6. **^{19}F NMR** (376 MHz, DMSO-*d*$_6$): δ –70.93 (s). **HRMS** (ESI+): calcd for $C_{12}H_8F_3N_3O_2$ [M+H]$^+$: 284.0642, found 284.0643.

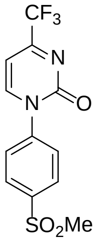

1-(4-(Methylsulfonyl)phenyl)-4-(trifluoromethyl)pyrimidin-2(1*H*)-one (3j). Following the GP1, using compound **1a** (492 mg, 3 mmol, 1 equiv) and (4-(methylsulfonyl)phenyl)boronic acid **2j** (610 mg, 3 mmol, 1 equiv). The obtained residue was refluxed in methyl *tert*-butyl ether (20 mL), cooled and filtered off. White solid (553 mg, 57 %). Mp 220-222 °C. **IR** (neat): ν_{max} 3107, 1687, 1532, 1460, 1306, 1201, 1147, 1057, 792. **^{1}H NMR** (400 MHz, DMSO-*d*$_6$): δ 8.66 (d, *J* = 6.7 Hz, 1H), 8.12 (d, *J* = 8.4 Hz, 2H), 7.86 (d, *J* = 8.4 Hz, 2H), 7.03 (d, *J* = 6.7 Hz, 1H), 3.32 (s, 3H). **^{13}C NMR** (125 MHz, DMSO-*d*$_6$): δ 162.9 (q, *J* = 35.6 Hz), 154.8, 154.2, 144.2, 141.8, 128.6, 128.3, 119.9 (q, *J* = 277.8 Hz), 99.9, 43.8. **^{19}F NMR** (376 MHz, DMSO-*d*$_6$): δ –70.99 (s). **HRMS** (ESI+): calcd for $C_{12}H_9F_3N_2O_3S$ [M+H]$^+$: 319.0359, found 319.0362.

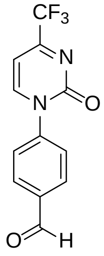

4-(2-Oxo-4-(trifluoromethyl)pyrimidin-1(2*H*)-yl)benzaldehyde (3k). Following the GP1, using compound **1a** (492 mg, 3 mmol, 1 equiv) and (4-formylphenyl)boronic acid **2k** (450 mg, 3 mmol, 1 equiv). The obtained residue was refluxed in methyl *tert*-butyl ether (20 mL), cooled and filtered off. White solid (572 mg, 70 %). Mp 160-162

°C. **IR** (neat): ν_{max} 3023, 1716, 1683, 1523, 1456, 1313, 1216, 1152, 1058, 831, 815. **^{1}H NMR** (400 MHz, DMSO-d_6): δ 10.10 (s, 1H), 8.63 (d, *J* = 6.6 Hz, 1H), 8.09 (d, *J* = 8.1 Hz, 2H), 7.80 (d, *J* = 8.1 Hz, 2H), 6.99 (d, *J* = 6.6 Hz, 1H). **^{13}C NMR** (150 MHz, DMSO-d_6): δ 192.8, 162.7 (q, *J* = 35.4 Hz), 154.7, 154.1, 144.7, 136.7, 130.7, 127.9, 119.9 (q, *J* = 277.8 Hz), 99.7. **^{19}F NMR** (376 MHz, DMSO-d_6): δ –71.02 (s). **HRMS** (ESI+): calcd for $C_{12}H_7F_3N_2O_2$ $[M+H]^+$: 269.0534, found 269.0535.

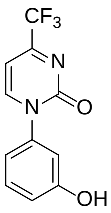

1-(3-Hydroxyphenyl)-4-(trifluoromethyl)pyrimidin-2(1*H*)-one (3l). Following the GP1, using compound **1a** (492 mg, 3 mmol, 1 equiv) and (3-hydroxyphenyl)boronic acid **2l** (414 mg, 3 mmol, 1 equiv). The obtained residue was purified by reverse phase HPLC method (eluent CH_3CN/H_2O). Slightly brown solid (576 mg, 75 %). Mp 192-195 °C. **IR** (neat): ν_{max} 3342, 3038, 1657, 1615, 1587, 1457, 1313, 1214, 1155, 1067, 963. **^{1}H NMR** (400 MHz, DMSO-d_6): δ 9.98 (s, 1H), 8.54 (d, *J* = 6.3 Hz, 1H), 7.33 (t, *J* = 7.4 Hz, 1H), 6.91 (bs, 4H). **^{13}C NMR** (125 MHz, DMSO-d_6): δ 162.4 (q, *J* = 35.3 Hz), 158.4, 155.0, 154.3, 141.2, 130.6, 120.0 (q, *J* = 276.1 Hz), 117.2, 116.7, 114.0, 99.4. **^{19}F NMR** (376 MHz, DMSO-d_6): δ –70.33 (s). **HRMS** (ESI+): calcd for $C_{11}H_7F_3N_2O_2$ $[M+H]^+$: 257.0532, found 257.0536.

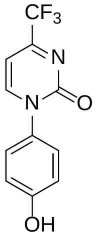

1-(4-Hydroxyphenyl)-4-(trifluoromethyl)pyrimidin-2(1*H*)-one (3m). Following the GP1, using compound **1a** (492 mg, 3 mmol, 1 equiv) and (4-hydroxyphenyl)boronic acid **2m** (414 mg, 3 mmol, 1 equiv). The obtained residue was purified by reverse phase HPLC method (eluent CH_3CN/H_2O). Slightly brown solid (668 mg, 87 %). Mp 213-215 °C. **IR** (neat): ν_{max} 3335, 3025, 1664, 1620, 1454, 1323, 1216, 1152, 1073, 957. **^{1}H NMR** (400 MHz, DMSO-d_6): δ 9.94 (s, 1H), 8.53 (d, *J* = 5.0 Hz, 1H), 7.32 (d, *J* = 7.2 Hz, 2H), 6.88 (bs, 3H). **^{13}C NMR** (150 MHz, DMSO-d_6): δ 162.0 (q, *J* = 35.2 Hz), 158.4, 155.3, 154.6, 131.6, 127.9, 120.0 (d, *J* = 277.5 Hz), 115.9, 99.3. **^{19}F NMR** (376 MHz, DMSO-d_6): δ –70.42 (s). **HRMS** (ESI+): calcd for $C_{11}H_7F_3N_2O_2$ $[M+H]^+$: 257.0532, found 257.0535.

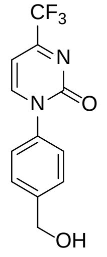

1-(4-(Hydroxymethyl)phenyl)-4-(trifluoromethyl)pyrimidin-2(1*H*)-one (3n). Following the GP1, using compound **1a** (492 mg, 3 mmol, 1 equiv) and (4-(hydroxymethyl)phenyl)boronic acid **2n** (456 mg, 3 mmol, 1 equiv). The obtained residue was refluxed in methyl *tert*-butyl ether (20 mL), cooled and filtered off. White solid (486 mg, 60%). Mp 145-146 °C. **IR** (neat): ν_{max} 3288, 3030, 2920, 2875, 1668, 1529, 1463, 1313, 1210, 1133, 1056, 798. **^{1}H NMR** (400 MHz, DMSO-*d*6): δ 8.57 (d, *J* = 6.6 Hz, 1H), 7.64 – 7.38 (m, 5H), 6.94 (d, *J* = 6.7 Hz, 1H), 5.35 (t, *J* = 5.7 Hz, 1H), 4.57 (d, *J* = 5.6 Hz, 3H). **^{13}C NMR** (126 MHz, DMSO-*d*6): δ 162.3 (q, *J* = 35.2 Hz), 155.1, 154.4, 144.2, 138.7, 127.4, 126.5, 119.94 (q, *J* = 277.8 Hz), 99.5, 62.7. **^{19}F NMR** (376 MHz, DMSO-*d*6): δ –70.42 (s). **HRMS** (ESI+): calcd for $C_{12}H_9F_3N_2O_2$ $[M+H]^+$: 271.0689, found 271.0692.

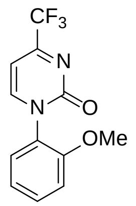

1-(2-Methoxyphenyl)-4-(trifluoromethyl)pyrimidin-2(1*H*)-one (3o). Following the GP1, using compound **1a** (492 mg, 3 mmol, 1 equiv) and (2-methoxyphenyl)boronic acid **2o** (456 mg, 3 mmol, 1 equiv). The obtained residue was refluxed in methyl *tert*-butyl ether (20 mL), cooled and filtered off. White solid (421 mg, 52 %). Mp 156-158 °C. **IR** (neat): ν_{max} 3104, 1673, 1531, 1502, 1450, 1296, 1202, 1150, 759. **^{1}H NMR** (400 MHz, DMSO-*d*6): δ 8.49 (d, *J* = 6.1 Hz, 1H), 7.61 – 7.35 (m, 2H), 7.22 (d, *J* = 8.0 Hz, 1H), 7.07 (t, *J* = 7.2 Hz, 1H), 6.89 (d, *J* = 6.1 Hz, 1H), 3.75 (s, 3H). **^{13}C NMR** (100 MHz, DMSO-*d*6): δ 162.7 (q, *J* = 35.5 Hz), 156.2, 154.0, 131.5, 128.6, 128.5, 121.1, 119.9 (q, *J* = 277.9 Hz), 113.1, 99.2, 56.5. **^{19}F NMR** (376 MHz, DMSO-*d*6): δ –71.03 (s). **HRMS** (ESI+): calcd for $C_{12}H_9F_3N_2O_2$ $[M+H]^+$: 271.0689, found 271.0694.

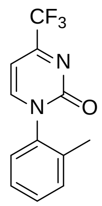

1-(*o*-Tolyl)-4-(trifluoromethyl)pyrimidin-2(1*H*)-one (3p). Following the GP1, using compound **1a** (492 mg, 3 mmol, 1 equiv) and *o*-tolylboronic acid **2p** (612 mg, 4.5 mmol, 1.5 equiv). The obtained residue was refluxed in methyl *tert*-butyl ether (20 mL), cooled and filtered off. White solid (267 mg, 35 %). Mp 145-147 °C. **IR** (neat): ν_{max} 3053, 1672, 1542, 1452, 1330, 1312, 1206, 1147, 1060, 792, 685. **^{1}H NMR** (400 MHz, DMSO-*d*6): 8.56 (d, *J* = 6.4 Hz, 1H), 7.56 – 7.27 (bs, 4H), 6.98 (d, *J* = 6.4 Hz, 1H), 2.10 (s, 3H). **^{13}C NMR** (100 MHz, DMSO-*d*6): δ 162.8 (q, *J* = 35.2 Hz), 155.4, 154.0, 139.5, 134.7, 131.4, 130.0, 127.7, 127.6, 120.0 (q, *J* = 276.3 Hz), 99.6, 17.4. **^{19}F NMR** (376 MHz, DMSO-*d*6): δ –70.88 (s). **HRMS** (ESI+): calcd for $C_{12}H_9F_3N_2O$ $[M+H]^+$: 255.0740, found 255.0740.

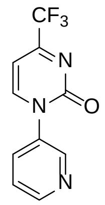

1-(Pyridin-3-yl)-4-(trifluoromethyl)pyrimidin-2(1*H*)-one (3q). Following the GP1, using compound **1a** (492 mg, 3 mmol, 1 equiv) and pyridin-3-ylboronic acid **2q** (562 mg, 4.5 mmol, 1.5 equiv). The obtained residue was recrystallized from toluene. White solid (94 mg, 13 %). Mp 152-154 °C. **IR** (neat): ν_{max} 3113, 1676, 1533, 1459, 1305, 1156, 1062, 793, 707. **^{1}H NMR** (400 MHz, DMSO-d_6): 8.77 (s, 1H), 8.74 – 8.64 (m, 2H), 8.05 (d, *J* = 7.4 Hz, 1H), 7.65 – 7.55 (m, 1H), 7.02 (d, *J* = 6.6 Hz, 1H). **^{13}C NMR** (125 MHz, DMSO-d_6): δ 162.90 (q, *J* = 35.2 Hz), 155.1, 154.4, 150.5, 147.7, 136.9, 135.0, 124.4, 120.0 (q, *J* = 276.2 Hz), 99.8. **^{19}F NMR** (376 MHz, DMSO-d_6): δ –71.08 (s). **HRMS** (ESI+): calcd for $C_{10}H_6F_3N_3O$ $[M+H]^+$: 242.0536, found 242.0537.

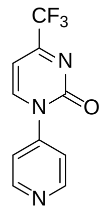

1-(Pyridin-4-yl)-4-(trifluoromethyl)pyrimidin-2(1*H*)-one (3r). Following the GP1, using compound **1a** (492 mg, 3 mmol, 1 equiv) and pyridin-4-ylboronic acid **2r** (562 mg, 4.5 mmol, 1.5 equiv). The obtained residue was recrystallized from toluene. White solid (123 mg, 17 %). Mp 135-137 °C. **IR** (neat): ν_{max} 3110, 1679, 1524, 1453, 1335, 1307, 1151, 1057, 798, 704. **^{1}H NMR** (400 MHz, DMSO-d_6): δ 8.80 (bs, 2H), 8.66 (d, *J* = 6.8 Hz, 1H), 7.66 (d, *J* = 4.5 Hz, 2H), 7.04 (d, *J* = 6.8 Hz, 1H). **^{13}C NMR** (150 MHz, DMSO-d_6): δ 162.9 (q, *J* = 35.5 Hz), 154.4, 153.7, 151.4, 147.2, 121.8, 119.9 (q, *J* = 276.2 Hz), 99.9. **^{19}F NMR** (376 MHz, DMSO-d_6): δ –71.07 (s). **HRMS** (ESI+): calcd for $C_{10}H_6F_3N_3O$ $[M+H]^+$: 242.0536, found 242.0541.

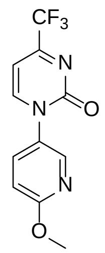

1-(6-Methoxypyridin-3-yl)-4-(trifluoromethyl)pyrimidin-2(1*H*)-one (3s). Following the GP1, using compound **1a** (492 mg, 3 mmol, 1 equiv) and (6-methoxypyridin-3-yl)boronic acid **2s** (700 mg, 4.5 mmol, 1.5 equiv). The obtained residue was recrystallized from toluene. White solid (372 mg, 45 %). Mp 128-130 °C. **IR** (neat): ν_{max} 3104, 1671, 1529, 1501, 1455, 1323, 1312, 1207, 1154, 1022, 806. **^{1}H NMR** (400 MHz, DMSO-d_6): δ 8.62 (d, *J* = 6.4 Hz, 1H), 8.34 (s, 1H), 7.93 (d, *J* = 7.6 Hz, 1H), 7.15 – 6.85 (m, 2H), 3.91 (s, 3H). **^{13}C NMR** (125 MHz, DMSO-d_6): δ 163.9, 162.6 (q, *J* = 35.3 Hz), 155.3, 154.6, 144.9, 138.2, 131.3, 120.0 (q, *J* = 276.3 Hz), 111.0, 99.7, 54.2. **^{19}F NMR** (376

MHz, DMSO-d_6): δ –70.48 (s). **HRMS** (ESI+): calcd for $C_{11}H_8F_3N_3O_2$ [M+H]$^+$: 272.0641, found 272.0644.

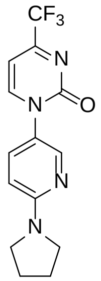

1-(6-(Pyrrolidin-1-yl)pyridin-3-yl)-4-(trifluoromethyl)pyrimidin-2(1*H*)-one (3t). Following the GP, using compound **1a** (492 mg, 3 mmol, 1 equiv) and (6-(pyrrolidin-1-yl)pyridin-3-yl)boronic acid **2t** (878 mg, 4.5 mmol, 1.5 equiv). The obtained residue was recrystallized from toluene. Yellow solid (595 mg, 63 %). Mp 230-232°C. **IR** (neat): v_{max} 3093, 2865, 1692, 1615, 1520, 1463, 1306, 1203, 1147, 1057, 806, 790. **^{1}H NMR** (400 MHz, DMSO-d_6): δ 8.56 (d, *J* = 6.6 Hz, 1H), 8.16 (d, *J* = 1.5 Hz, 1H), 7.66 (d, *J* = 10.5 Hz, 1H), 6.93 (d, *J* = 6.6 Hz, 1H), 6.53 (d, *J* = 9.0 Hz, 1H), 3.42 (bs, 4H), 1.96 (bs, 4H). **^{13}C NMR** (125 MHz, DMSO-d_6): δ 162.2 (q, *J* = 35.6 Hz), 157.0, 155.3, 154.8, 145.5, 135.5, 125.9, 120.0 (q, *J* = 277.1 Hz), 106.1, 99.5, 47.1, 25.5. **^{19}F NMR** (376 MHz, DMSO-d_6): δ –70.89 (s). **HRMS** (ESI+): calcd for $C_{14}H_{13}F_3N_4O$ [M+H]$^+$: 311.1114, found 311.1117.

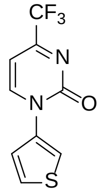

1-(Thiophen-3-yl)-4-(trifluoromethyl)pyrimidin-2(1*H*)-one (3u). Following the GP1, using compound **1a** (492 mg, 3 mmol, 1 equiv) and thiophen-3-ylboronic acid **2u** (585 mg, 4.5 mmol, 1.5 equiv). The obtained residue was recrystallized from toluene. White solid (352 mg, 47 %). Mp 148-150 °C. **IR** (neat): v_{max} 3095, 1663, 1514, 1458, 1338, 1299, 1204, 1146, 1052, 795. **^{1}H NMR** (400 MHz, DMSO-d_6): δ 8.64 (d, *J* = 6.7 Hz, 1H), 7.94 (br s, 1H), 7.69 (dd, *J* = 5.0, 3.2 Hz, 1H), 7.38 (d, *J* = 0.9 Hz, 1H), 6.94 (d, *J* = 6.7 Hz, 1H). **^{13}C NMR** (125 MHz, DMSO-d_6): δ 162.1 (q, *J* = 35.6 Hz), 154.8, 153.8, 137.8, 126.9, 125.4, 122.6, 120.0 (q, *J* = 276.3 Hz), 99.6. **^{19}F NMR** (376 MHz, DMSO-d_6): δ –70.96 (s). **HRMS** (ESI+): calcd for $C_9H_5F_3N_2OS$ [M+H]$^+$: 247.0147, found 247.0155.

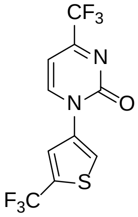

4-(Trifluoromethyl)-1-(5-(trifluoromethyl)thiophen-3-yl)pyrimidin-2(1*H*)-one (3v). Following the GP1, using compound **1a** (492 mg, 3 mmol, 1 equiv) and (5-(trifluoromethyl)thiophen-3-yl)boronic acid **2v** (896 mg, 4.5 mmol, 1.5 equiv). The obtained residue was purified by reverse phase HPLC method (eluent CH_3CN/H_2O). White solid (191 mg, 20 %). Mp 142-144 °C. **IR** (neat): v_{max} 3082,

1684, 1471, 1293, 1220, 1161, 1138, 801. **^{1}H NMR** (400 MHz, DMSO-*d*$_6$): 8.71 (d, *J* = 6.8 Hz, 1H), 8.33 (d, *J* = 1.6 Hz, 1H), 8.03 (d, *J* = 0.9 Hz, 1H), 7.01 (d, *J* = 6.8 Hz, 1H). **^{13}C NMR** (125 MHz, DMSO-*d*$_6$): δ 162.6 (q, *J* = 35.4 Hz), 154.9, 153.8, 137.1, 129.1 (q, *J* = 38.8 Hz), 128.6 (q, *J* = 3.7 Hz), 127.8, 122.6 (q, *J* = 267.3 Hz), 119.9 (q, *J* = 276.3 Hz), 99.8. **^{19}F NMR** (376 MHz, DMSO-*d*$_6$): δ –55.26 (s, 3F), –71.04 (s, 3F). **HRMS** (ESI+): calcd for $C_{10}H_4F_6N_2OS$ $[M+H]^+$: 315.0021, found 315.0024.

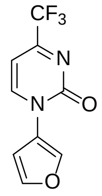

1-(Furan-3-yl)-4-(trifluoromethyl)pyrimidin-2(1*H*)-one (3w). Following the GP1, using compound **1a** (492 mg, 3 mmol, 1 equiv) and furan-3-ylboronic acid **2w** (512 mg, 4.5 mmol, 1.5 equiv). The obtained residue was recrystallized from toluene. Beige solid (97 mg, 14 %). Mp 138-140 °C. **IR** (neat): ν_{max} 3122, 1664, 1526, 1460, 1338, 1212, 1151, 1065, 793. **^{1}H NMR** (400 MHz, DMSO-*d*$_6$): 8.83 (d, *J* = 6.8 Hz, 1H), 8.47 (s, 1H), 7.85 (s, 1H), 7.10 (s, 1H), 7.02 (d, *J* = 6.8 Hz, 1H). **^{13}C NMR** (100 MHz, DMSO-*d*$_6$): δ 160.9 (q, *J* = 35.3 Hz), 152.8, 152.5, 143.9, 138.1, 127.1, 120.0 (q, *J* = 275.8 Hz), 106.9, 100.0. **^{19}F NMR** (376 MHz, DMSO-*d*$_6$): δ –70.76 (s). **HRMS** (ESI+): calcd for $C_9H_5F_3N_2O_2$ $[M+H]^+$: 231.0376, found 231.0375.

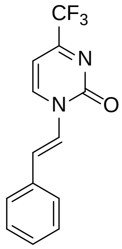

(*E*)-1-Styryl-4-(trifluoromethyl)pyrimidin-2(1*H*)-one (5a). Following the GP1, using compound **1a** (492 mg, 3 mmol, 1 equiv) and (*E*)-styrylboronic acid **4** (444 mg, 3 mmol, 1 equiv). The obtained residue was refluxed in methyl *tert*-butyl ether (20 mL), cooled and filtered off. Yellow solid (694 mg, 87%). Mp 147-148 °C. **IR** (neat): ν_{max} 3099, 3030, 1669, 1528, 1461, 1326, 1204, 1150, 1045, 955, 805, 749. **UV-vis** (CH_2Cl_2): λ_{max} 370 nm. **Fluorescence emission** (CH_2Cl_2): λ_{max} 490 nm. **^{1}H NMR** (400 MHz, DMSO-*d*$_6$): δ 8.86 (d, *J* = 6.6 Hz, 1H), 7.69 (d, *J* = 14.6 Hz, 1H), 7.58 (d, *J* = 7.1 Hz, 2H), 7.49 – 7.33 (m, 3H), 7.30 (d, *J* = 14.7 Hz, 1H), 6.96 (d, *J* = 6.4 Hz, 1H).**^{13}C NMR** (126 MHz, DMSO-*d*$_6$): δ 161.6 (q, *J* = 35.5 Hz), 153.4, 150.5, 134.4, 129.4, 129.3, 127.4, 126.21, 126.1, 120.0 (q, *J* = 277.5 Hz), 100.1. **^{19}F NMR** (376 MHz, DMSO-*d*$_6$): δ –70.36 (s). **HRMS** (ESI+): calcd for $C_{13}H_9F_3N_2O$ $[M+H]^+$: 267.0740, found 267.0741.

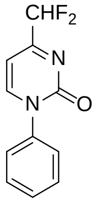

4-(Difluoromethyl)-1-phenylpyrimidin-2(1*H*)-one (9a). Following the GP1, using 4-(difluoromethyl)pyrimidin-2(1H)-one **1b** (438 mg, 3 mmol, 1 equiv) and phenylboronic acid **2a** (336 mg, 3 mmol, 1 equiv). The obtained residue was refluxed in methyl *tert*-butyl ether (20 mL), cooled and filtered off. White solid (266 mg, 40 %). Mp 118-119 °C. **IR** (neat): ν_{max} 3100, 3059, 1667, 1526, 1453, 1079, 1062, 783, 690. **^{1}H NMR** (400 MHz, DMSO-*d*$_6$): δ 8.45 (d, *J* = 6.7 Hz, 1H), 7.59 – 7.47 (m, 5H), 6.77 (t, *J* = 54.1 Hz, 2H), 6.75 (d, *J* = 6.7 Hz, 1H). **^{13}C NMR** (126 MHz, DMSO-*d*$_6$): δ 168.6 (t, *J* = 26.1 Hz), 154.8, 153.4, 140.5, 129.7, 129.4, 126.9, 112.5 (t, *J* = 242.1 Hz), 99.6. **^{19}F NMR** (376 MHz, DMSO-*d*$_6$): δ –121.84 (d, *J* = 54.1 Hz). **HRMS** (ESI+): calcd for $C_{11}H_8F_2N_2O$ [M+H]$^+$: 223.0678, found 223.0677.

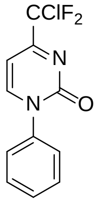

4-(Chlorodifluoromethyl)-1-phenylpyrimidin-2(1*H*)-one (9b). Following the GP1, using 4-(chlorodifluoromethyl)pyrimidin-2(1H)-one **1c** (542 mg, 3 mmol, 1 equiv) and phenylboronic acid **2a** (336 mg, 3 mmol, 1 equiv). The obtained residue was refluxed in methyl *tert*-butyl ether (20 mL), cooled and filtered off. Light yellow solid (596 mg, 74%). Mp 151-152 °C. **IR** (neat): ν_{max} 3058, 2963, 1669, 1526, 1453, 1283, 1148, 1058, 966, 887, 762. **^{1}H NMR** (400 MHz, DMSO-*d*$_6$): δ 8.57 (d, *J* = 6.7 Hz, 1H), 7.66 – 7.47 (m, 5H), 6.90 (d, *J* = 6.7 Hz, 1H). **^{13}C NMR** (126 MHz, DMSO-*d*$_6$): δ 166.4 (t, *J* = 29.9 Hz), 154.9, 154.5, 140.3, 129.7, 129.6, 126.9, 123.0 (t, *J* = 292.8 Hz), 98.6. **^{19}F NMR** (376 MHz, DMSO-*d*$_6$): δ –59.59 (s). **HRMS** (ESI+): calcd for $C_{11}H_7ClF_2N_2O$ [M+H]$^+$: 257.0288, found 257.0287.

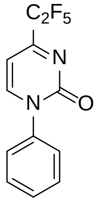

4-(Perfluoroethyl)-1-phenylpyrimidin-2(1*H*)-one (9c). Following the GP1, using 4-(perfluoroethyl)pyrimidin-2(1H)-one **1d** (642 mg, 3 mmol, 1 equiv) and phenylboronic acid **2a** (366 mg, 3 mmol, 1 equiv). The obtained residue was refluxed in methyl *tert*-butyl ether (20 mL), cooled and filtered off. White solid (679 mg, 78%). Mp 154-156 °C. **IR** (neat): ν_{max} 3113, 3062, 1675, 1529, 1453, 1234, 1210, 1174, 1129, 1081, 767, 697. **^{1}H NMR** (400 MHz, DMSO-*d*$_6$): δ 8.61 (d, *J* = 6.7 Hz, 1H), 7.66 – 7.23 (m, 5H), 6.97 (d, *J* = 6.7 Hz, 1H). **^{13}C NMR** (126 MHz, DMSO-*d*$_6$): δ 162.9 (t, *J* = 26.4 Hz), 155.0, 154.1, 140.2, 129.7, 126.9, 120.9 (qt, *J* = 287.2, 36.3 Hz), 110.9 (tq, *J* = 256.8, 37.5 Hz), 100.5. **^{19}F NMR** (376 MHz, DMSO-*d*$_6$): δ –82.18 (s, 3F), –119.04 (s, 2F). **HRMS** (ESI+): calcd for $C_{12}H_7F_5N_2O$ [M+H]$^+$: 291.0552, found 291.0554.

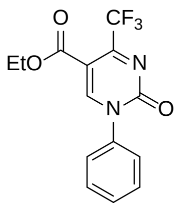

Ethyl 2-oxo-1-phenyl-4-(trifluoromethyl)-1,2-dihydropyrimidine-5-carboxylate (9f). To a mixture of ethyl 2-oxo-4-(trifluoromethyl)-1,2-dihydropyrimidine-5-carboxylate **1g** (708 mg, 3 mmol, 1 equiv), phenylboronic acid **2a** (402 mg, 3.3 mmol, 1.1 equiv) and copper(II) acetate monohydrate (599 mg, 3 mmol, 1 equiv) in dichloromethane (25 mL) pyridine (0.475 g, 0.48 mL, 6 mmol, 2 equiv) was added. The reaction mixture was vigorously stirred at room temperature for 96 h. The solvent was evaporated and the residue was treated with 2 N hydrochloric acid (20 mL) and was extracted with ethyl acetate (2 × 30 mL). The organic layer was washed with brine (2 × 50 mL), dried over anhydrous sodium sulfate and the solvent was evaporated. The obtained residue was refluxed in toluene (25 mL) and acetic acid (0.2 mL) for 5 h, cooled and the precipitate was filtered off. White solid (609 mg, 65 %). Mp 140-142 °C. **IR** (neat): ν_{max} 2993, 1718, 1680, 1623, 1492, 1269, 1206, 1147, 792, 701. **^{1}H NMR** (400 MHz, DMSO-d_6): δ 8.88 (s, 1H), 7.58 (s, 5H), 4.26 (q, *J* = 6.4 Hz, 2H), 1.27 (t, *J* = 6.2 Hz, 3H). **^{13}C NMR** (125 MHz, DMSO-d_6): δ 161.6, 159.4 (q, *J* = 35.6 Hz), 157.6, 153.3, 139.5, 130.0, 129.8, 127.00, 119.7 (q, *J* = 277.9 Hz), 106.3, 62.1, 14.2. **^{19}F NMR** (376 MHz, DMSO-d_6): δ –67.47 (s). **HRMS** (ESI+): calcd for $C_{14}H_{11}F_3N_2O_3$ $[M+H]^+$: 313.0795, found 313.0798.

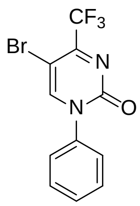

5-Bromo-1-phenyl-4-(trifluoromethyl)pyrimidin-2(1*H*)-one (9g). Following the GP1, using 5-bromo-4-(trifluoromethyl)pyrimidin-2(1*H*)-one **1h** (729 mg, 3 mmol, 1 equiv) and phenylboronic acid **2a** (366 mg, 3 mmol, 1 equiv). The obtained residue was refluxed in methyl *tert*-butyl ether (20 mL), cooled and filtered off. White solid (785 mg, 82 %). Mp 189-192 °C. **IR** (neat): ν_{max} 3058, 1699, 1675, 1487, 1452, 1294, 1216, 1148, 1049, 780, 700. **^{1}H NMR** (400 MHz, DMSO-d_6): δ 8.95 (s, 1H), 7.54 (bs, 5H). **^{13}C NMR** (125 MHz, DMSO-d_6): δ 158.5 (q, *J* = 34.2 Hz), 155.7, 152.8, 139.5, 129.9, 129.7, 127.0, 120.7 (q, *J* = 277.3 Hz), 90.7. **^{19}F NMR** (376 MHz, DMSO-d_6): δ –69.19 (s). **HRMS** (ESI+): calcd for $C_{11}H_6BrF_3N_2O$ $[M+H]^+$: 318.9688, found 318.9686.

3. General procedure 2 (GP2) for the synthesis of compounds 3a,g,q,s, 5a–h and 8 by Chan–Evans–Lam reaction of pyrimidin-2(1*H*)-one 1a with boronic acid pinacol esters 6a–d and 7a–h

To a suspension of compound **1a**, corresponding boronic acid pinacol esters **6b–d** or **7a–h**, copper(II) acetate monohydrate (599 mg, 3 mmol, 1 equiv) and boric acid (371 mg, 6 mmol, 2 equiv) in acetonitrile (20 mL) pyridine (0.475 g, 0.48 mL, 6 mmol, 2 equiv) was added. The mixture was vigorously stirred and heated at 80 °C for 8 h with an open air condenser. After cooling to room temperature the mixture was filtered, the solid material was washed with acetonitrile (2 × 10 mL). The combined filtrates were concentrated under reduced pressure, the residue was treated with 2 N ammonium hydroxide solution (20 mL) and was extracted with ethyl acetate (2 × 30 mL). The organic layers were combined, washed with brine (2 × 30 mL), dried over anhydrous sodium sulfate, filtered and the solvent was evaporated under reduced pressure. The obtained residue was treated as specified below.

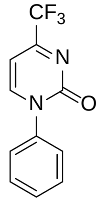

1-Phenyl-4-(trifluoromethyl)pyrimidin-2(1*H*)-one (3a). Following the GP2, using compound **1a** (492 mg, 3 mmol, 1 equiv) and 4,4,5,5-tetramethyl-2-phenyl-1,3,2-dioxaborolane **6a** (933 mg, 4.5 mmol, 1.5 equiv). The obtained residue was refluxed with methyl *tert*-butyl ether (20 mL), cooled and filtered off. White solid (629 mg, 86 %). Physico-chemical and spectral characteristics of this product was identical with the compound obtained according to GP1.

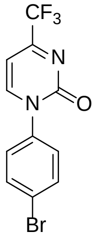

1-(4-Bromophenyl)-4-(trifluoromethyl)pyrimidin-2(1*H*)-one (3g). Following the GP2, using compound **1a** (492 mg, 3 mmol, 1 equiv) and 2-(4-bromophenyl)-4,4,5,5-tetramethyl-1,3,2-dioxaborolane **6b** (1.27 g, 4.5 mmol, 1.5 equiv). The obtained residue was refluxed in methyl *tert*-butyl ether (20 mL), cooled and filtered off. White solid (680 mg, 71%). Physico-chemical and spectral characteristics of this product was identical with the compound obtained according to GP1.

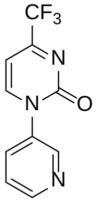

1-(Pyridin-3-yl)-4-(trifluoromethyl)pyrimidin-2(1*H*)-one (3q). Following the GP2, using compound **1a** (492 mg, 3 mmol, 1 equiv) and 3-(4,4,5,5-tetramethyl-1,3,2-dioxaborolan-2-yl)pyridine **6c** (937 mg, 4.5 mmol, 1.5 equiv). The obtained residue was recrystallized from toluene. White solid (109 mg, 15%). Physico-chemical and spectral characteristics of this product was identical with the compound obtained according to GP1.

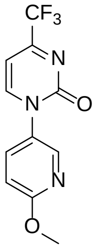

1-(6-Methoxypyridin-3-yl)-4-(trifluoromethyl)pyrimidin-2(1*H*)-one (3s). Following the GP2, using compound **1a** (492 mg, 3 mmol, 1 equiv) and 2-methoxy-5-(4,4,5,5-tetramethyl-1,3,2-dioxaborolan-2-yl)pyridine **6d** (860 mg, 4.5 mmol, 1.5 equiv). The obtained residue was recrystallized from toluene. White solid (372 mg, 45 %). Physico-chemical and spectral characteristics of this product was identical with the compound obtained according to GP1.

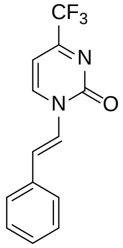

(*E*)-1-Styryl-4-(trifluoromethyl)pyrimidin-2(1*H*)-one (5a). Following the GP2, using compound **1a** (492 mg, 3 mmol, 1 equiv) and (*E*)-4,4,5,5-tetramethyl-2-styryl-1,3,2-dioxaborolane **7a** (1035 mg, 4.5 mmol, 1.5 equiv). The obtained residue was refluxed with methyl *tert*-butyl ether (20 mL), cooled and filtered off. White solid (654 mg, 82 %). Physico-chemical and spectral characteristics of this product was identical with the compound obtained according to GP1.

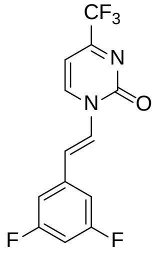

(*E*)-1-(3,5-Difluorostyryl)-4-(trifluoromethyl)pyrimidin-2(1*H*)-one (5b). Following the GP2, using compound **1a** (492 mg, 3 mmol, 1 equiv) and (*E*)-2-(3,5-difluorostyryl)-4,4,5,5-tetramethyl-1,3,2-dioxaborolane **7b** (1.197 g, 4.5 mmol, 1.5 equiv). The obtained residue was refluxed in methyl *tert*-butyl ether (20 mL), cooled and filtered off. Yellow solid (680 mg, 75%). Mp 236-237 °C. **IR** (neat): ν_{max} 3098, 3033, 1667, 1528, 1459, 1309, 1209, 1161, 940, 810, 671. **UV-vis** (CH_2Cl_2): λ_{max} 369 nm. **Fluorescence emission** (CH_2Cl_2): λ_{max} 491 nm. **^{1}H NMR** (400 MHz, DMSO-d_6): δ 8.81 (d, *J* = 6.8 Hz, 1H), 7.85 (d, *J* = 14.6 Hz, 1H), 7.38 (d, *J* = 6.8 Hz, 2H), 7.32 (d, *J* = 14.6 Hz, 1H), 7.23 (t, *J* = 9.2 Hz, 1H), 7.03 (d, *J* = 6.9 Hz, 1H). **^{13}C NMR** (126 MHz,

DMSO-d_6): δ 163.1 (dd, *J* = 246.0, 13.6 Hz),162.0 (q, *J* = 35.6 Hz), 153.3, 150.6, 138.4 (t, *J* = 10.2 Hz), 128.9, 124.1, 119.9 (q, *J* = 277.5 Hz), 110.5 (dd, *J* = 20.0, 6.2 Hz), 104.3 (t, *J* = 26.1 Hz), 100.2. **^{19}F NMR** (376 MHz, DMSO-d_6): δ –70.85 (s, 3F), –109.97 (s, 2F). **HRMS** (ESI+): calcd for $C_{13}H_7F_5N_2O$ $[M+H]^+$: 303.0552, found 303.0550.

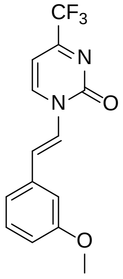

(*E*)-1-(3-Methoxystyryl)-4-(trifluoromethyl)pyrimidin-2(1*H*)-one (5c). Following the GP2, using compound **1a** (492 mg, 3 mmol, 1 equiv) and (*E*)-2-(3-methoxystyryl)-4,4,5,5-tetramethyl-1,3,2-dioxaborolane **7c** (1.17 g, 4.5 mmol, 1.5 equiv). The reaction was carried out in darkness. The obtained residue was refluxed in methyl *tert*-butyl ether (20 mL), cooled and filtered off. Yellow solid (498 mg, 56%). Mp 187-188 °C. **IR** (neat): $ν_{max}$ 3060. **UV-vis** (CH_2Cl_2): $λ_{max}$ 372 nm. **Fluorescence emission** (CH_2Cl_2): $λ_{max}$ 493 nm. **^{1}H NMR** (400 MHz, DMSO-d_6): δ 8.86 (d, *J* = 6.7 Hz, 1H), 7.72 (d, *J* = 14.6 Hz, 1H), 7.33 (t, *J* = 7.7 Hz, 1H), 7.27 (d, *J* = 14.6 Hz, 1H), 7.15 (d, *J* = 7.5 Hz, 2H), 7.00 (d, *J* = 6.7 Hz, 1H), 6.93 (d, *J* = 7.6 Hz, 1H).**^{13}C NMR** (126 MHz, DMSO-d_6): δ 161.2 (q, *J* = 35.7 Hz), 159.6, 153.0, 150.1, 135.4, 130.0, 126.0, 125.8, 119.6 (d, *J* = 277.3 Hz), 119.5, 114.7, 112.0, 99.7, 55.2. **^{19}F NMR** (376 MHz, DMSO-d_6): δ –70.35 (s). **HRMS** (ESI+): calcd for $C_{14}H_{11}F_3N_2O_2$ $[M+H]^+$: 297.0844, found 297.0846.

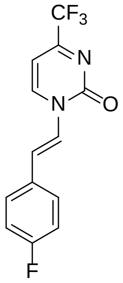

(*E*)-1-(4-Fluorostyryl)-4-(trifluoromethyl)pyrimidin-2(1*H*)-one (5d). Following the GP2, using compound **1a** (492 mg, 3 mmol, 1 equiv) and (*E*)-2-(4-fluorostyryl)-4,4,5,5-tetramethyl-1,3,2-dioxaborolane **7d** (1.116 g, 4.5 mmol, 1.5 equiv). The obtained residue was refluxed in methyl *tret*-butyl ether, cooled and filtered off. Yellow-green solid (780 mg, 91.5%). Mp 235-236 °C. **IR** (neat): $ν_{max}$ 3110, 3029, 1659, 1512, 1458, 1322, 1206, 1170, 1043, 956, 817. **UV-vis** (CH_2Cl_2): $λ_{max}$ 368 nm. **Fluorescence emission** (CH_2Cl_2): $λ_{max}$ 492 nm. **^{1}H NMR** (400 MHz, DMSO-d_6): δ 8.85 (d, *J* = 6.8 Hz, 1H), 7.82 – 7.48 (m, 3H), 7.35 – 7.17 (m, 3H), 7.00 (d, *J* = 6.8 Hz, 1H). **^{13}C NMR** (126 MHz, DMSO-d_6): δ 162.77 (d, *J* = 246.5 Hz), 161.68 (q, *J* = 35.3 Hz), 153.5, 150.6, 131.0 (d, *J* = 3.2 Hz), 129.6 (d, *J* = 8.3 Hz), 126.1, 125.2, 120.0 (q, *J* = 277.2 Hz), 116.4 (d, *J* = 21.8 Hz), 100.1. **^{19}F NMR** (376 MHz, DMSO-d_6): δ –70.77 (s, 3F), –112.89 (s, 1F). **HRMS** (ESI+): calcd for $C_{13}H_8F_4N_2O$ $[M+H]^+$: 285.0646, found 285.0652.

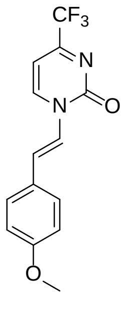

(*E*)-1-(4-Methoxystyryl)-4-(trifluoromethyl)pyrimidin-2(1*H*)-one (5e). Following the GP2, using compound **1a** (492 mg, 3 mmol, 1 equiv) and (*E*)-2-(4-methoxystyryl)-4,4,5,5-tetramethyl-1,3,2-dioxaborolane **7e** (1.17 g, 4.5 mmol, 1.5 equiv). The obtained residue was refluxed in methyl *tert*-butyl ether (20 mL), cooled and filtered off. Yellow-green solid (542 mg, 61%). Mp 234-236 °C. **IR** (neat): ν_{max} 3098, 3036, 2961, 2938, 1666, 1528, 1459, 1328, 1257, 1206, 1146, 814. **UV-vis** (CH_2Cl_2): λ_{max} 383 nm. **Fluorescence emission** (CH_2Cl_2): λ_{max} 532 nm. **^{1}H NMR** (400 MHz, DMSO-d_6): δ 8.54 (d, *J* = 6.6 Hz, 1H), 7.18 (s, 1H), 7.06 (d, *J* = 8.2 Hz, 1H), 7.00 (d, *J* = 8.3 Hz, 1H), 6.92 (d, *J* = 6.7 Hz, 1H), 6.13 (s, 2H). **^{13}C NMR** (126 MHz, DMSO-d_6): δ 161.3 (q, *J* = 35.1 Hz), 160.3, 153.5, 150.5, 129.0, 126.9, 126.1, 124.2, 120.0 (d, *J* = 277.5 Hz), 114.9, 100.0, 55.7. **^{19}F NMR** (376 MHz, DMSO-d_6): δ –70.70 (s). **HRMS** (ESI+): calcd for $C_{14}H_{11}F_3N_2O_2$ [M+H]$^+$: 297.0846, found 297.0846.

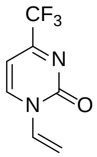

4-(Trifluoromethyl)-1-vinylpyrimidin-2(1*H*)-one (5f). Following the GP2, using compound **1a** (492 mg, 3 mmol, 1 equiv) and vinylboronic acid pinacol ester **7f** (939 mg, 6.0 mmol, 2 equiv). The obtained residue was washed with water (2×10 mL), dried and recrystallized from toluene. White solid (336 mg, 58 %). Mp 136-138 °C. **IR** (neat): ν_{max} 3037, 1685, 1623, 1526, 1470, 1336, 1200, 1145, 1113, 958, 814, 790. **^{1}H NMR** (400 MHz, DMSO-d_6): δ 8.77 (d, *J* = 6.4 Hz, 1H), 7.22 (dd, *J* = 15.7, 8.9 Hz, 1H), 6.94 (d, *J* = 6.4 Hz, 1H), 5.84 (d, *J* = 15.8 Hz, 1H), 5.39 (d, *J* = 8.6 Hz, 1H). **^{13}C NMR** (125 MHz, DMSO-d_6): δ 162.1 (q, *J* = 35.5 Hz), 153.2, 150.1, 132.7, 119.9 (q, *J* = 277.5 Hz), 110.3, 100.0. **^{19}F NMR** (376 MHz, DMSO-d_6): δ –71.04 (s). **HRMS** (ESI+): calcd for $C_7H_5F_3N_2O$ [M+H]$^+$: 191.0427, found 191.0429.

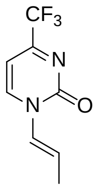

(*E*)-1-(Prop-1-en-1-yl)-4-(trifluoromethyl)pyrimidin-2(1*H*)-one (5g). Following the GP2, using compound **1a** (492 mg, 3 mmol, 1 equiv) and propen-1-ylboronic acid pinacol ester **7g** (1.024 g, 6.0 mmol, 2 equiv). The obtained residue was washed with water (2×10 mL), dried and recrystallized from toluene. White solid (367 mg, 60 %). Mp 202-204 °C. **IR** (neat): ν_{max} 3030, 1682, 1530, 1467, 1332, 1206, 1147, 1110, 952, 812,

794. ^{1}**H NMR** (400 MHz, DMSO-d_6): δ 8.67 (d, J = 6.5 Hz, 1H), 6.95 (d, J = 14.2 Hz, 1H), 6.90 (d, J = 6.5 Hz, 1H), 6.21 – 6.30 (m, 1H), 1.83 (d, J = 6.6 Hz, 1H). ^{13}**C NMR** (150 MHz, DMSO-d_6): δ 161.5 (q, J = 35.4 Hz), 153.3, 151.1, 127.4, 123.7, 119.9 (q, J = 277.5 Hz), 99.7, 15.5. ^{19}**F NMR** (376 MHz, DMSO-d_6): δ –70.91 (s). **HRMS** (ESI+): calcd for $C_8H_7F_3N_2O$ [M+H]$^+$: 205.0583, found 205.0587.

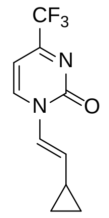

(*E*)-1-(2-Cyclopropylvinyl)-4-(trifluoromethyl)pyrimidin-2(1*H*)-one (5h). Following the GP2, using compound **1a** (492 mg, 3 mmol, 1 equiv) and 2-cyclopropylvinyboronic acid pinacol esther **7h** (1.164 g, 6.0 mmol, 2 equiv). The obtained residue was washed with water (2 × 10 mL), dried and recrystallized from toluene. White solid (428 mg, 62 %). Mp 150-152 °C. **IR** (neat): v_{max} 3095, 2083, 1664, 1534, 1460, 1331, 1303, 1201, 1150, 1096, 1042, 945, 808. ^{1}**H NMR** (400 MHz, DMSO-d_6): δ 8.64 (d, J = 6.7 Hz, 1H), 7.04 (d, J = 14.0 Hz, 1H), 6.88 (d, J = 6.7 Hz, 1H), 5.83 (dd, J = 14.0, 9.6 Hz, 1H), 1.75 – 1.60 (m, 1H), 0.90 – 0.75 (m, 2H), 0.60 – 0.45 (m, 2H). ^{13}**C NMR** (150 MHz, DMSO-d_6): δ 161.2 (q, J = 35.3 Hz), 153.2, 150.7, 133.0, 124.3, 119.9 (q, J = 277.4 Hz), 99.7, 12.4, 7.5. ^{19}**F NMR** (376 MHz, DMSO-d_6): δ –70.96 (s). **HRMS** (ESI+): calcd for $C_{10}H_9F_3N_2O$ [M+H]$^+$: 231.0740, found 231.0742.

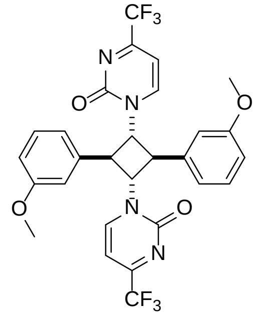

1,1'-(2,4-Bis(3-methoxyphenyl)cyclobutane-1,3-diyl)bis(4-(trifluoromethyl)pyrimidin-2(1*H*)-one) (8). Compound **5c** (100 mg) was exposed to sunlight in an open air Petri dish at room temperature for 12 h. The solid was washed with ethyl acetate (2×20 mL) and air-dried. White solid (76 mg, 76%). Mp >260 °C. **IR** (neat): v_{max} 3099, 3024, 2944, 1662, 1588, 1531, 1466, 1317, 1205, 1142, 1051, 792. ^{1}**H NMR** (400 MHz, DMSO-d_6): δ 8.65 (d, J = 6.7 Hz, 2H), 7.19 (t, J = 8.1 Hz, 2H), 6.91 – 6.61 (m, 8H), 6.06 – 5.94 (m, 2H), 5.20 – 4.91 (m, 2H), 3.68 (s, 6H). ^{13}**C NMR** (126 MHz, DMSO-d_6): δ 161.1 (q, J = 35.6 Hz), 159.7, 154.4, 152.6, 136.9, 130.0, 120.4, 119.9 (q, J = 277.3 Hz), 114.1, 113.4, 99.0, 58.7, 55.5, 45.2. ^{19}**F NMR** (376 MHz, DMSO-d_6): δ –70.89 (s). **HRMS** (ESI+): calcd for $C_{28}H_{22}F_6N_4O_4$ [M+H]$^+$: 593.1618, found 593.1617.

4. References

1. Gorbunova, M. G.; Gerus, I. I.; Kukhar, V. P. Synthesis and Properties of β-Ethoxyvinyl Polyfluoroalkyl Ketones. *Synthesis (Stuttg).* **2000**, *2000* (05), 738–742. https://doi.org/10.1055/s-2000-6386.

5. Copies of the ^{1}H and ^{13}C NMR spectra

1-Phenyl-4-(trifluoromethyl)pyrimidin-2(*1H*)-one (3a)

^{1}H NMR (400 MHz, DMSO-*d*$_6$):

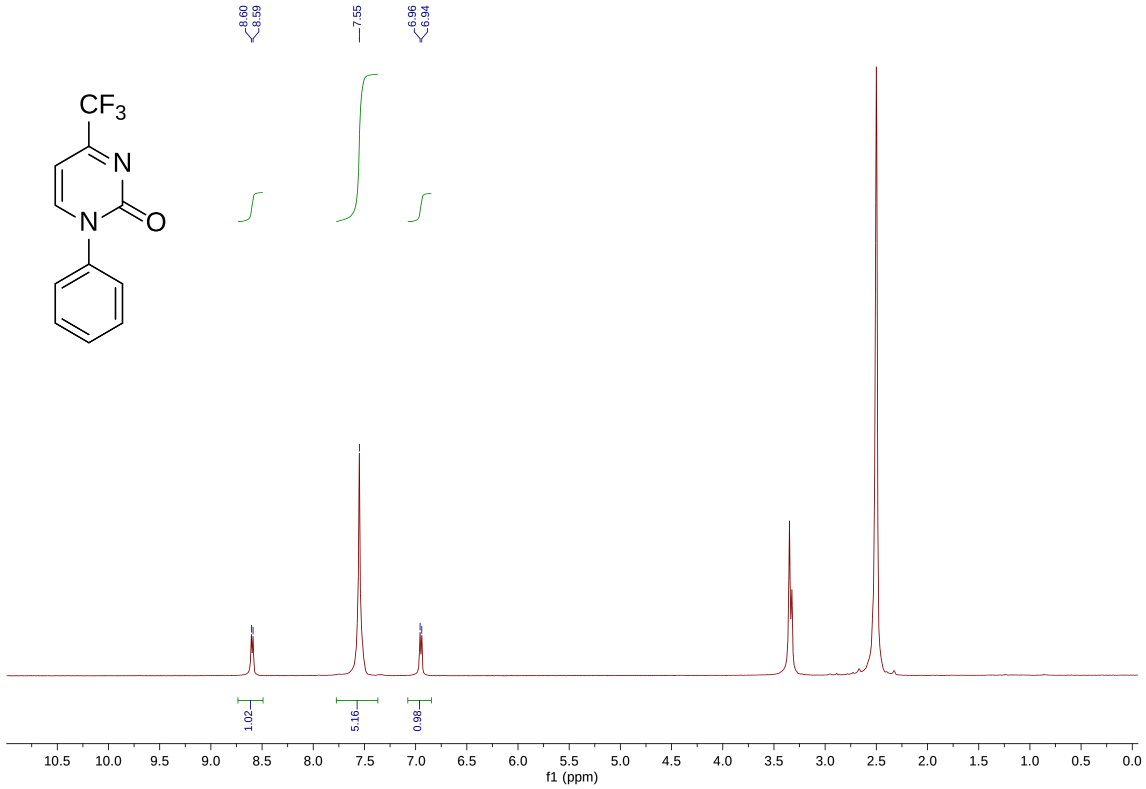

^{13}C NMR (125 MHz, DMSO-*d*$_6$):

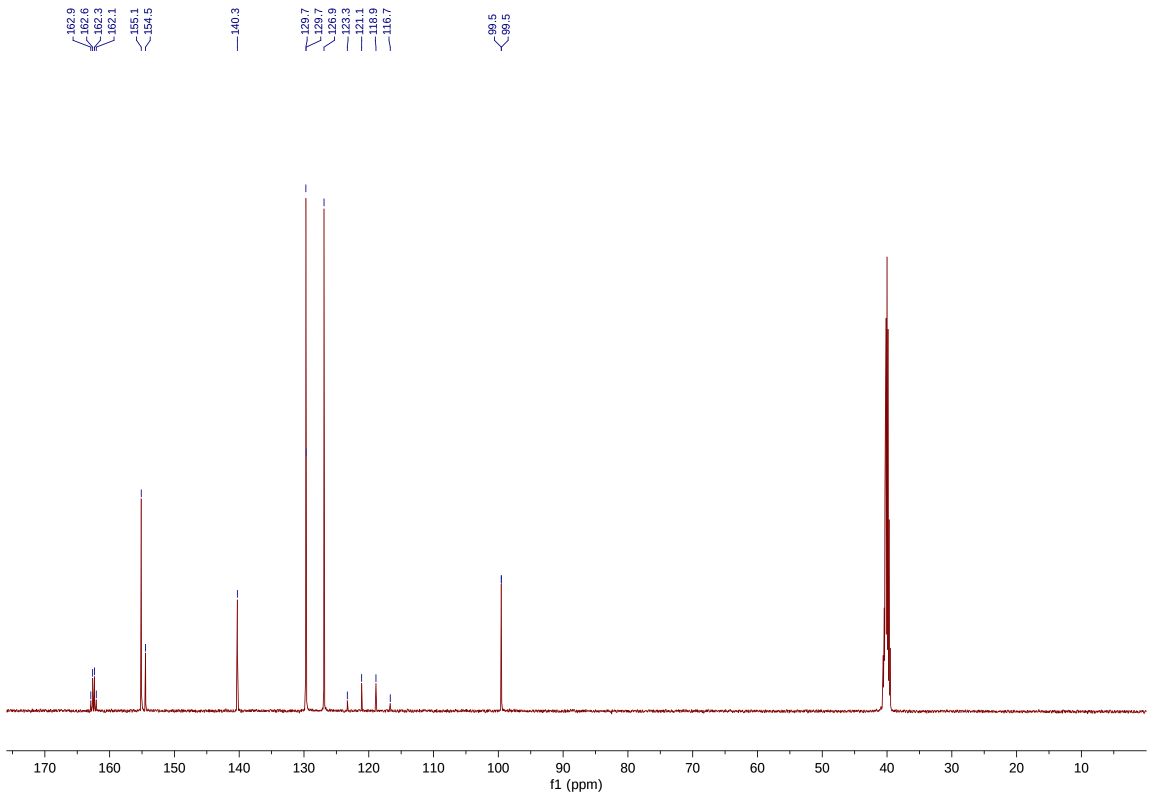

1-(4-Methoxyphenyl)-4-(trifluoromethyl)pyrimidin-2(1*H*)-one (3b)

^{1}H NMR (400 MHz, DMSO-d_6):

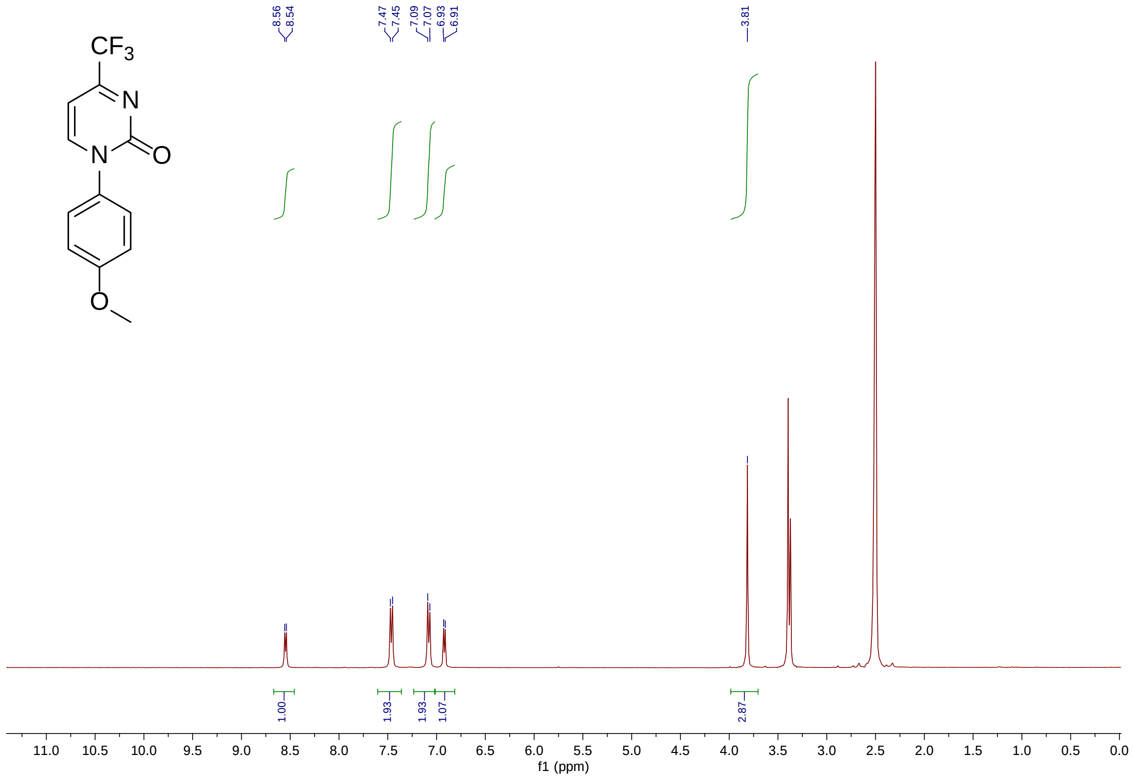

^{13}C NMR (125 MHz, DMSO-d_6):

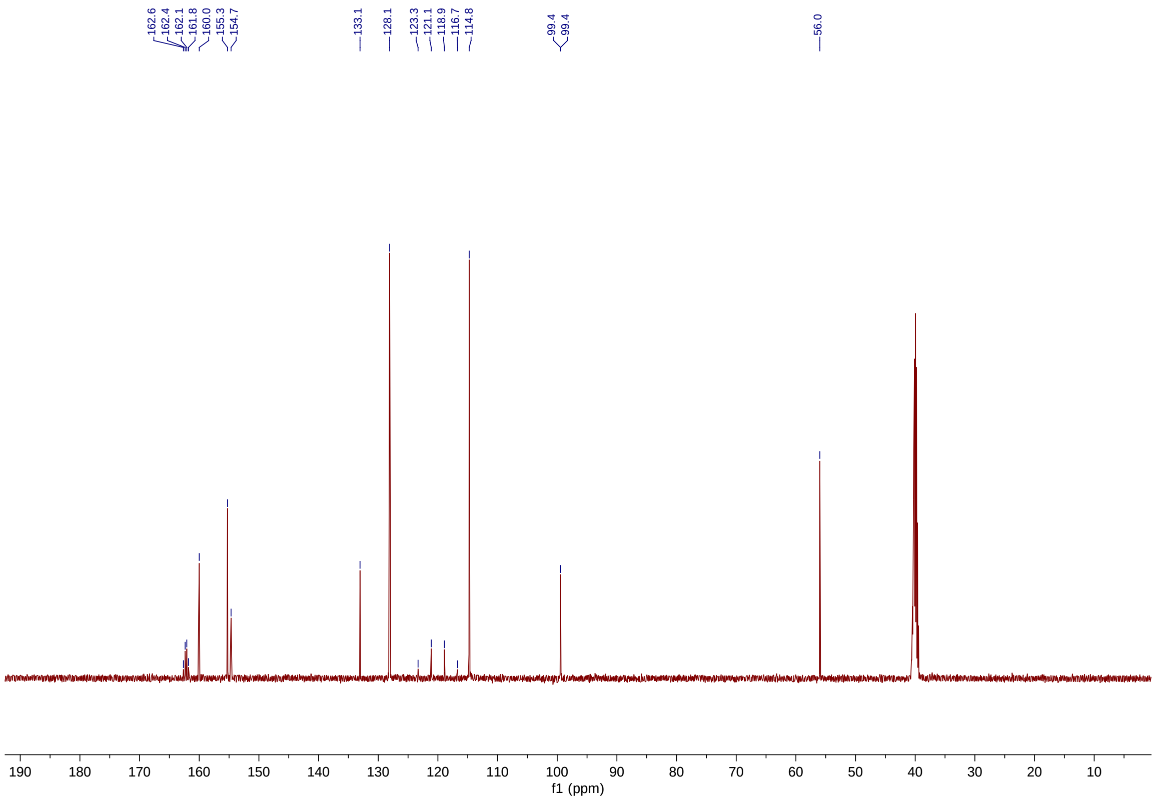

NOE experiment

^{1}H NMR (600 MHz, DMSO-d_6):

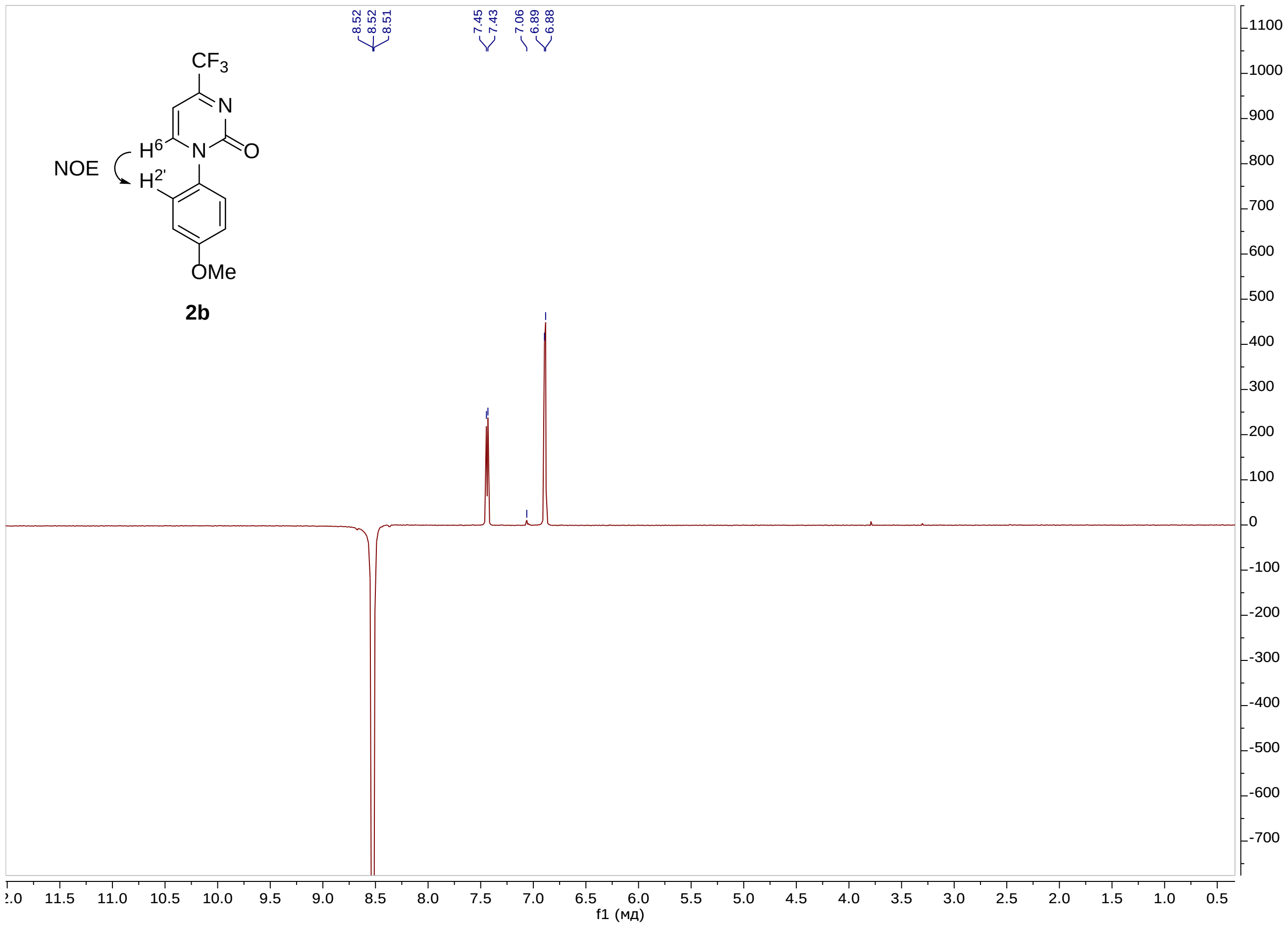
8.52
8.52
8.51
7.45
7.43
7.06
6.89
6.88
CF3
N
O
H6
N
NOE
H2'
OMe
2b
f1 (мд)

1-(Benzo[*d*][1,3]dioxol-5-yl)-4-(trifluoromethyl)pyrimidin-2(1*H*)-one (3c)

^{1}H NMR (400 MHz, DMSO-d_6):

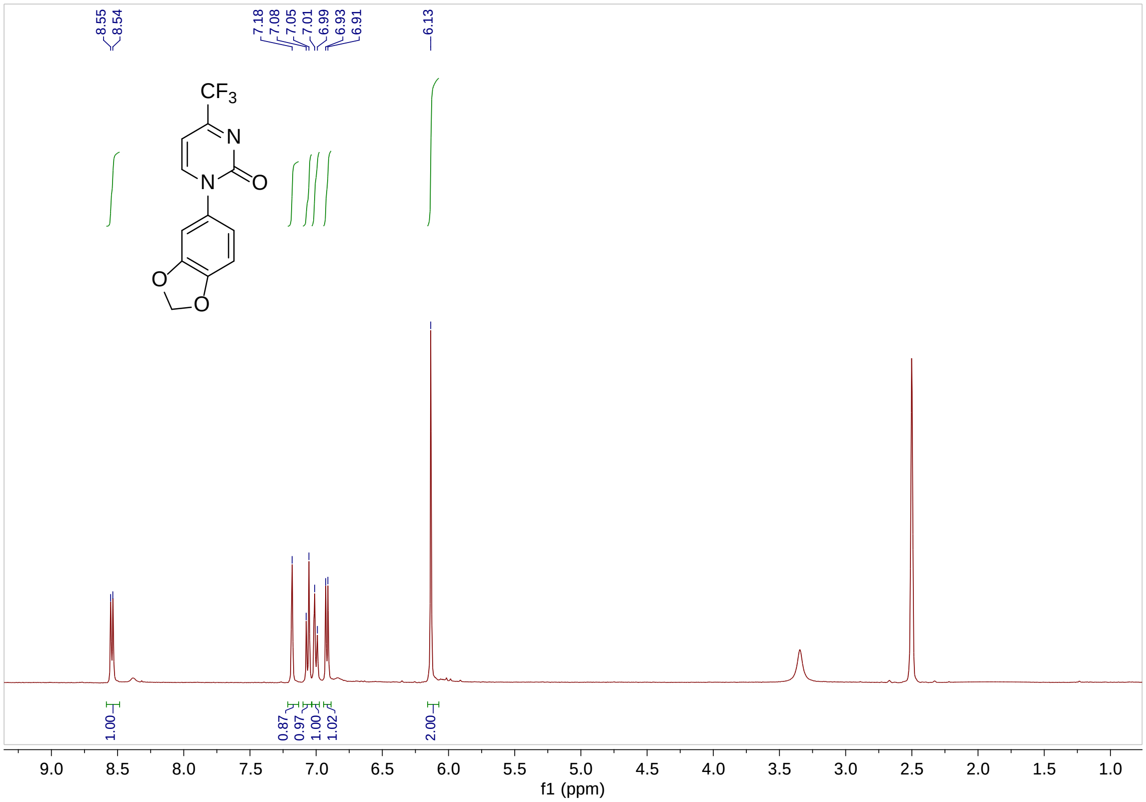

^{13}C NMR (125 MHz, DMSO-d_6):

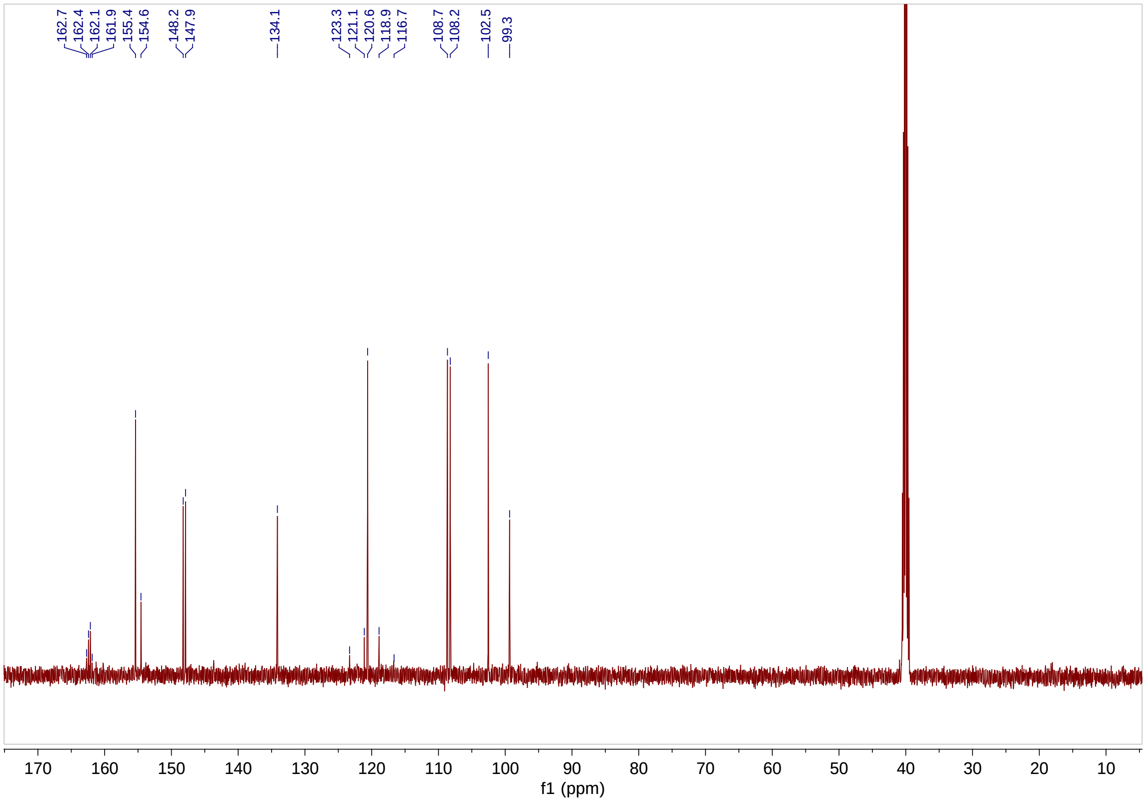

1-(4-(Trifluoromethoxy)phenyl)-4-(trifluoromethyl)pyrimidin-2(1*H*)-one (3d)

^{1}H NMR (400 MHz, DMSO-d_6):

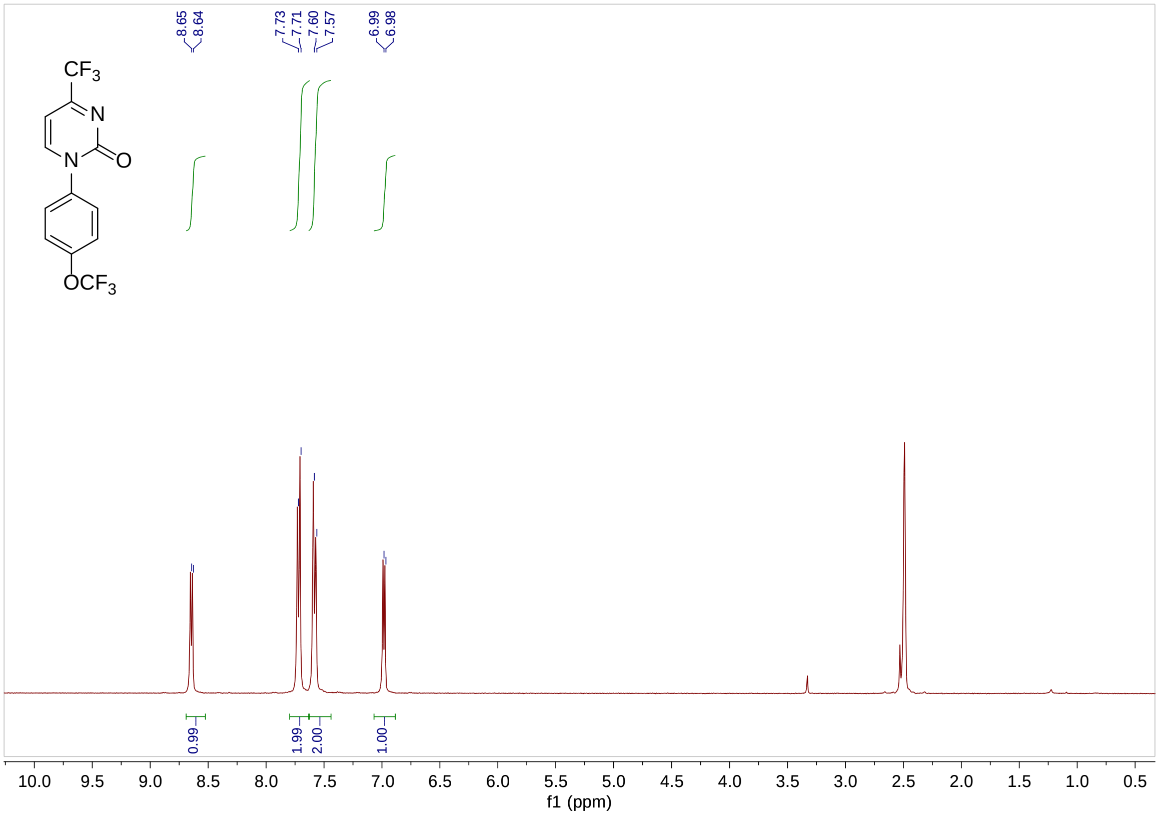

^{13}C NMR (125 MHz, DMSO-d_6):

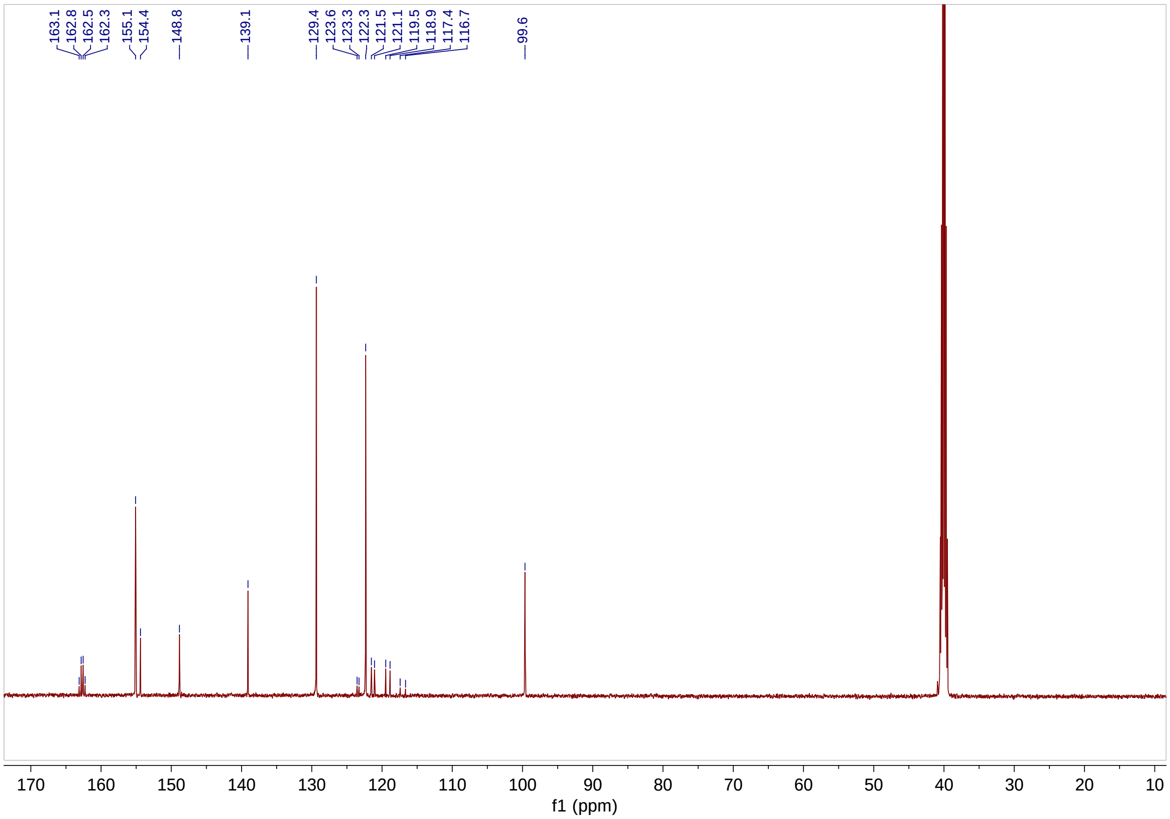

1-[4-(1*H*-Pyrazol-1-yl)phenyl]-4-(trifluoromethyl)pyrimidin-2(1*H*)-on (3e)

^{1}H NMR (400 MHz, DMSO-d_6):

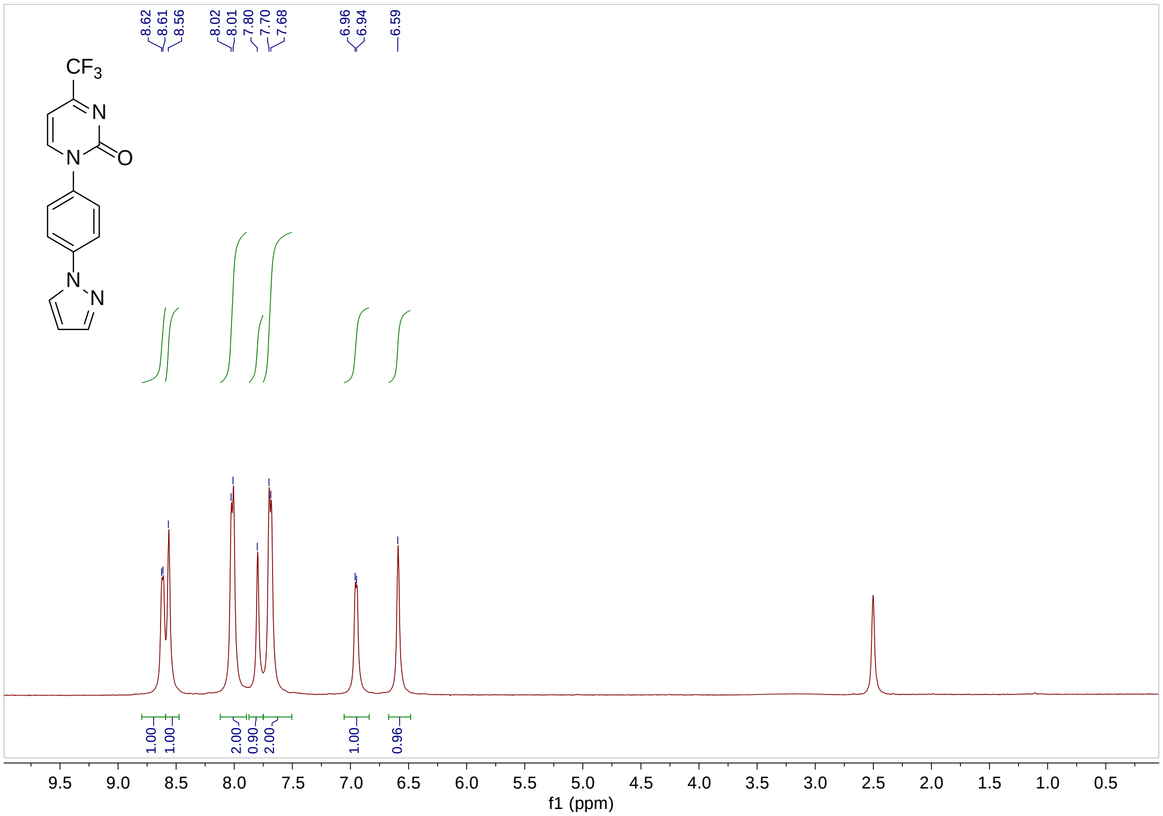

^{13}C NMR (125 MHz, DMSO-d_6):

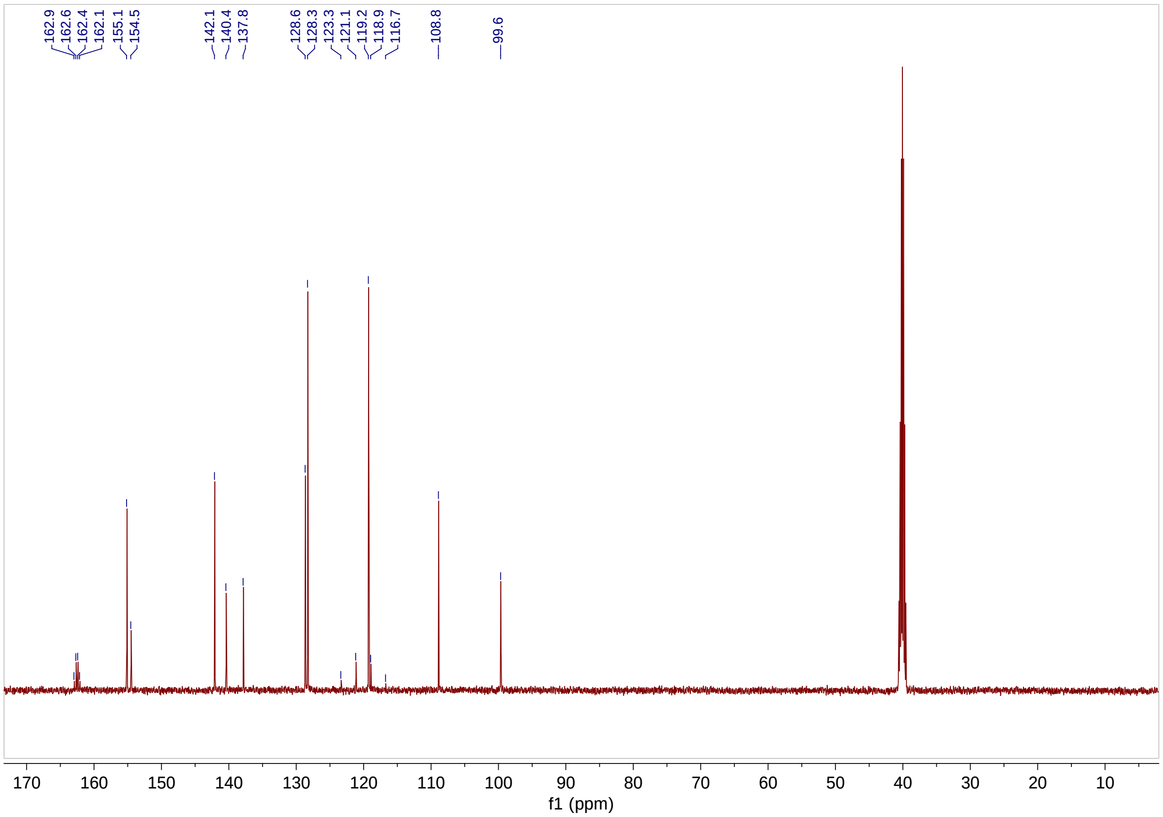

1-(4-((1*H*-1,2,4-Triazol-1-yl)methyl)phenyl)-4-(trifluoromethyl)pyrimidin-2(1*H*)-one (3f)

^{1}H NMR (400 MHz, DMSO-d_6):

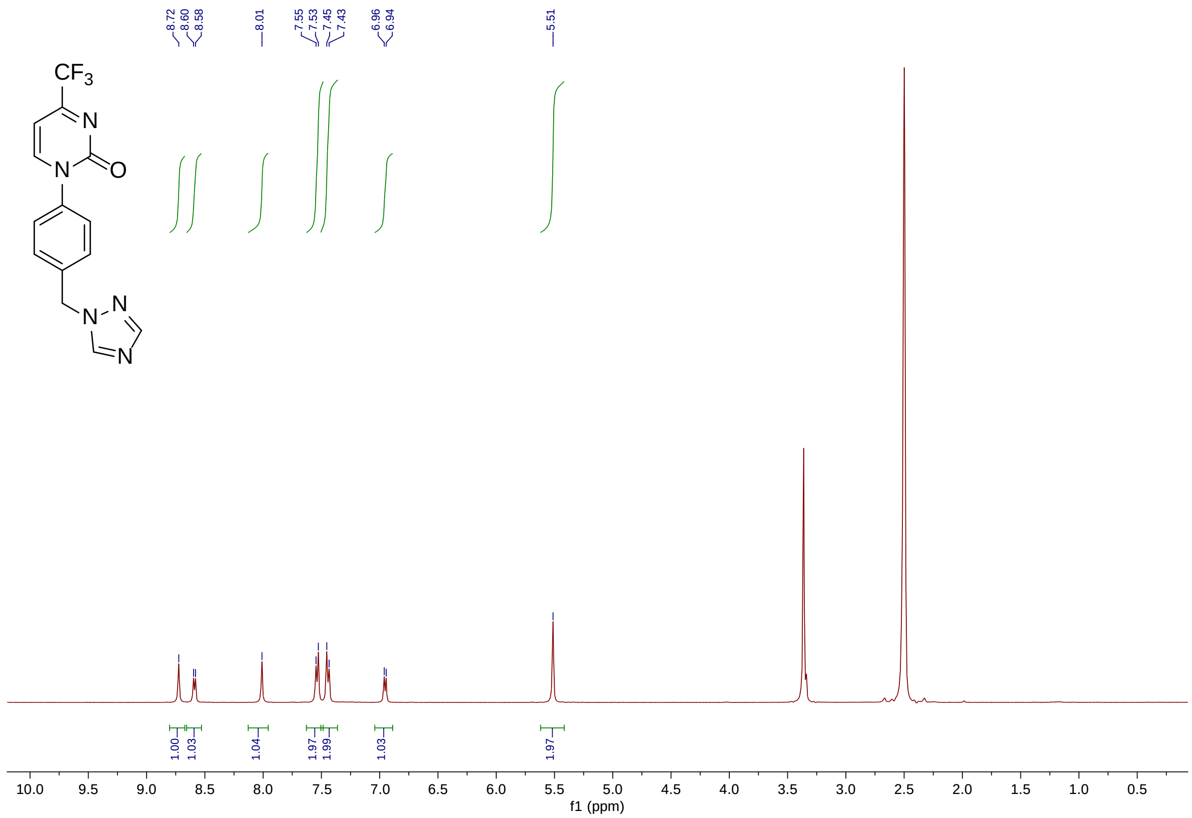

^{13}C NMR (150 MHz, DMSO-d_6):

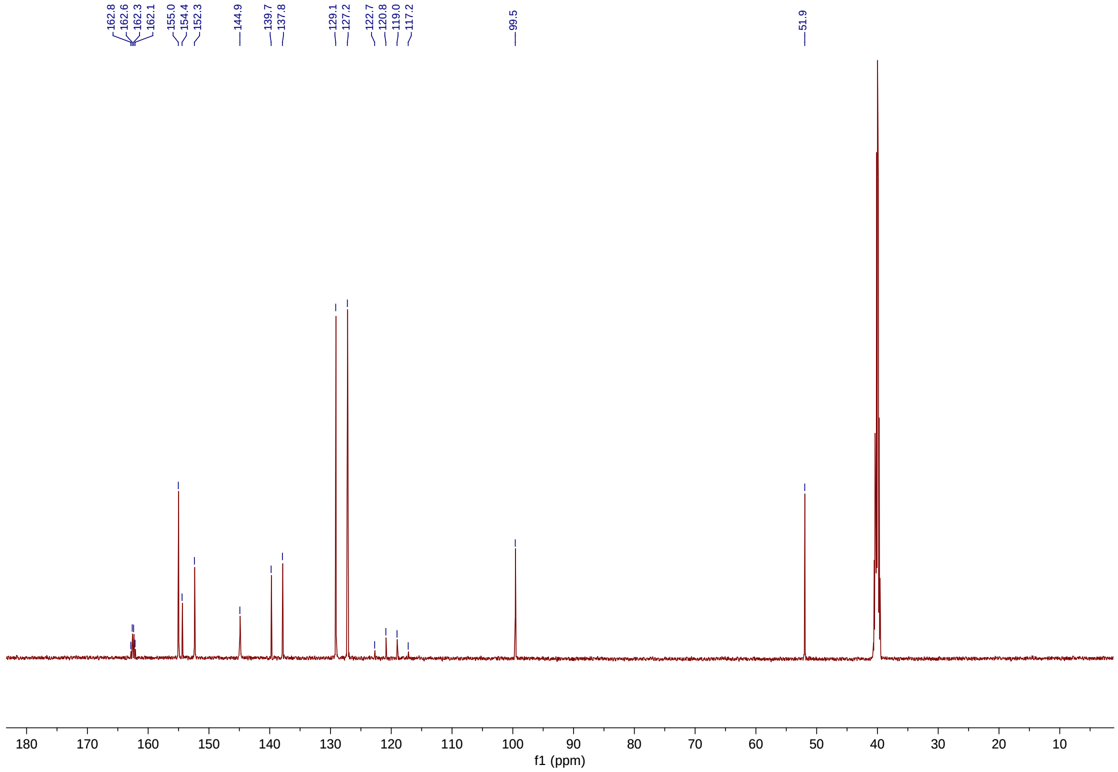

1-(4-Bromophenyl)-4-(trifluoromethyl)pyrimidin-2(1*H*)-one (3g)

^{1}H NMR (400 MHz, DMSO-d_6):

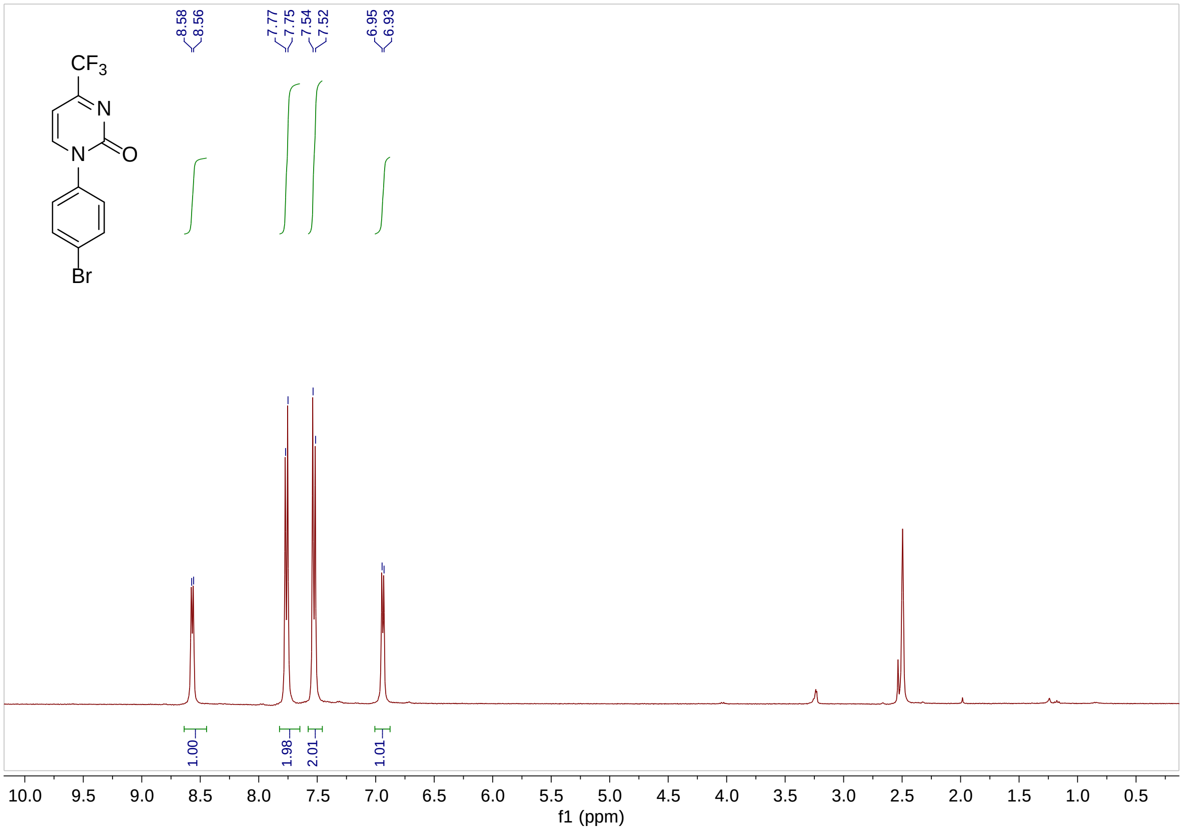

^{13}C NMR (125 MHz, DMSO-d_6):

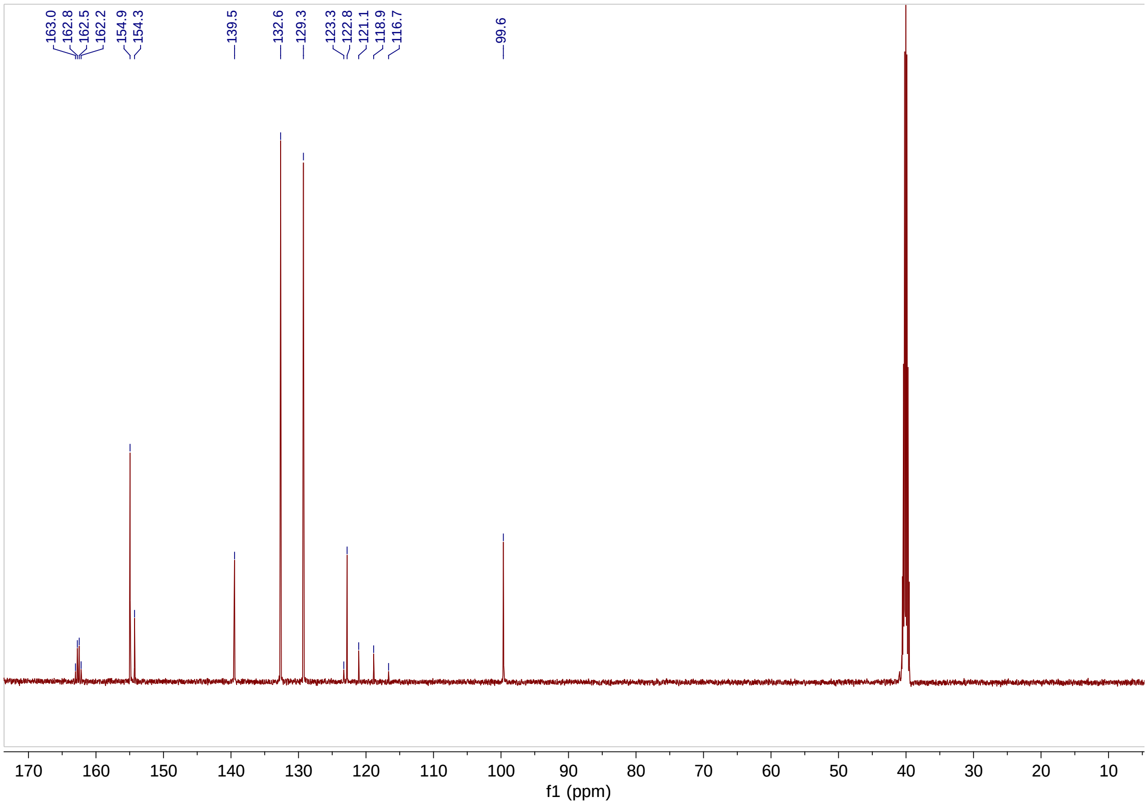

4-(Trifluoromethyl)-1-(4-(trifluoromethyl)phenyl)pyrimidin-2(1*H*)-one (3h)

^{1}H NMR (400 MHz, DMSO-d_6):

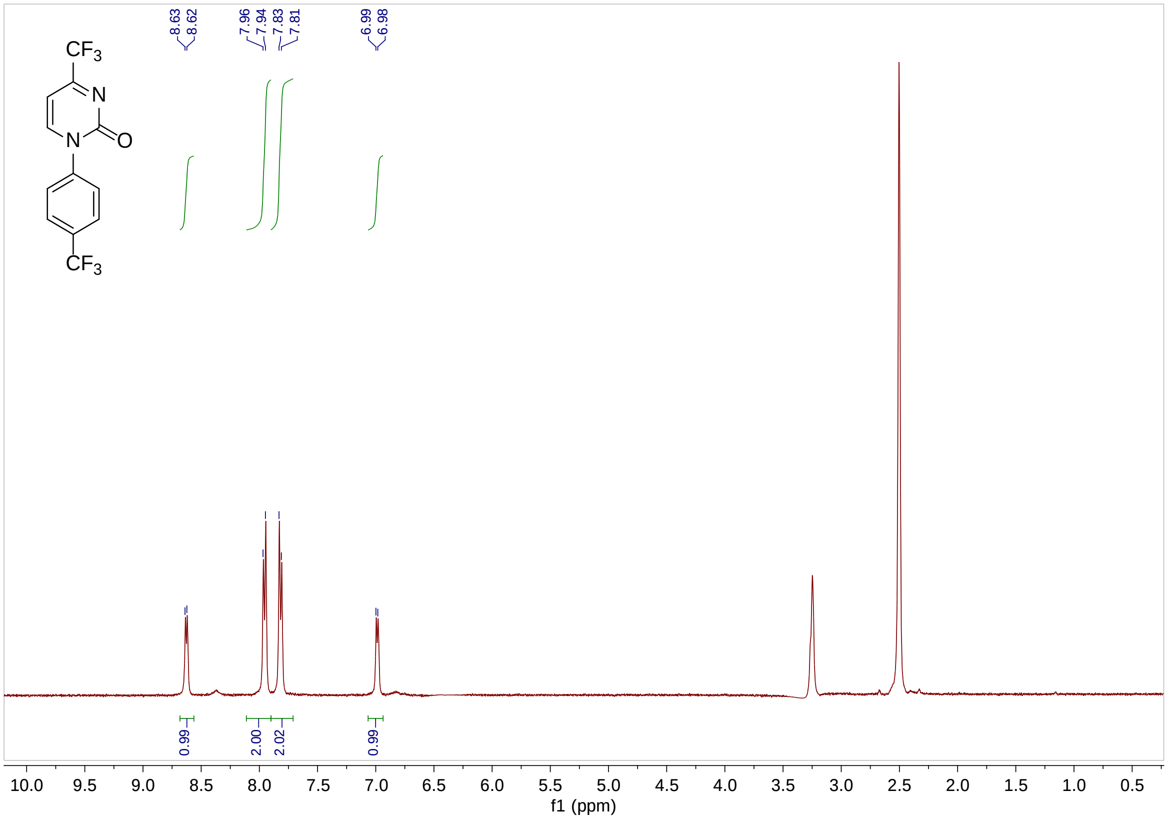

^{13}C NMR (125 MHz, DMSO-d_6):

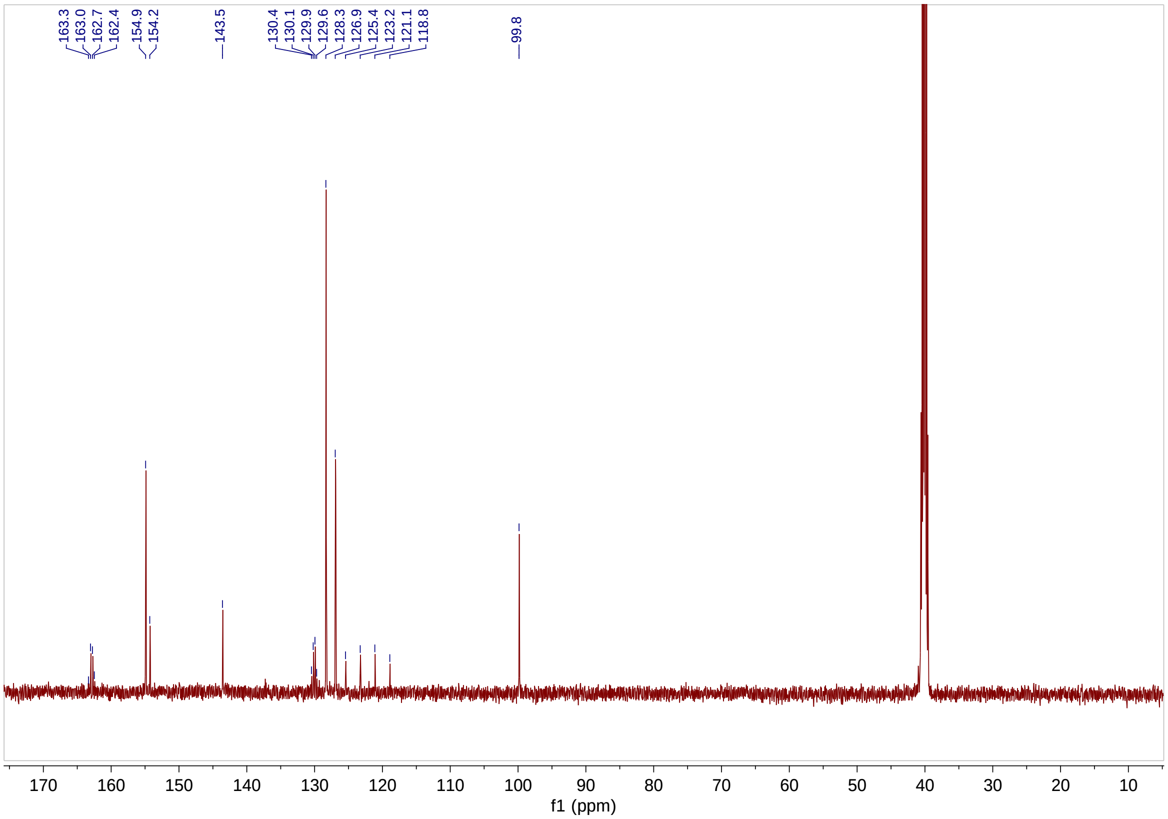

4-(2-Oxo-4-(trifluoromethyl)pyrimidin-1(2*H*)-yl)benzamide (3i)

^{1}H NMR (400 MHz, DMSO-d_6):

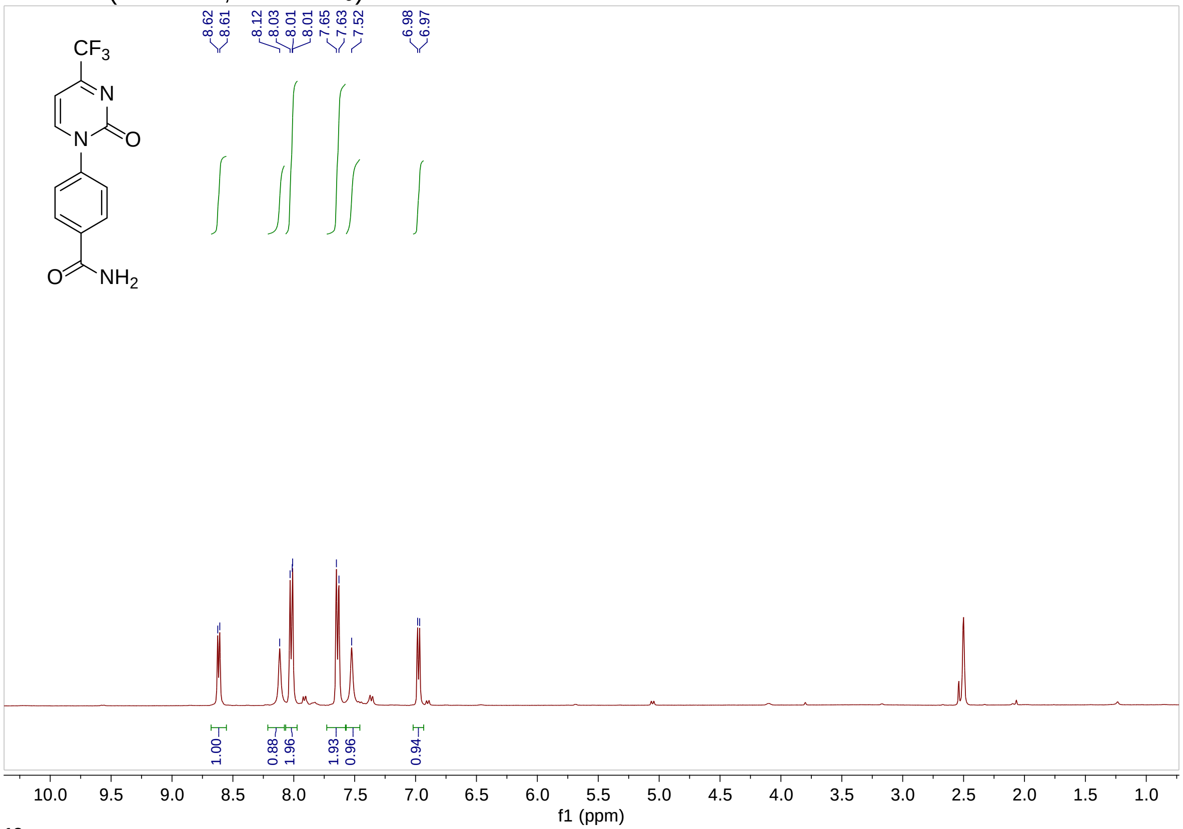

^{13}C NMR (125 MHz, DMSO-d_6):

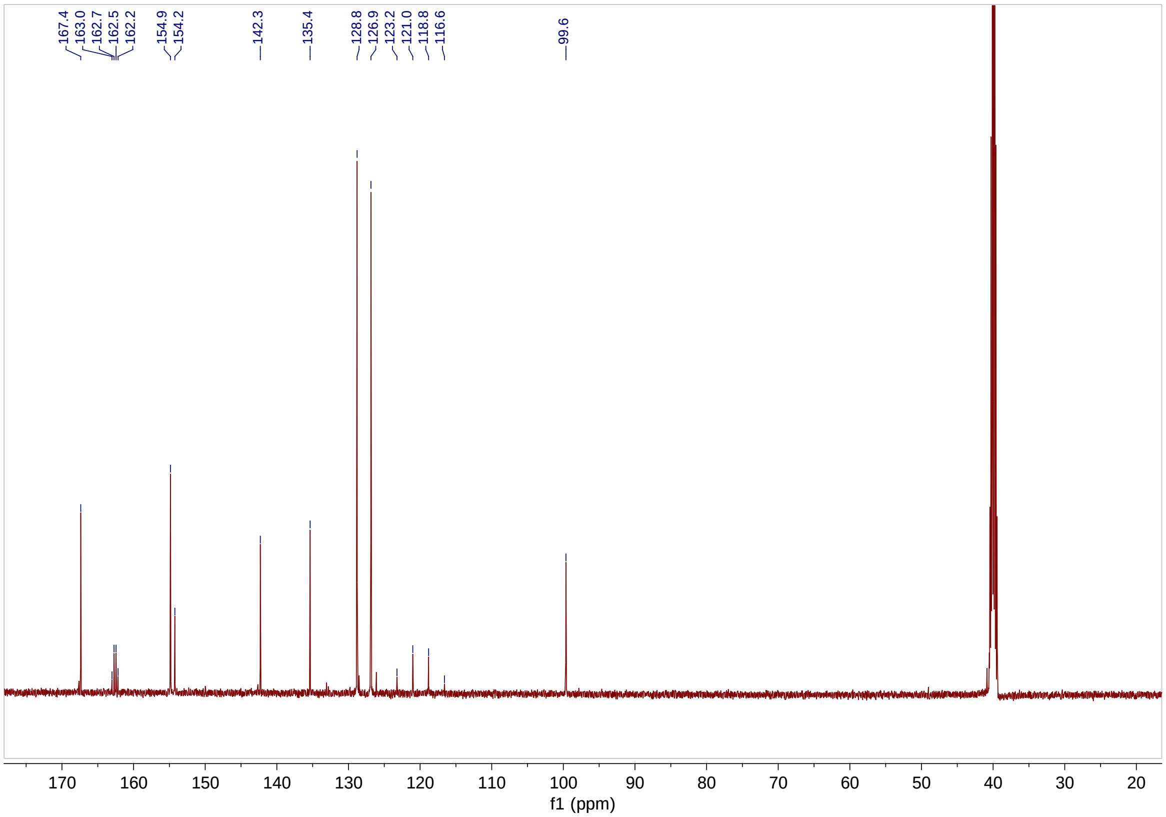

1-(4-(Methylsulfonyl)phenyl)-4-(trifluoromethyl)pyrimidin-2(1*H*)-one (3j)

^{1}H NMR (400 MHz, DMSO-d_6):

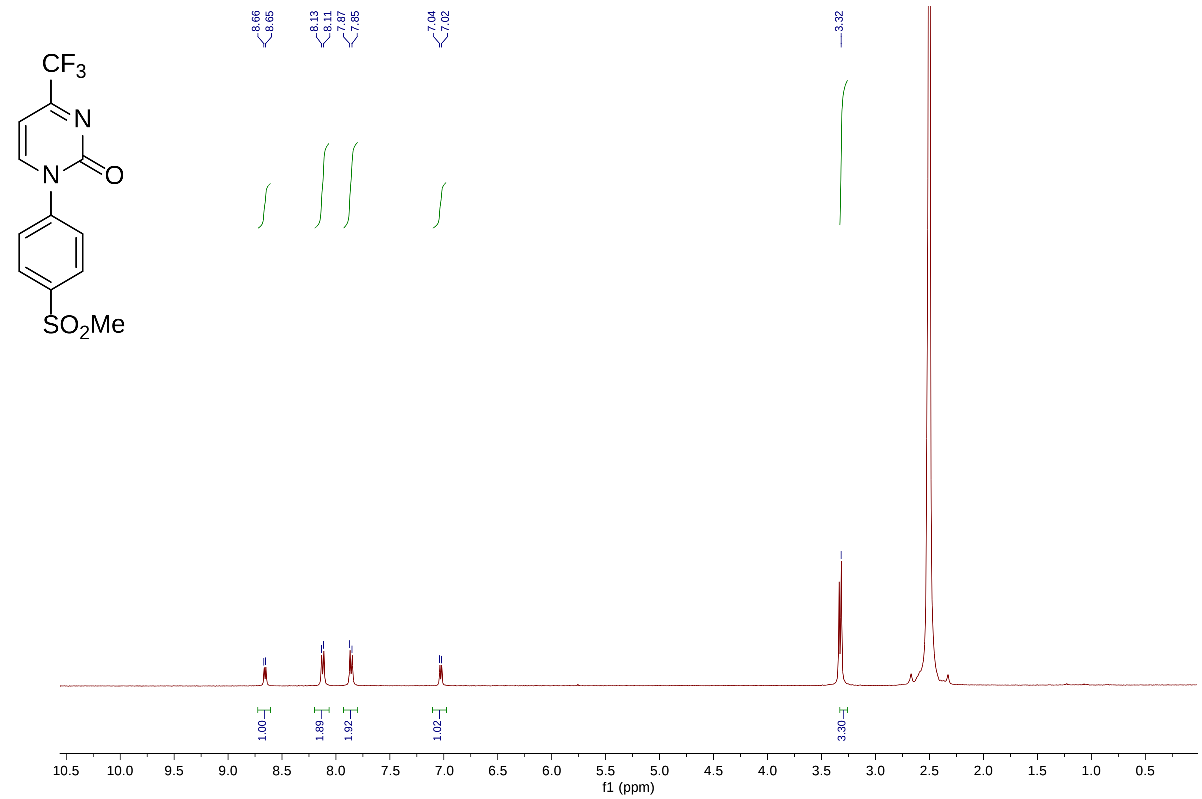

^{13}C NMR (125 MHz, DMSO-d_6):

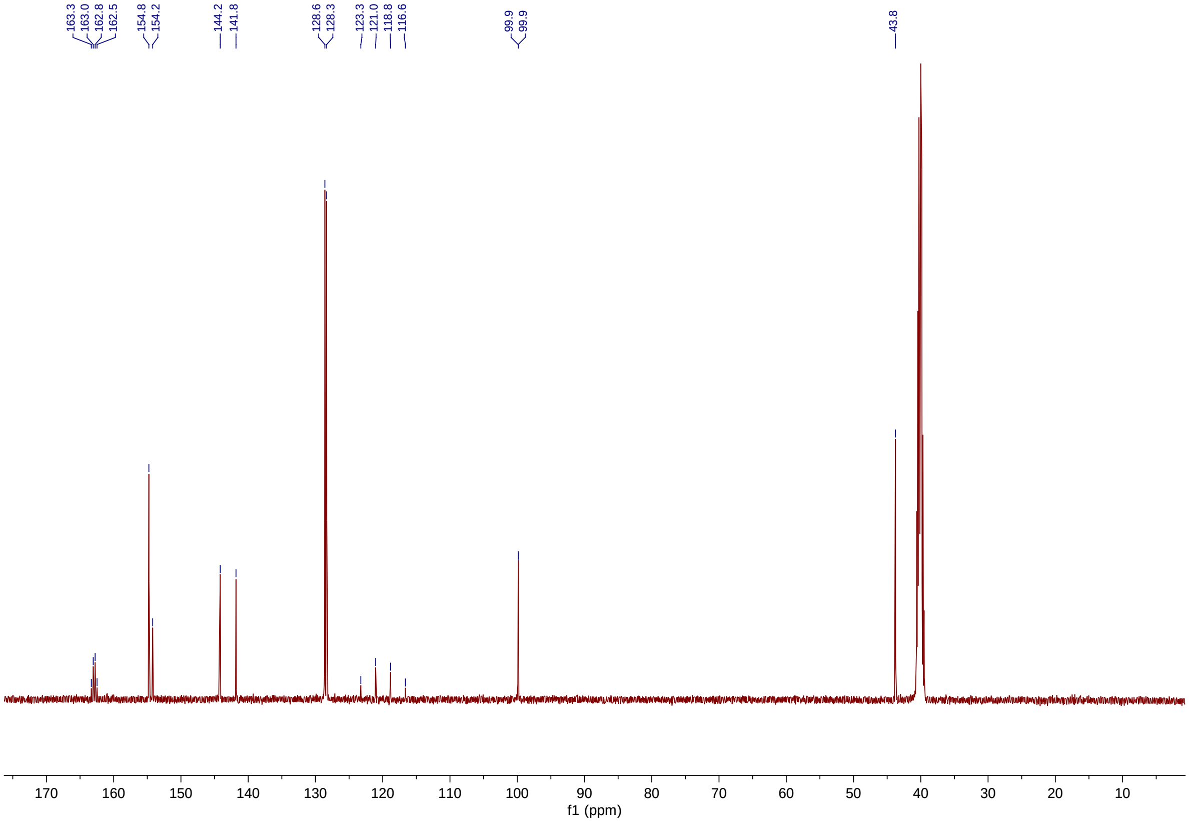

4-(2-Oxo-4-(trifluoromethyl)pyrimidin-1(2*H*)-yl)benzaldehyde (3k)

^{1}H NMR (400 MHz, DMSO-d_6):

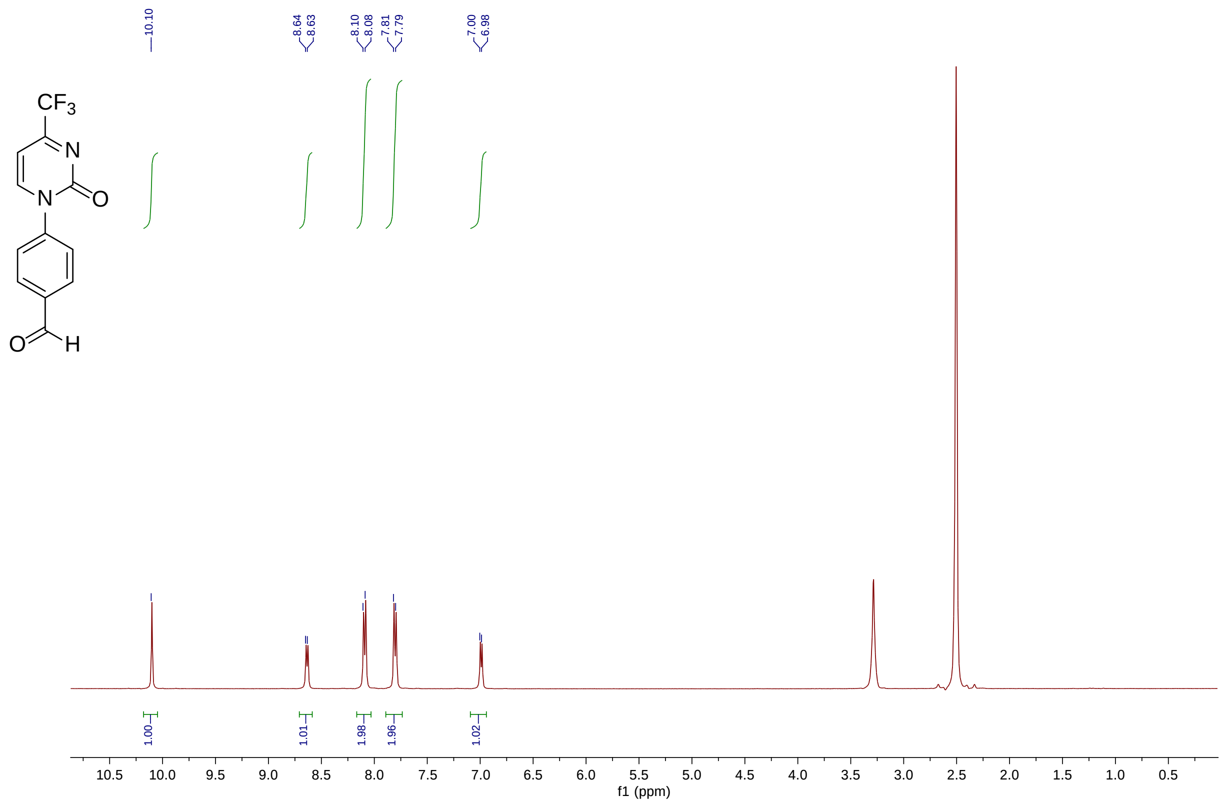

^{13}C NMR (150 MHz, DMSO-d_6):

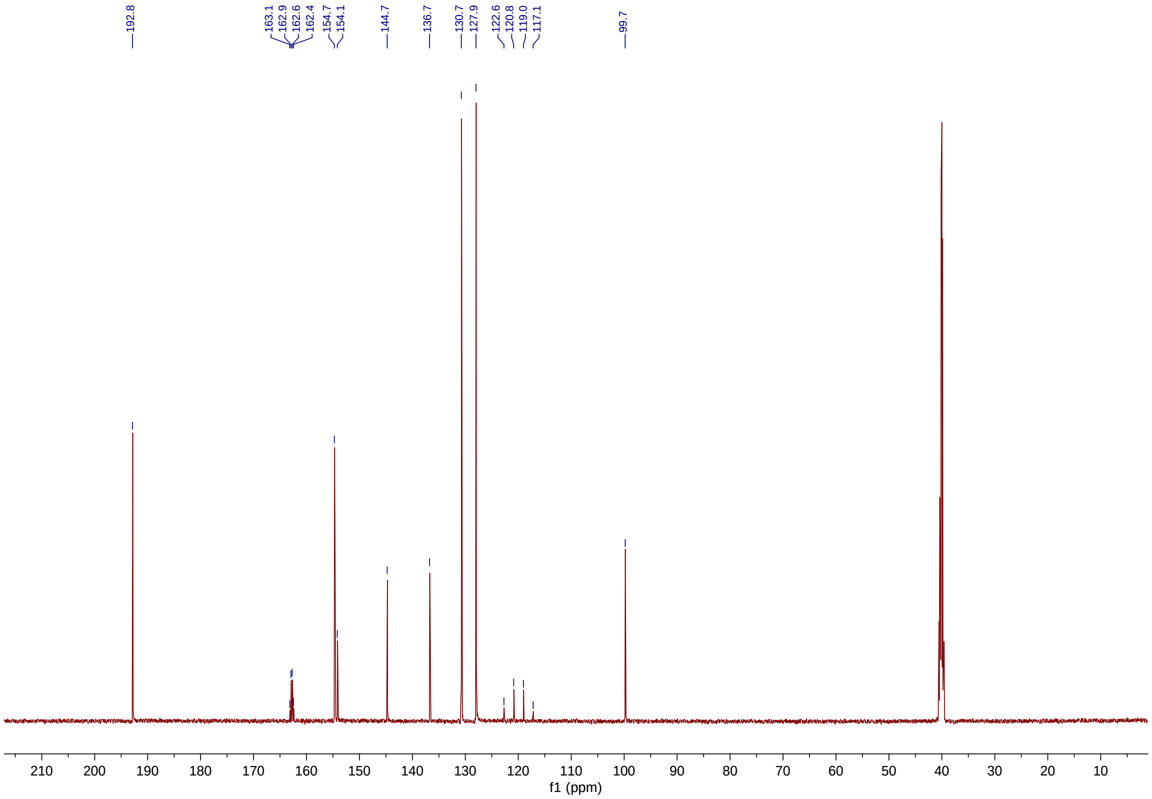

1-(3-Hydroxyphenyl)-4-(trifluoromethyl)pyrimidin-2(1*H*)-one (3l)

^{1}H NMR (400 MHz, DMSO-d_6):

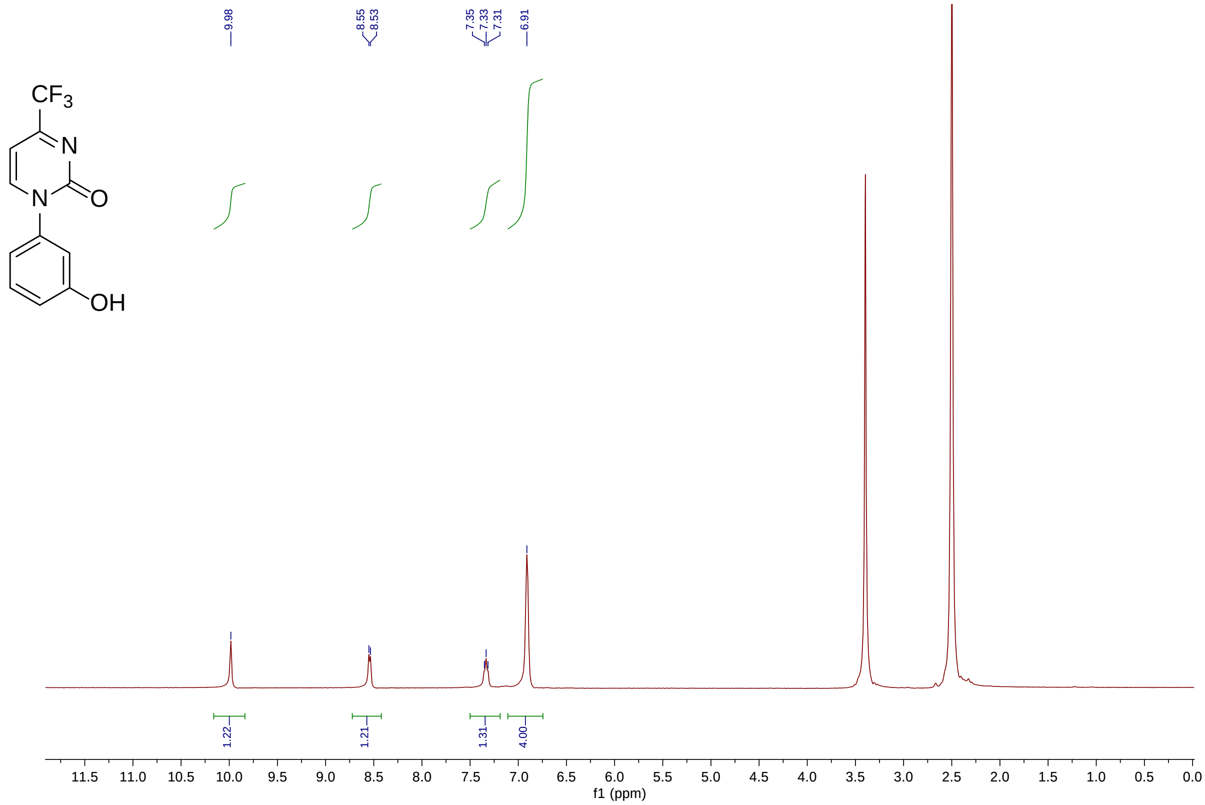

^{13}C NMR (125 MHz, DMSO-d_6):

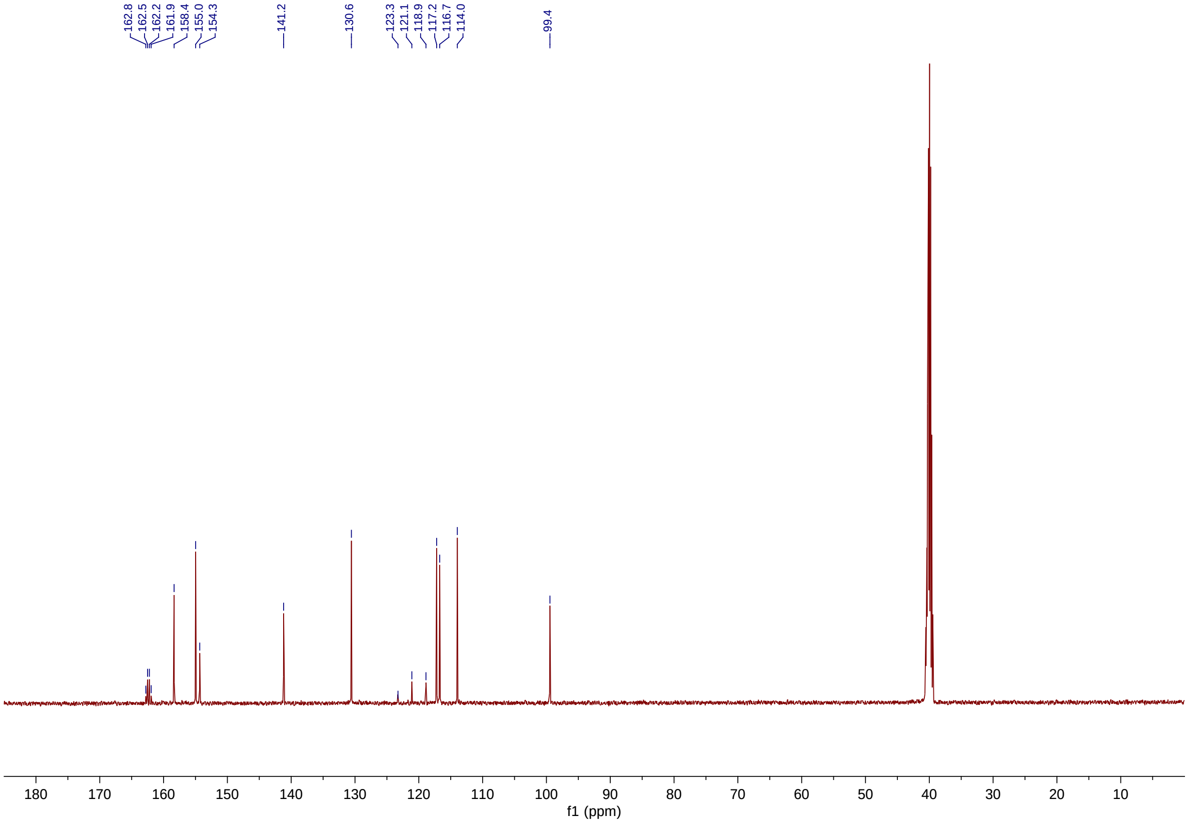

1-(4-Hydroxyphenyl)-4-(trifluoromethyl)pyrimidin-2(1*H*)-one (3m)

^{1}H NMR (400 MHz, DMSO-d_6):

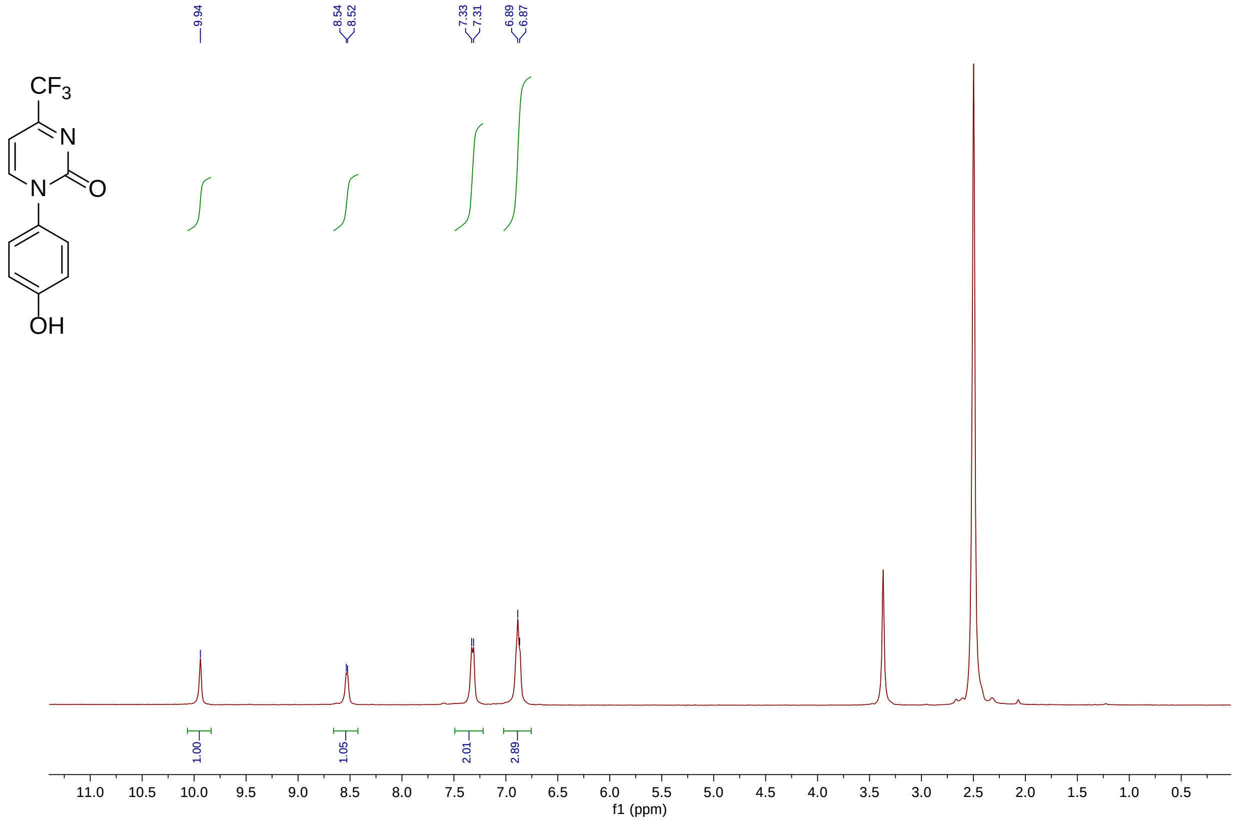

^{13}C NMR (150 MHz, DMSO-d_6):

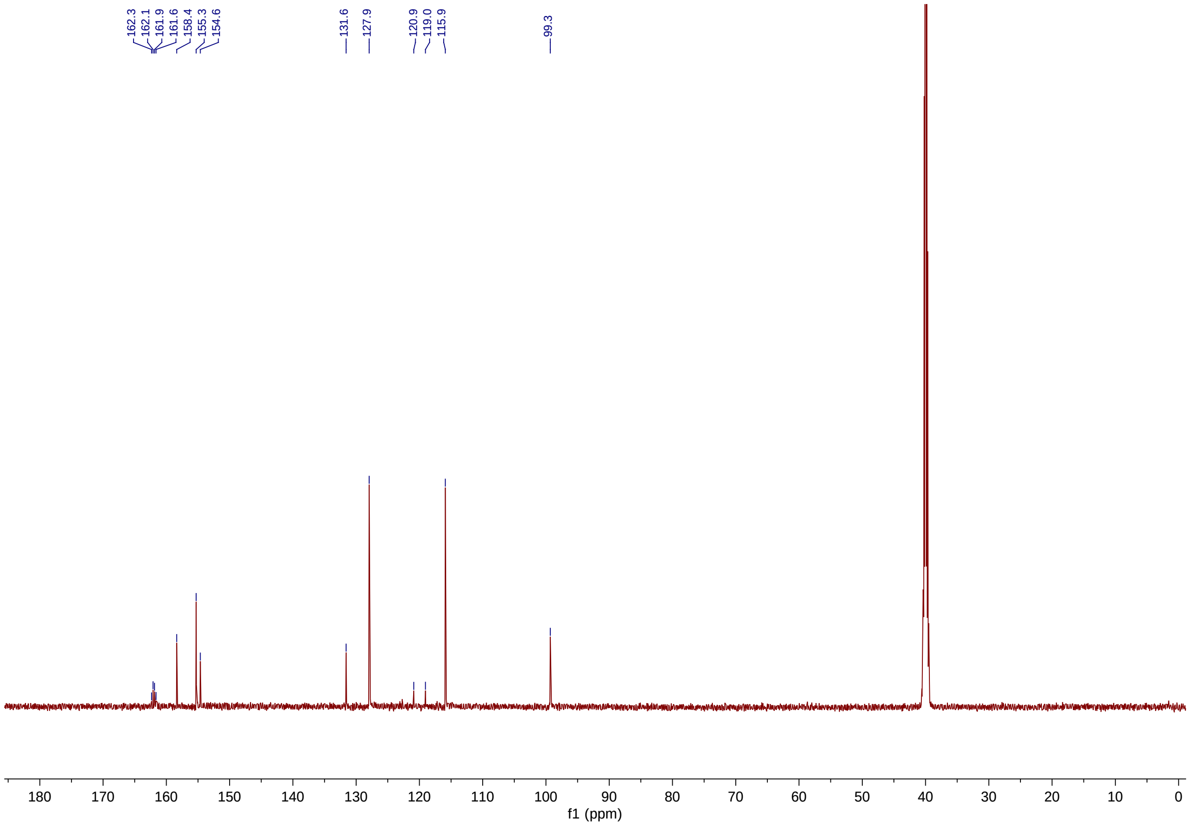

1-(4-(Hydroxymethyl)phenyl)-4-(trifluoromethyl)pyrimidin-2(1*H*)-one (3n)

[1]**H NMR** (400 MHz, DMSO-d_6):

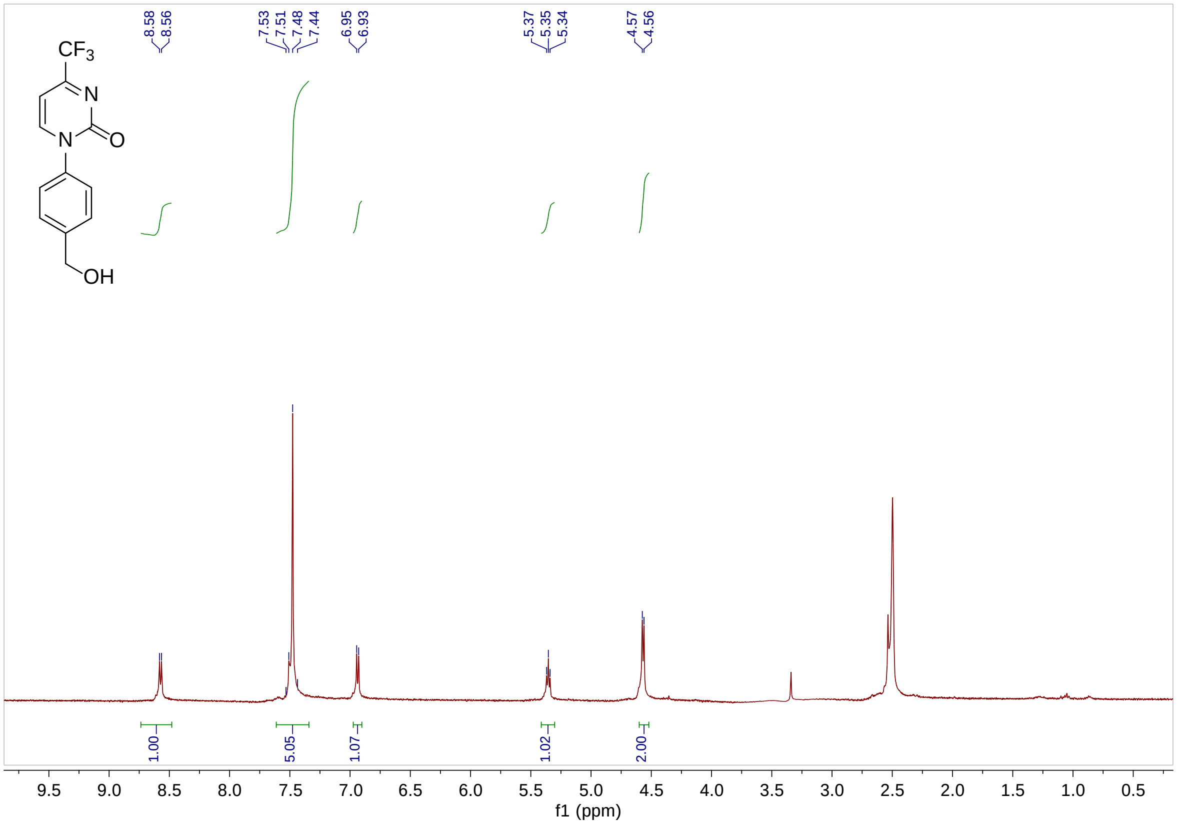

[13]**C NMR** (125 MHz, DMSO-d_6):

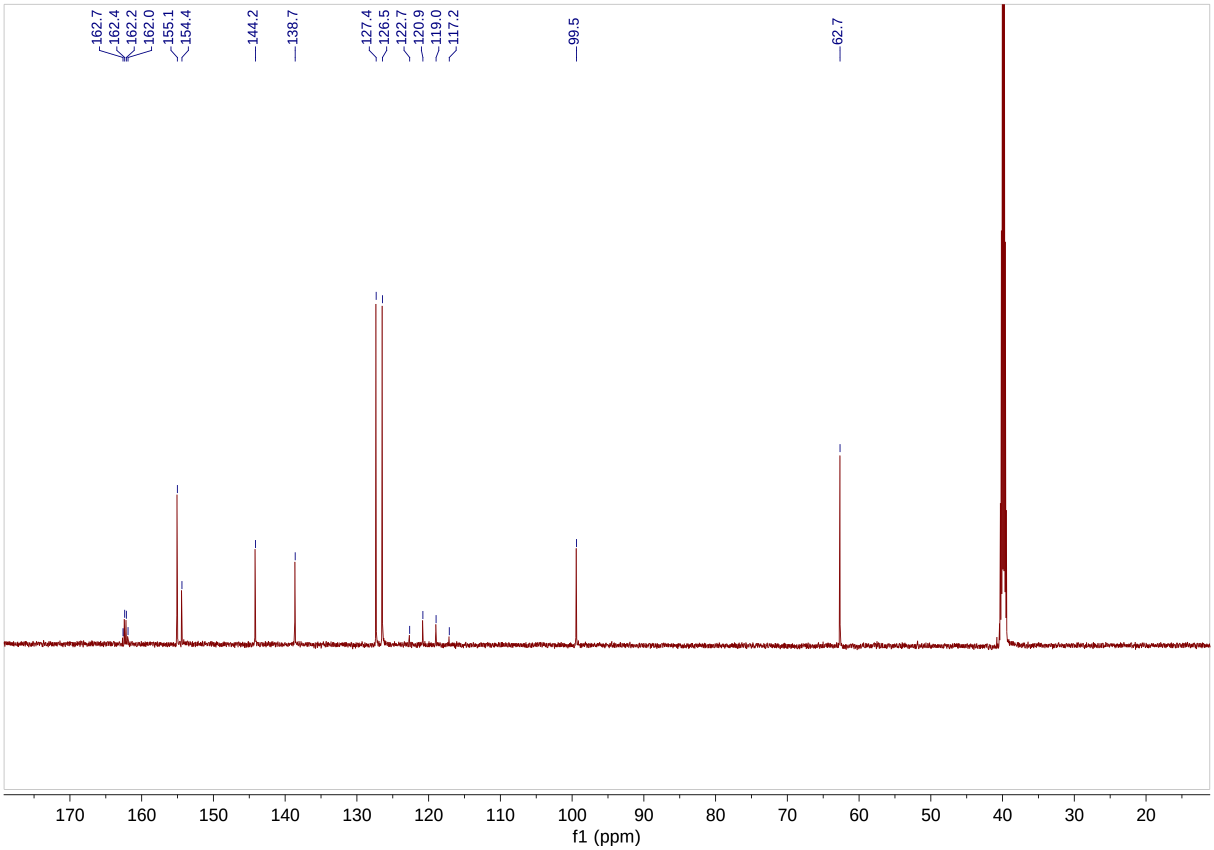

1-(2-Methoxyphenyl)-4-(trifluoromethyl)pyrimidin-2(1*H*)-one (3o)

^{1}H NMR (400 MHz, DMSO-d_6):

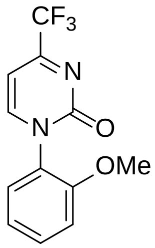

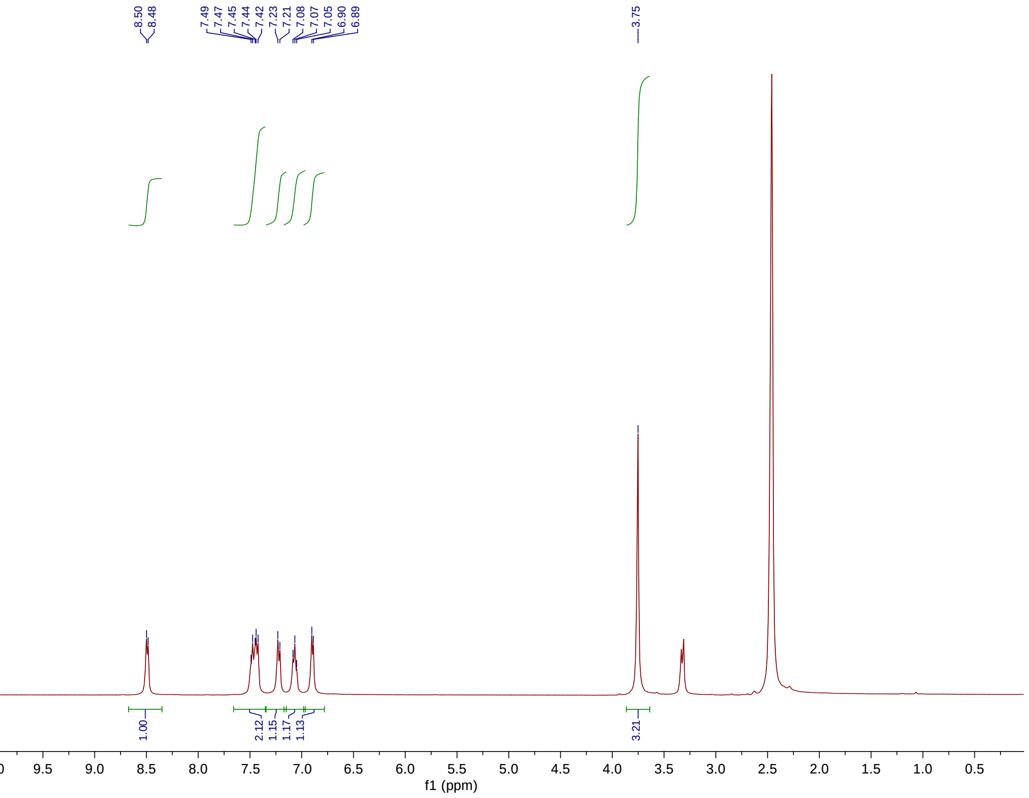

^{13}C NMR (100 MHz, DMSO-d_6):

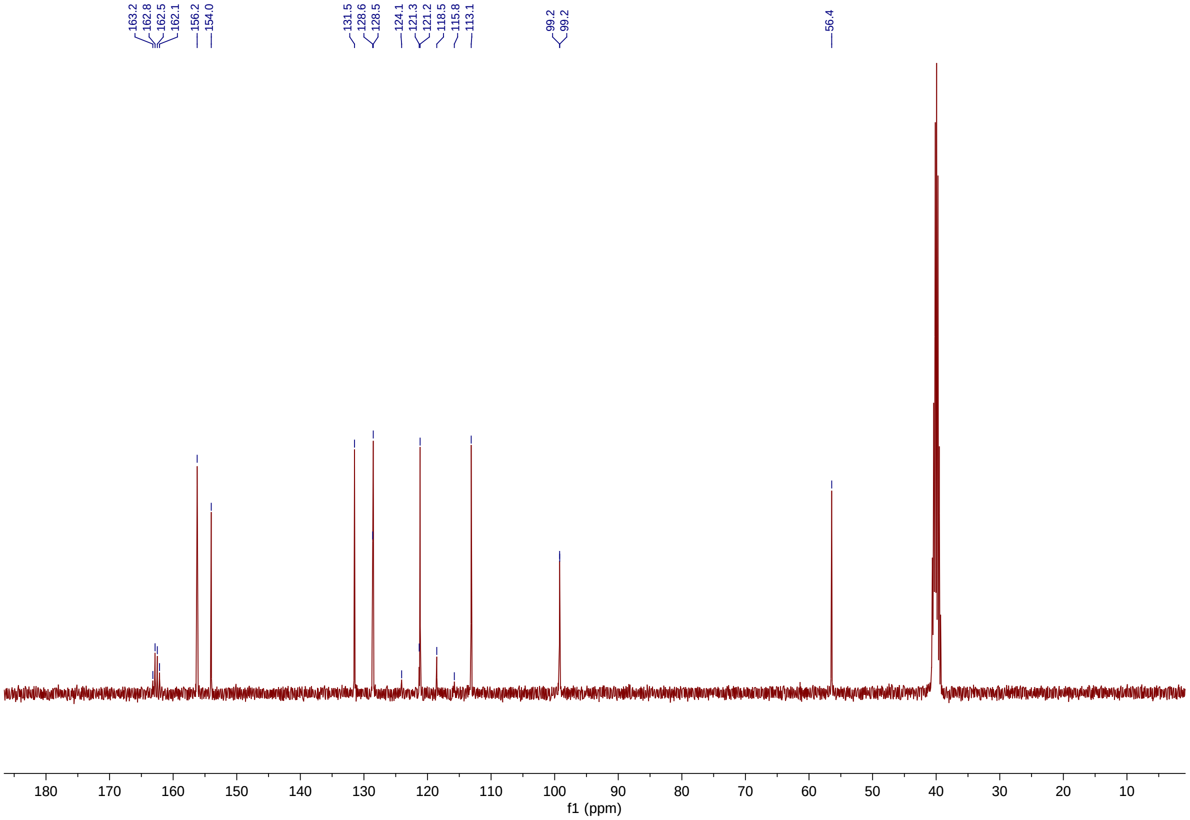

1-(o-Tolyl)-4-(trifluoromethyl)pyrimidin-2(1*H*)-one (3p)

^{1}H NMR (400 MHz, DMSO-d_6):

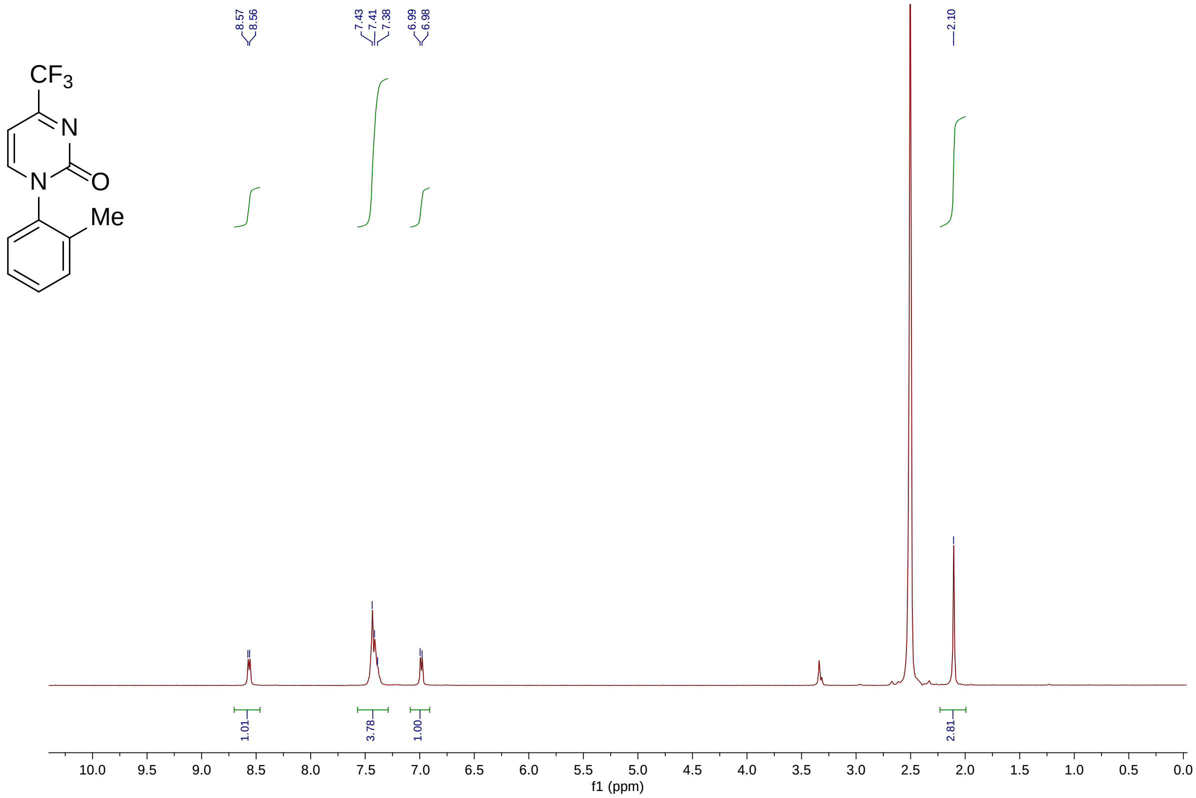

^{13}C NMR (100 MHz, DMSO-d_6):

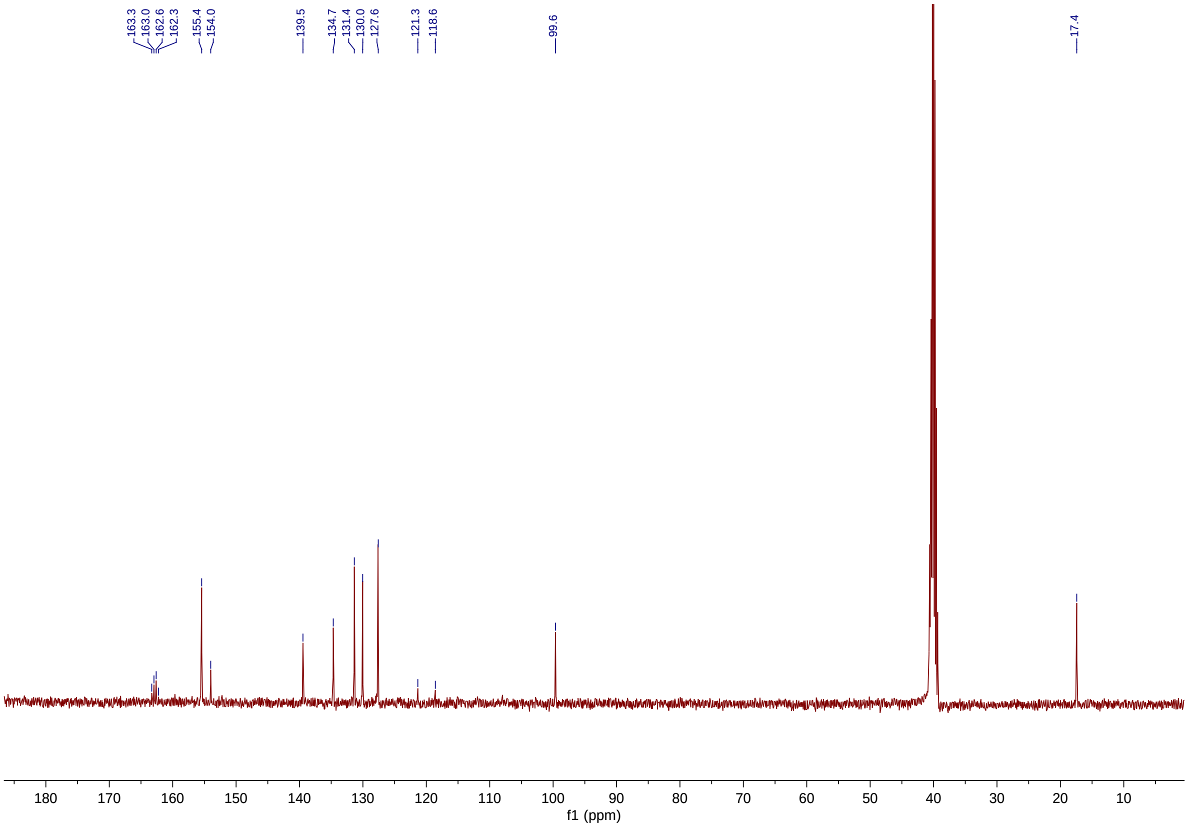

1-(Pyridin-3-yl)-4-(trifluoromethyl)pyrimidin-2(1*H*)-one (3q)

^{1}H NMR (400 MHz, DMSO-d_6):

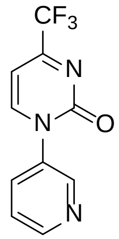

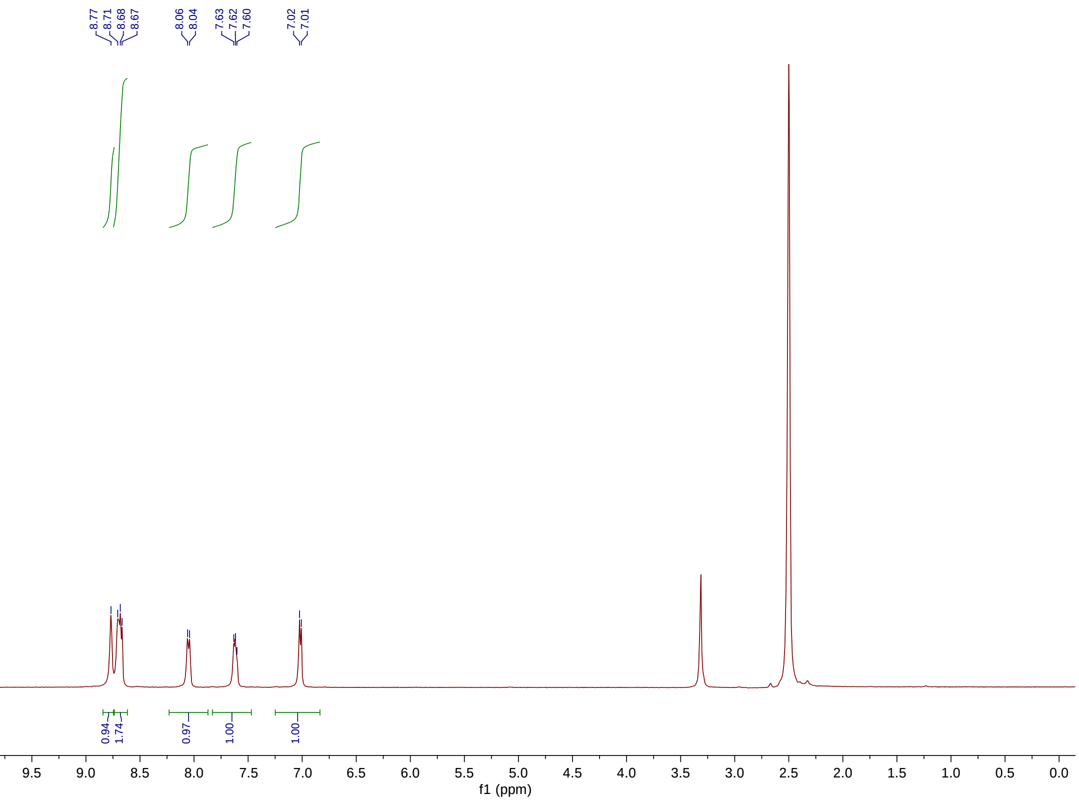

^{13}C NMR (125 MHz, DMSO-d_6):

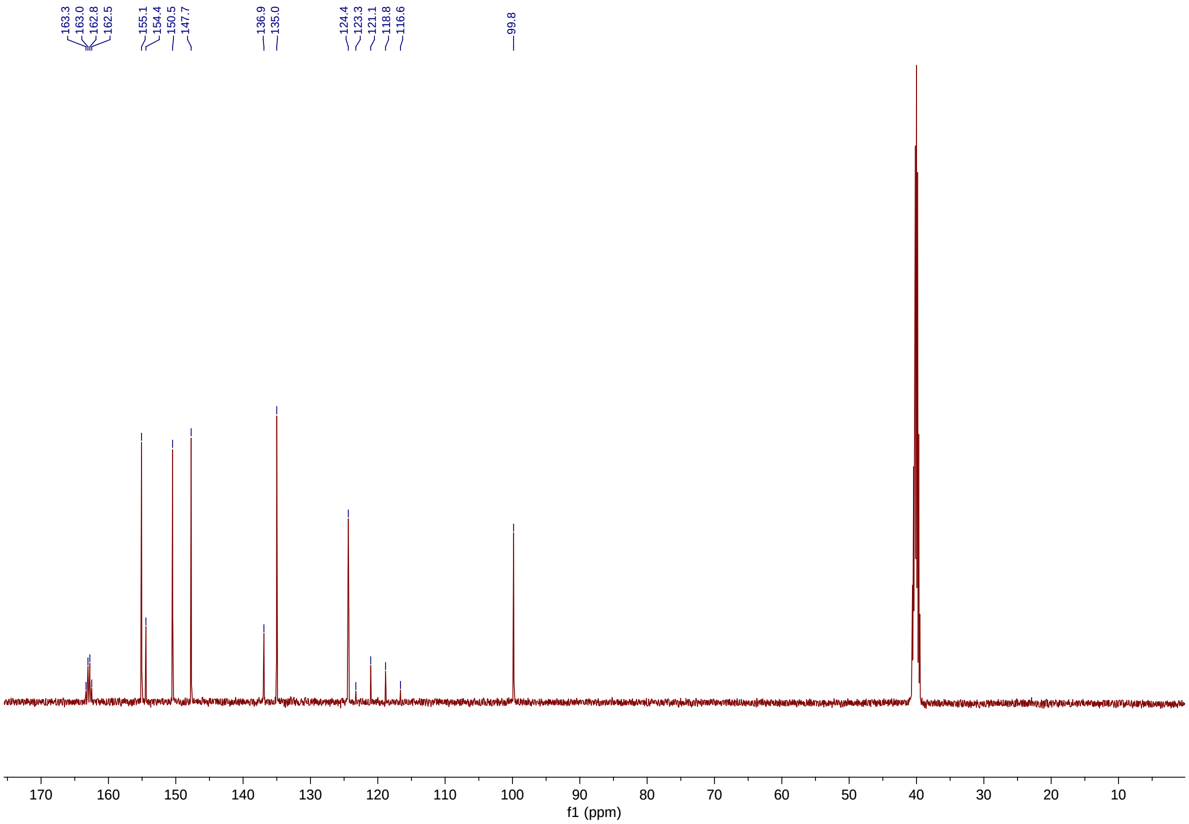

1-(Pyridin-4-yl)-4-(trifluoromethyl)pyrimidin-2(1*H*)-one (3r)

^{1}H NMR (400 MHz, DMSO-d_6):

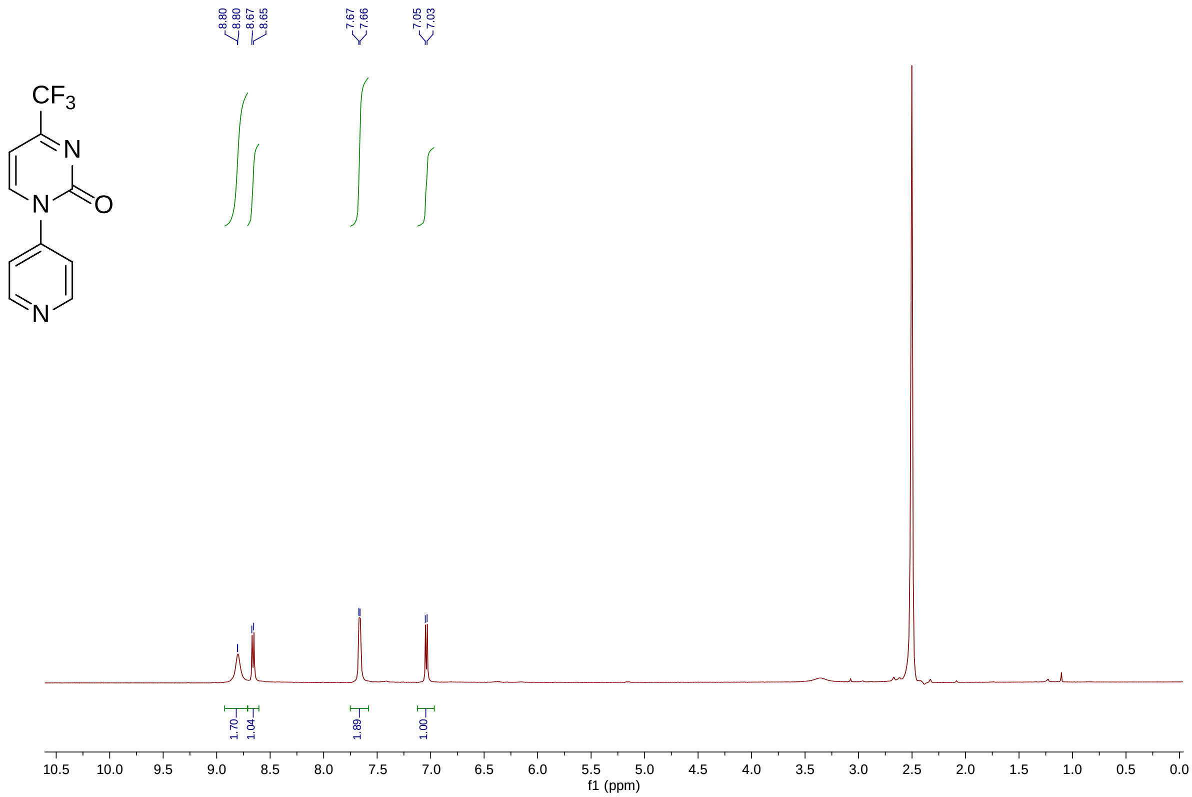

^{13}C NMR (150 MHz, DMSO-d_6):

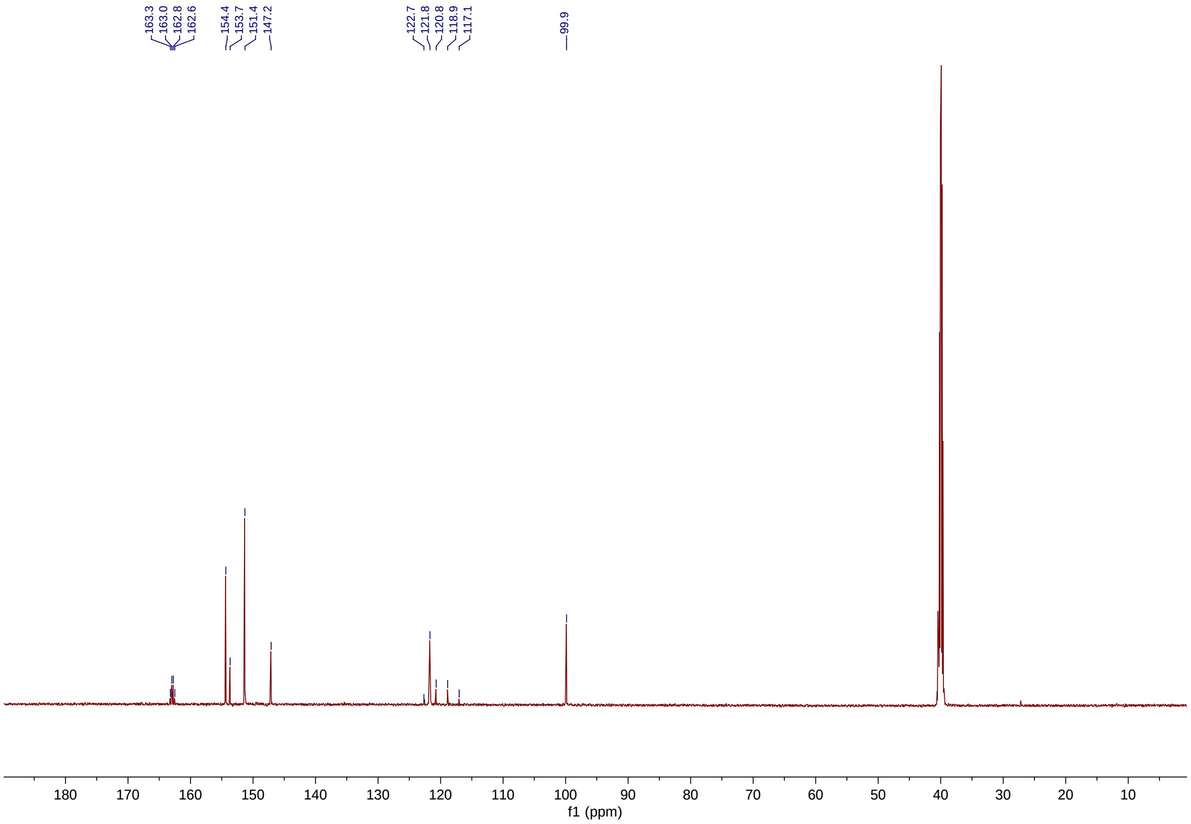

1-(6-Methoxypyridin-3-yl)-4-(trifluoromethyl)pyrimidin-2(1*H*)-one (3s)

^{1}H NMR (400 MHz, DMSO-d_6):

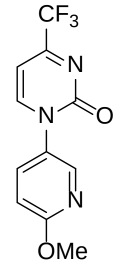

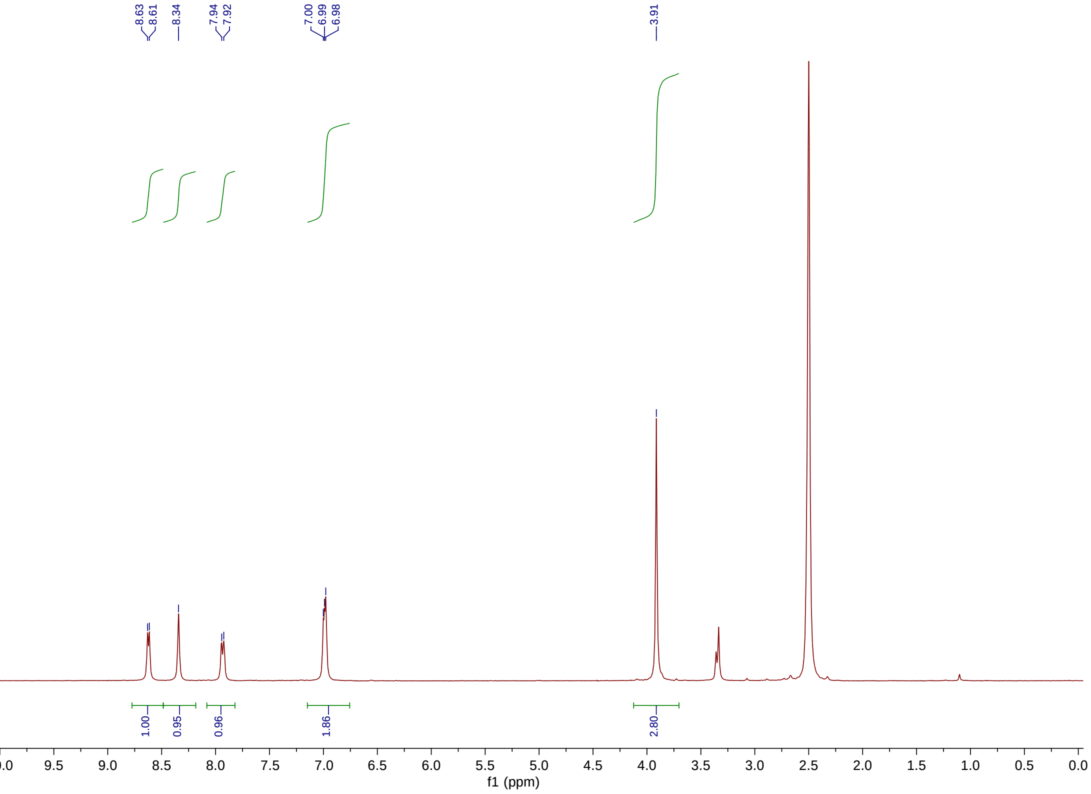

^{13}C NMR (125 MHz, DMSO-d_6):

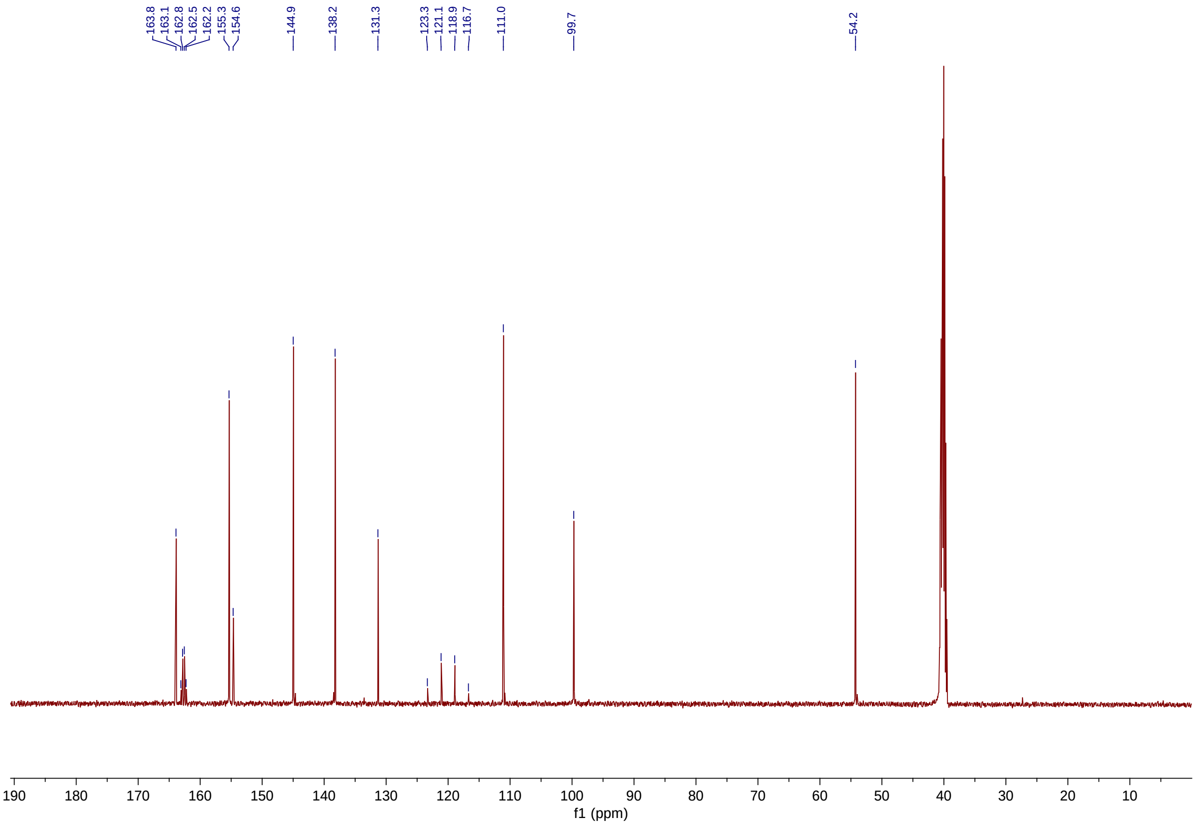

1-(6-(Pyrrolidin-1-yl)pyridin-3-yl)-4-(trifluoromethyl)pyrimidin-2(1*H*)-one (3t)

^{1}H NMR (400 MHz, DMSO-d_6):

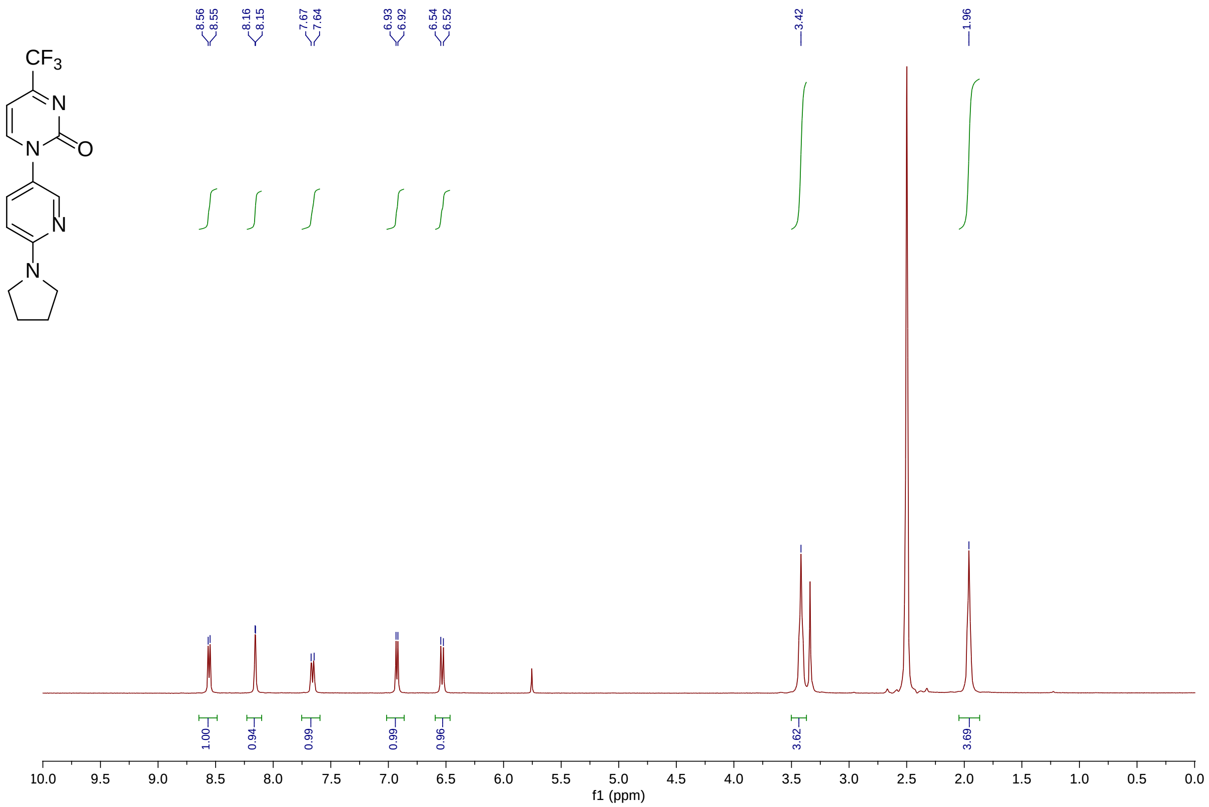

^{13}C NMR (125 MHz, DMSO-d_6):

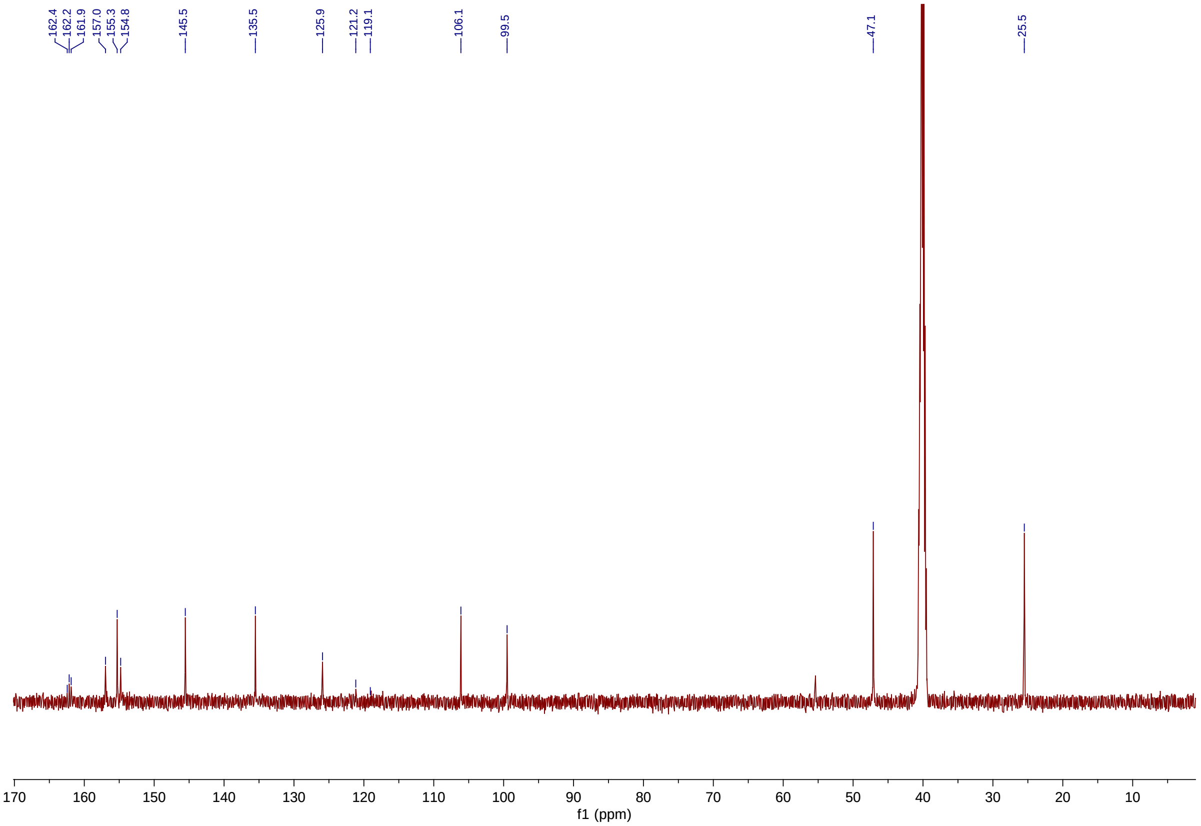

1-(Thiophen-3-yl)-4-(trifluoromethyl)pyrimidin-2(1*H*)-one (3u)

^{1}H NMR (400 MHz, DMSO-d_6):

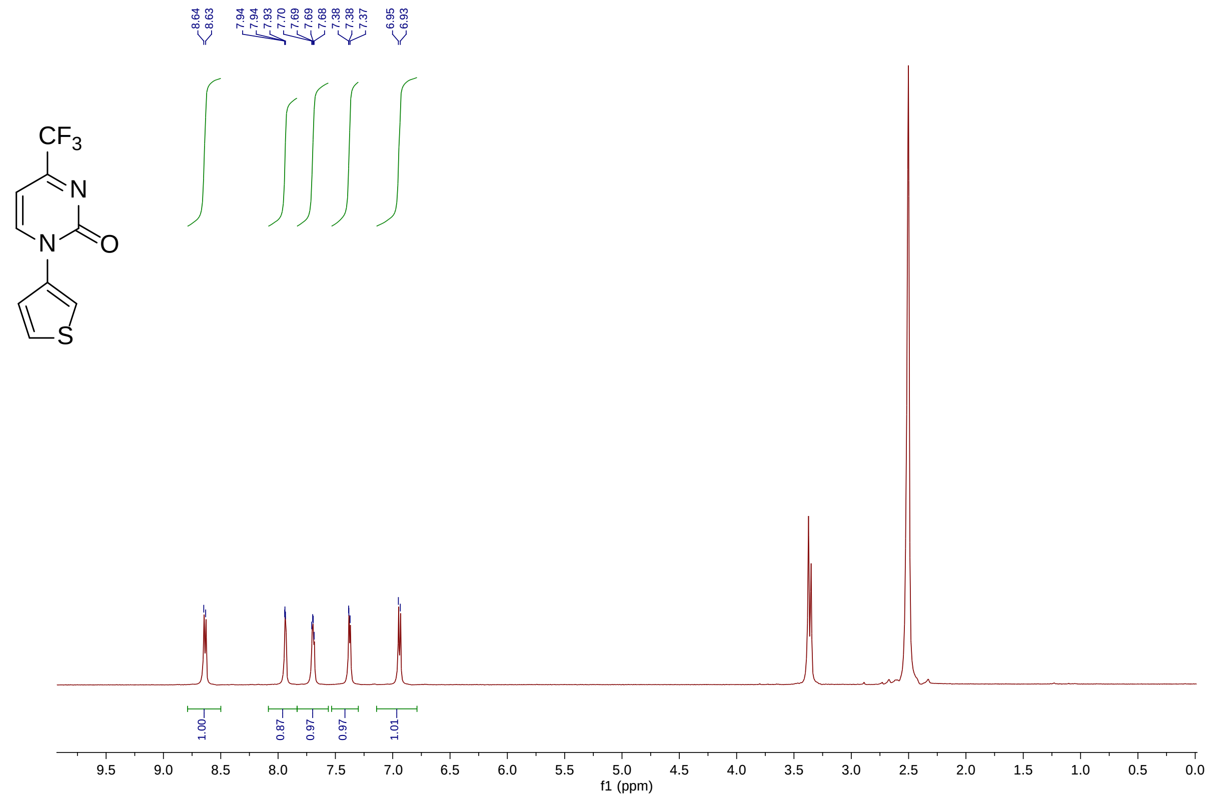

^{13}C NMR (125 MHz, DMSO-d_6):

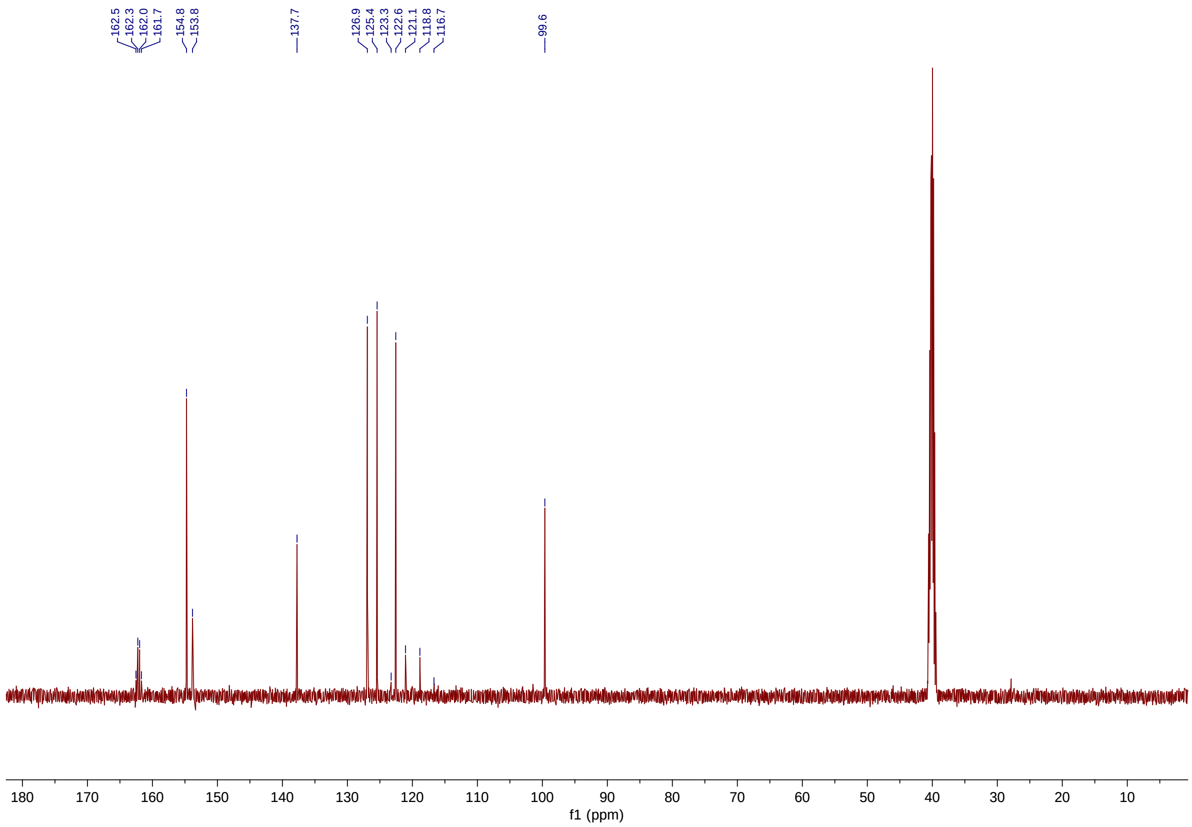

4-(Trifluoromethyl)-1-(5-(trifluoromethyl)thiophen-3-yl)pyrimidin-2(1*H*)-one (3v).

^{1}H NMR (400 MHz, DMSO-d_6):

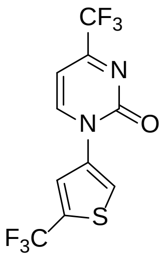

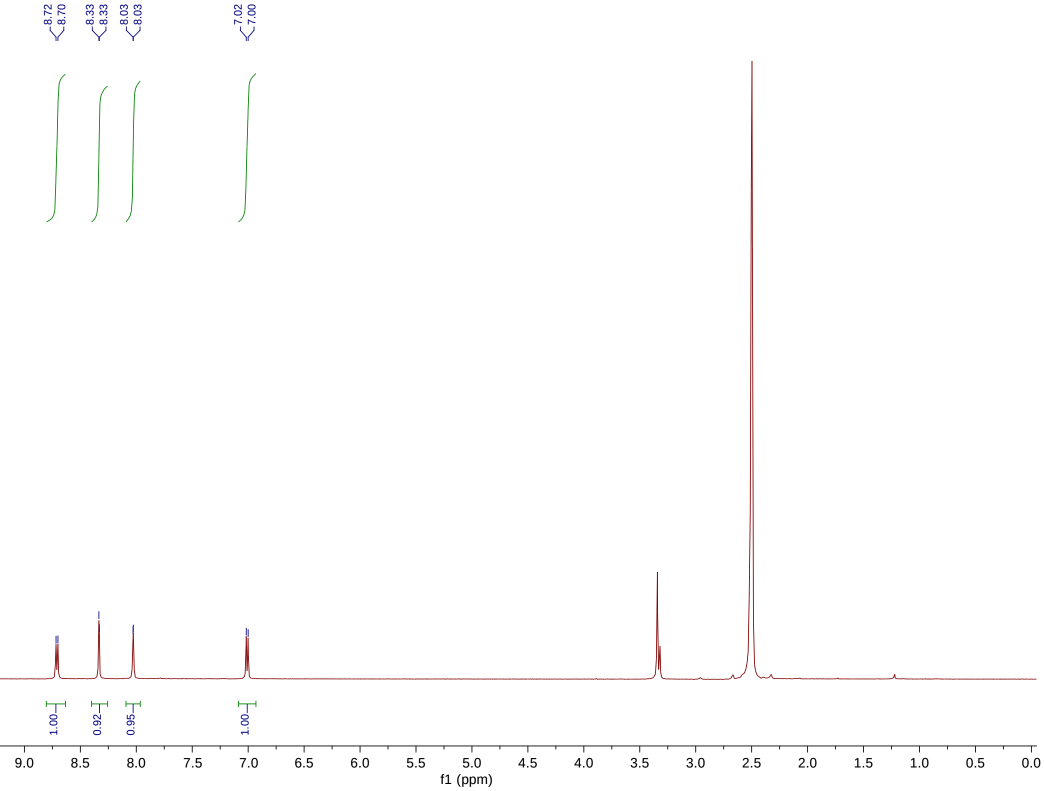

^{13}C NMR (125 MHz, DMSO-d_6):

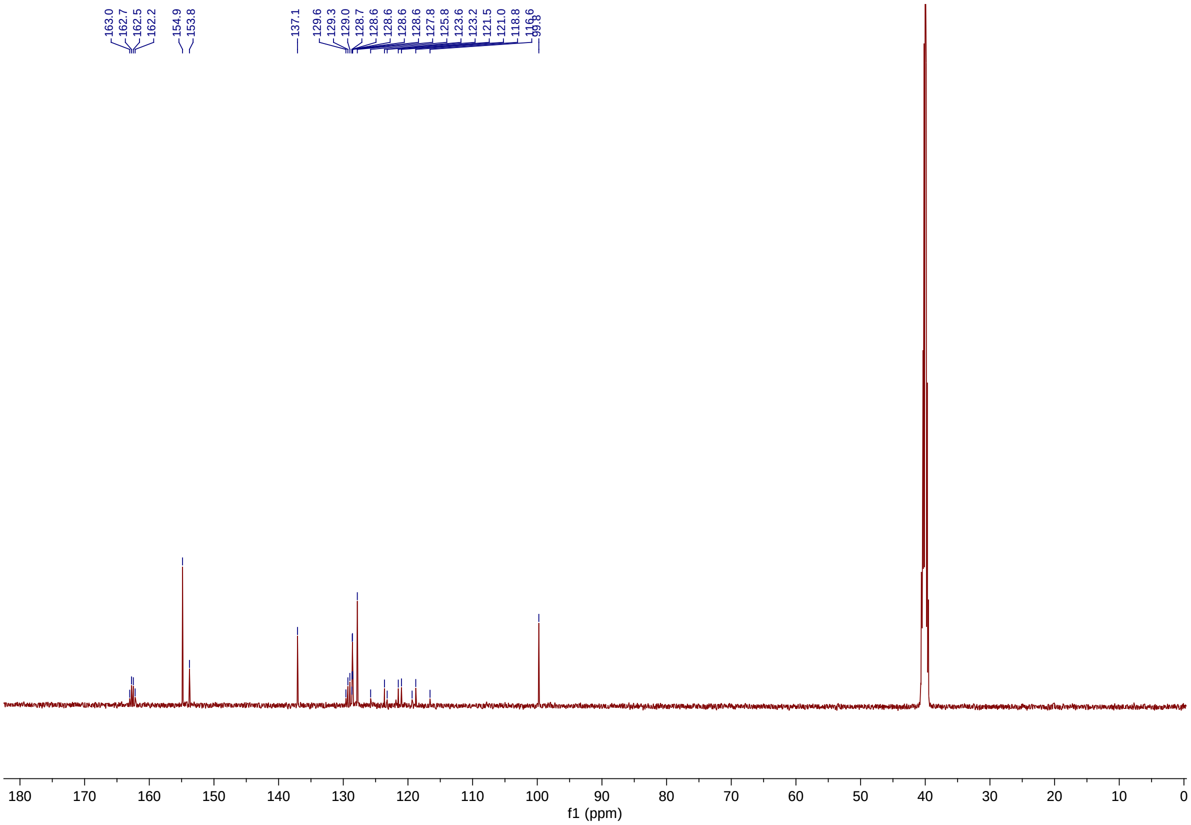

1-(Furan-3-yl)-4-(trifluoromethyl)pyrimidin-2(1*H*)-one (3w)

^{1}H NMR (400 MHz, DMSO-d_6):

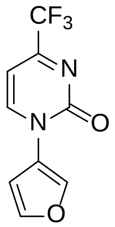

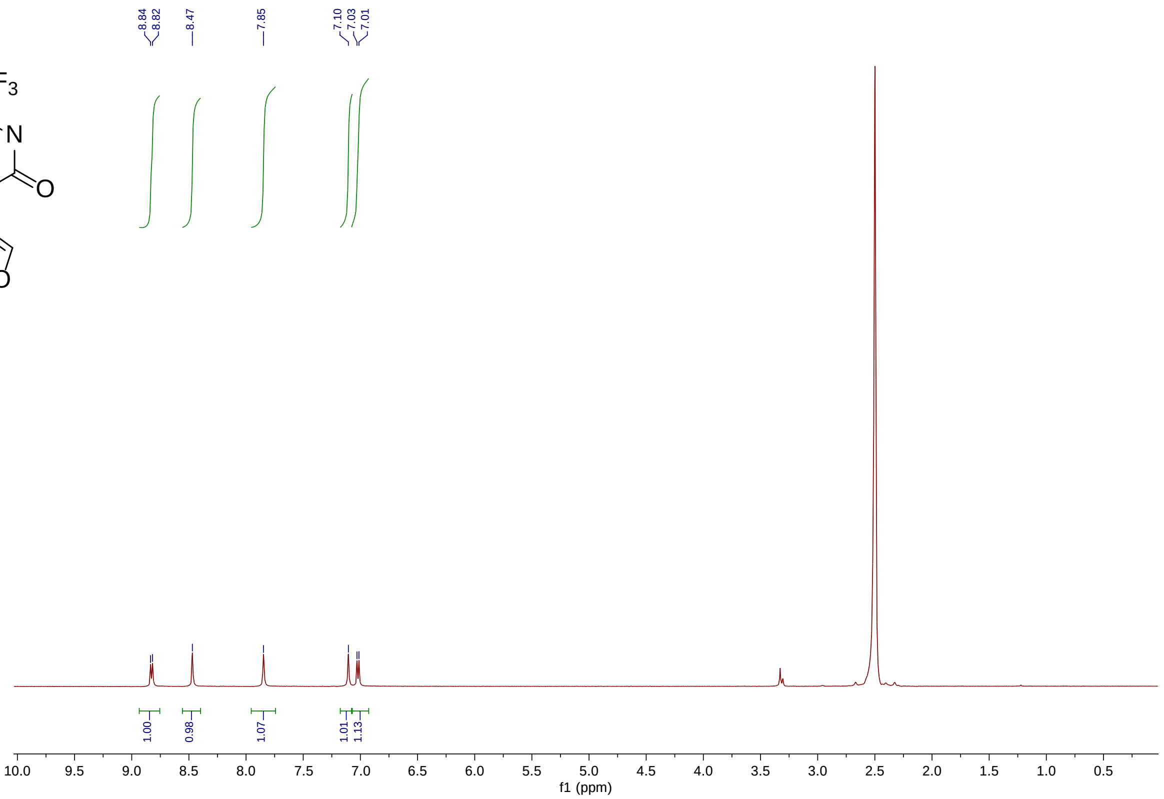

^{13}C NMR (100 MHz, DMSO-d_6):

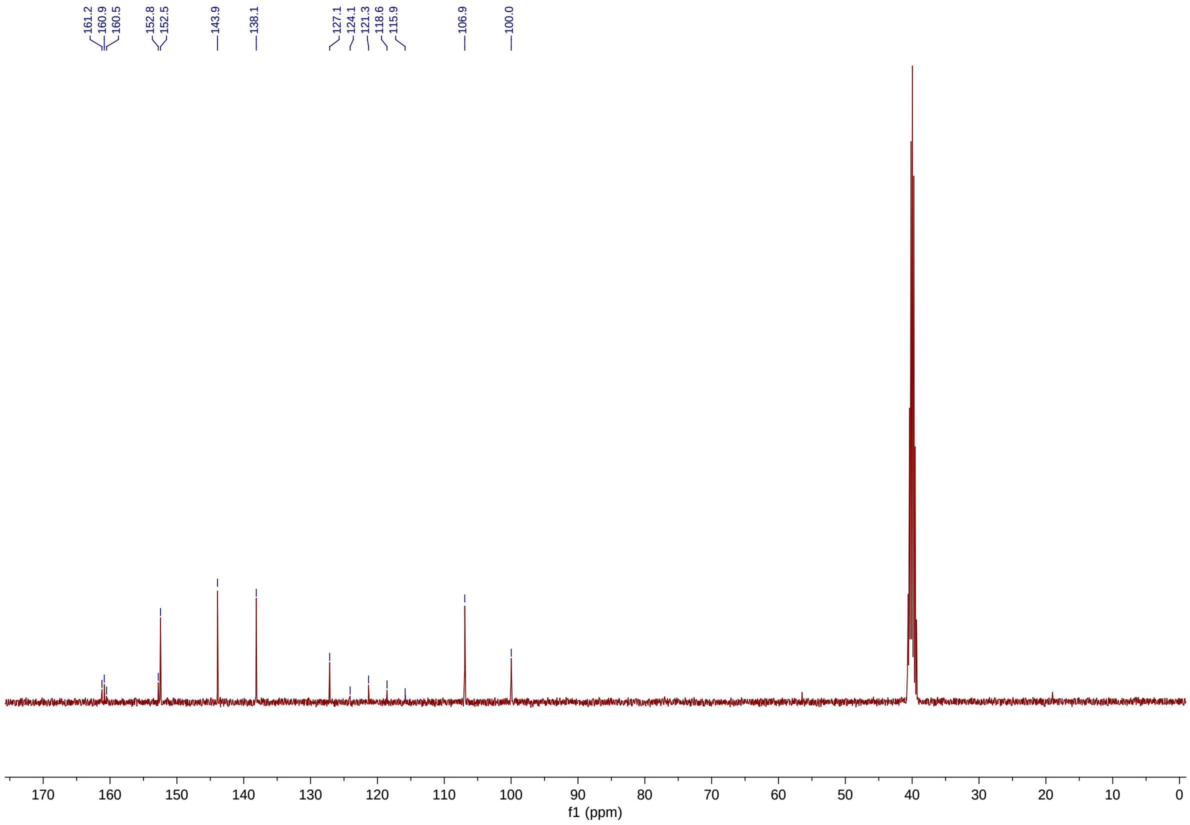

(*E*)-1-Styryl-4-(trifluoromethyl)pyrimidin-2(1*H*)-one (5a)

^{1}H NMR (400 MHz, DMSO-d_6):

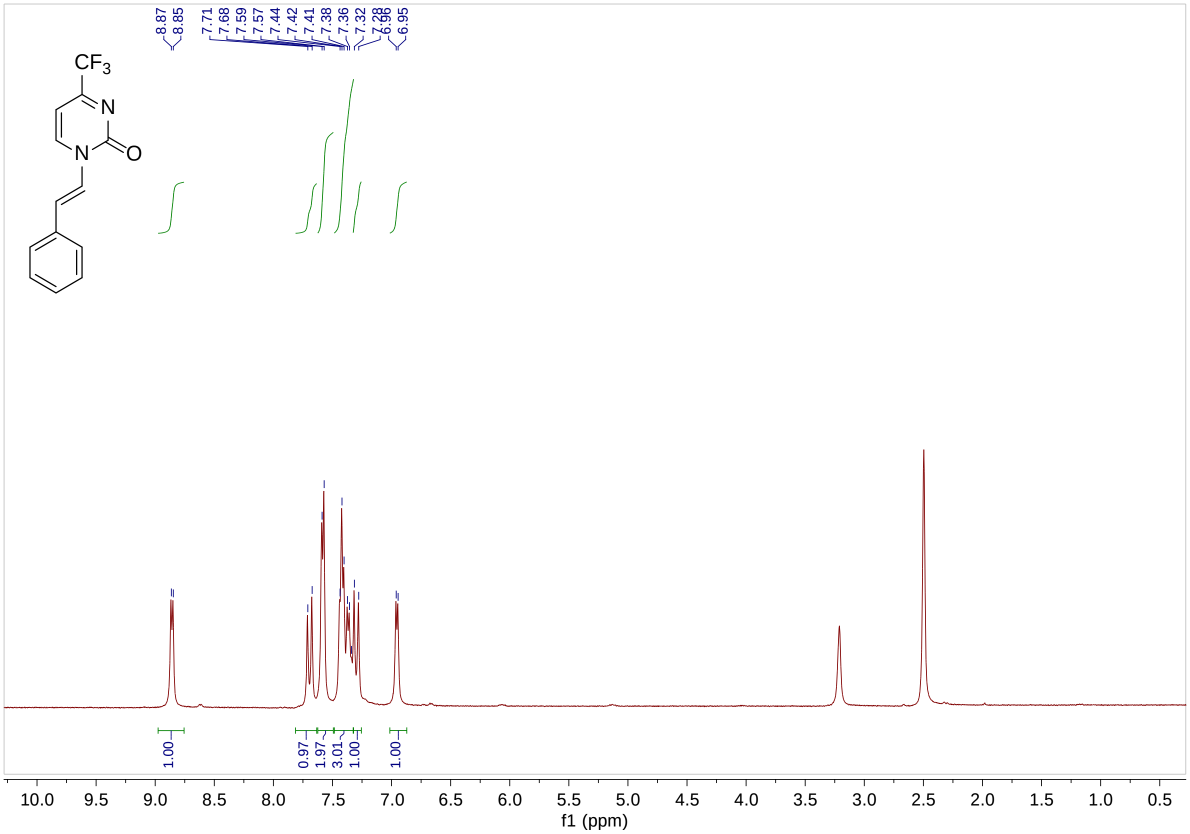

^{13}C NMR (125 MHz, DMSO-d_6):

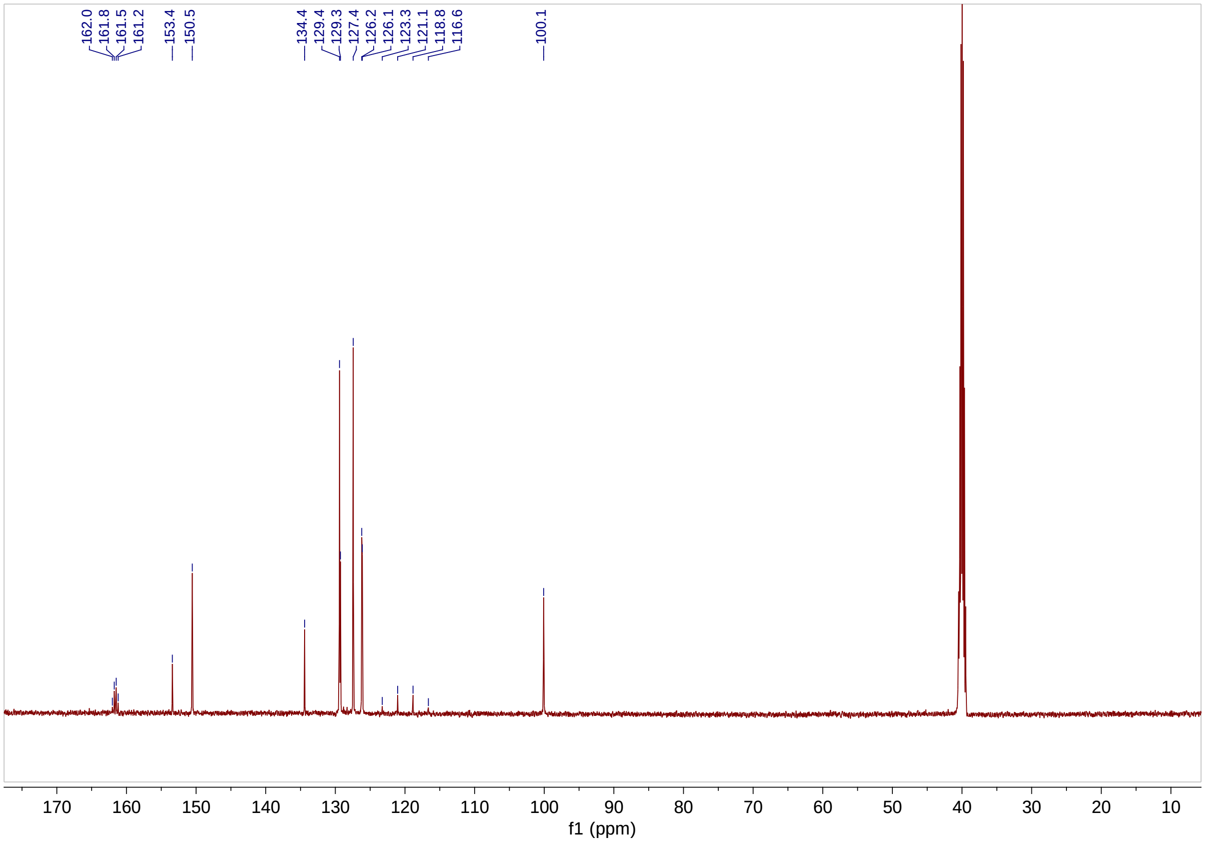

NOE experiment

^{1}H NMR (600 MHz, DMSO-d_6):

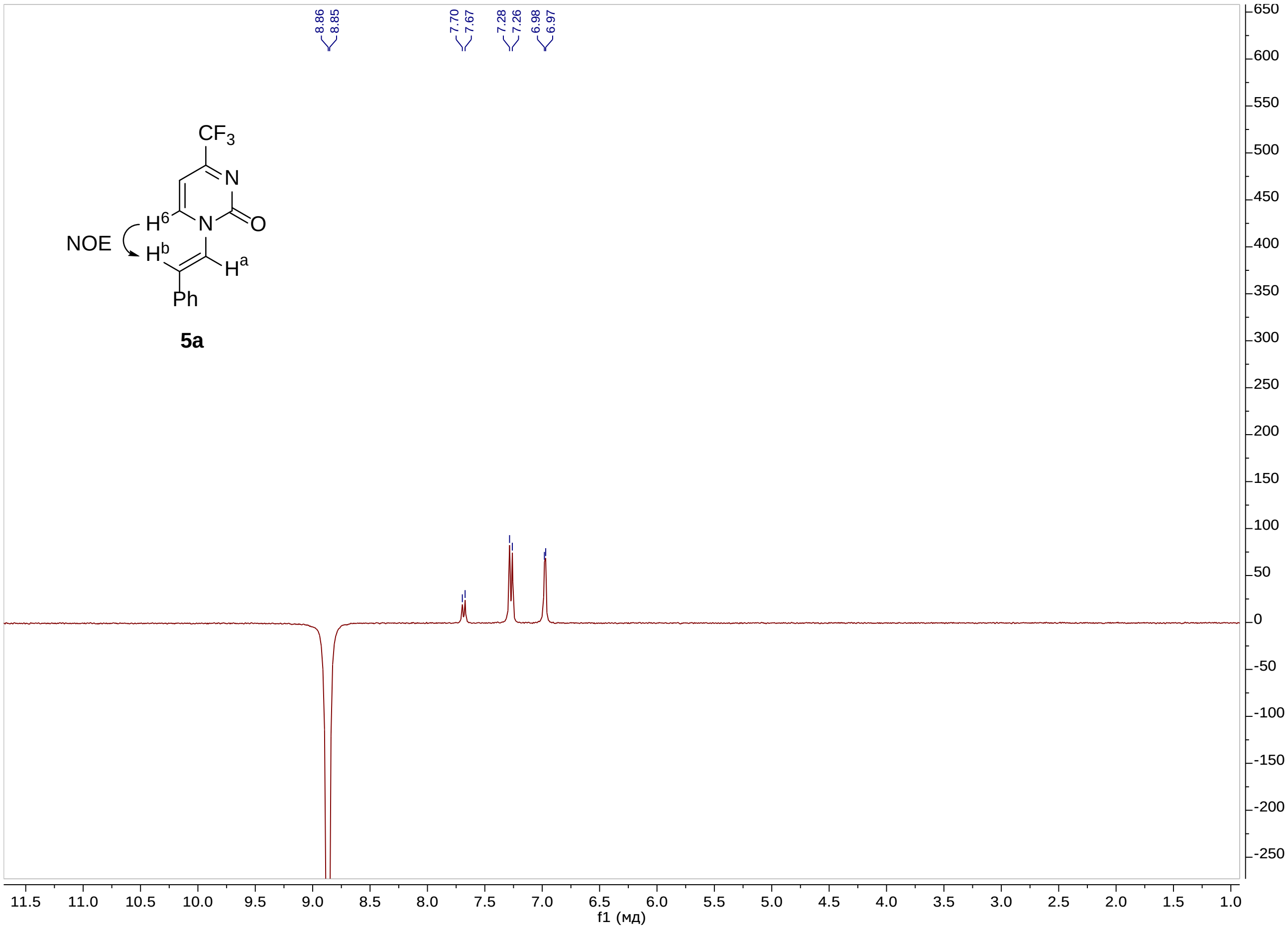

(*E*)-1-(3,5-Difluorostyryl)-4-(trifluoromethyl)pyrimidin-2(1*H*)-one (5b)

^{1}H NMR (400 MHz, DMSO-d_6):

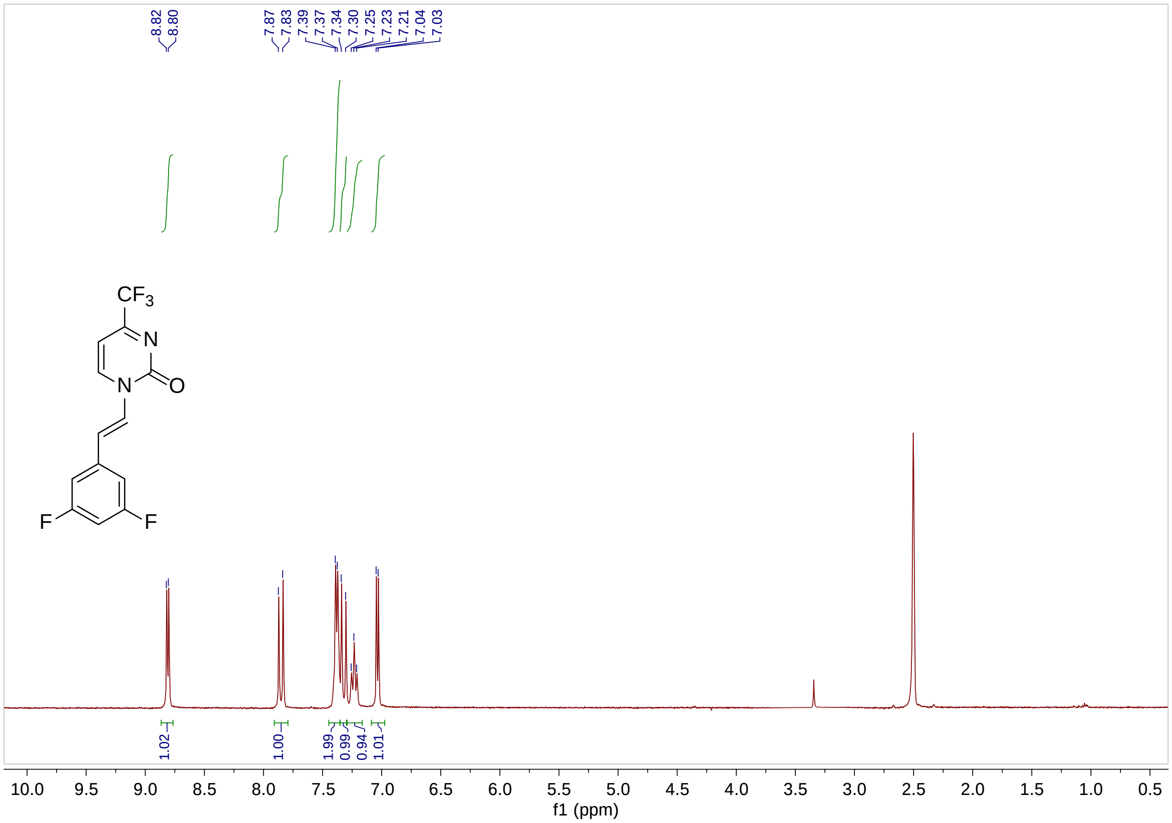

^{13}C NMR (125 MHz, DMSO-d_6):

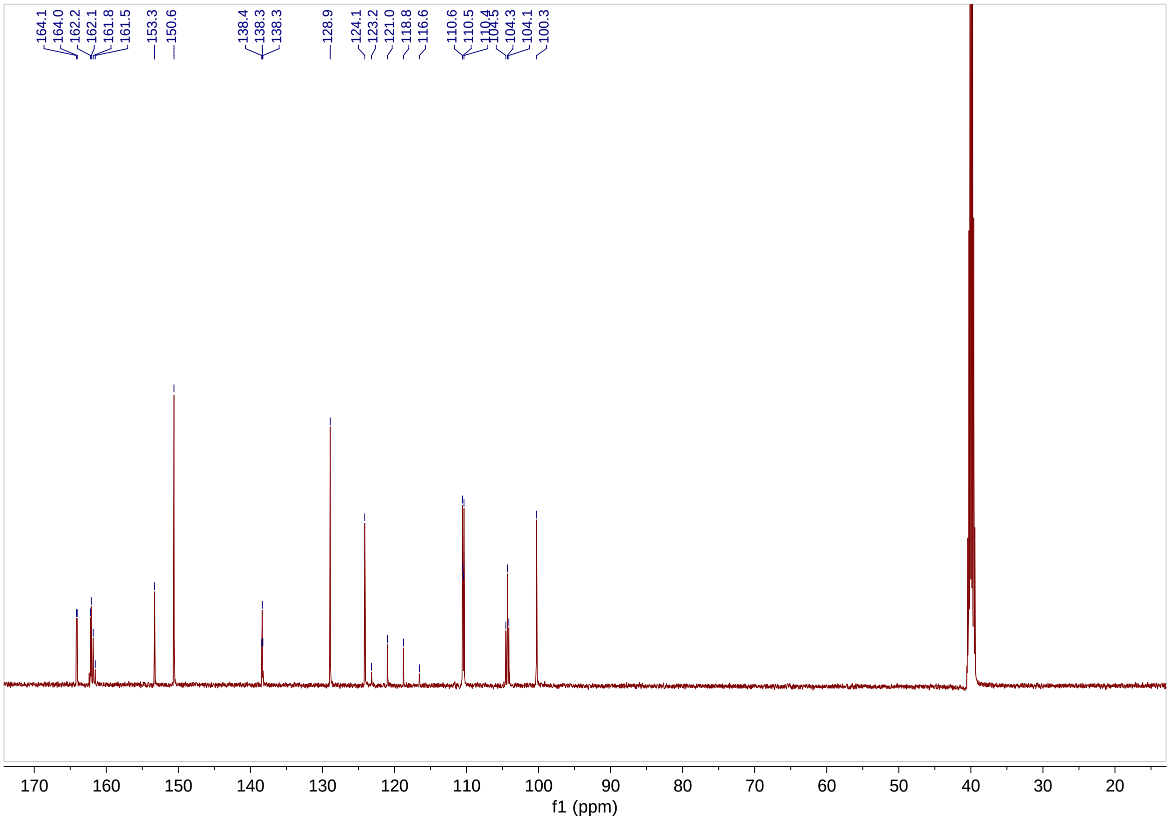

(*E*)-1-(3-Methoxystyryl)-4-(trifluoromethyl)pyrimidin-2(1*H*)-one (5c)

^{1}H NMR (400 MHz, DMSO-d_6):

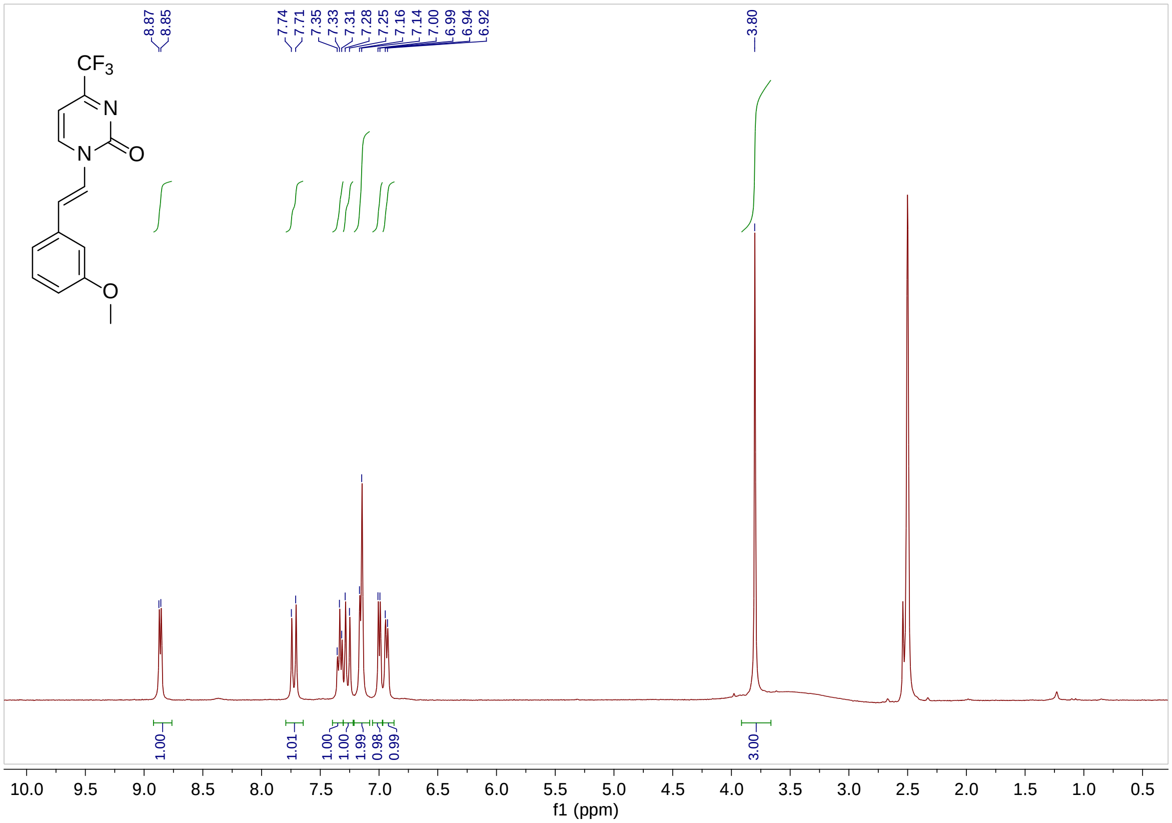

^{13}C NMR (125 MHz, DMSO-d_6):

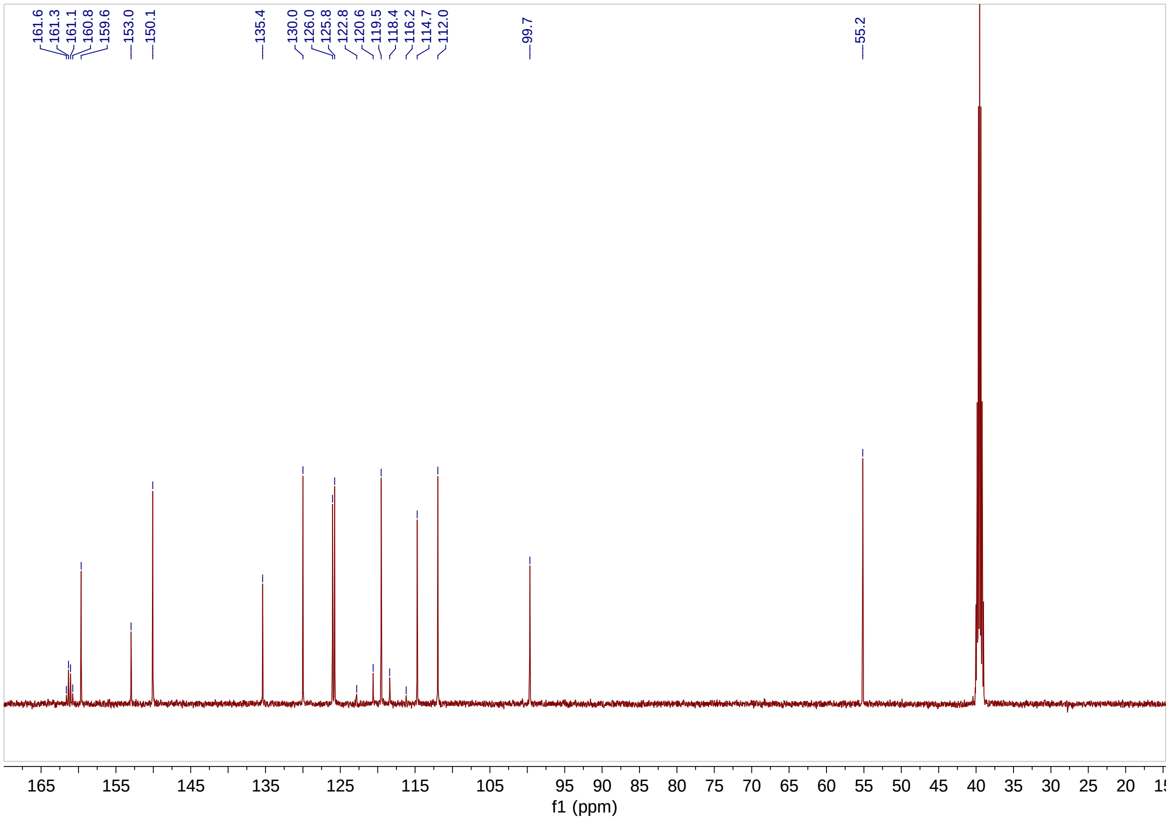

(*E*)-1-(4-Fluorostyryl)-4-(trifluoromethyl)pyrimidin-2(1*H*)-one (5d)

^{1}H NMR (400 MHz, DMSO-d_6):

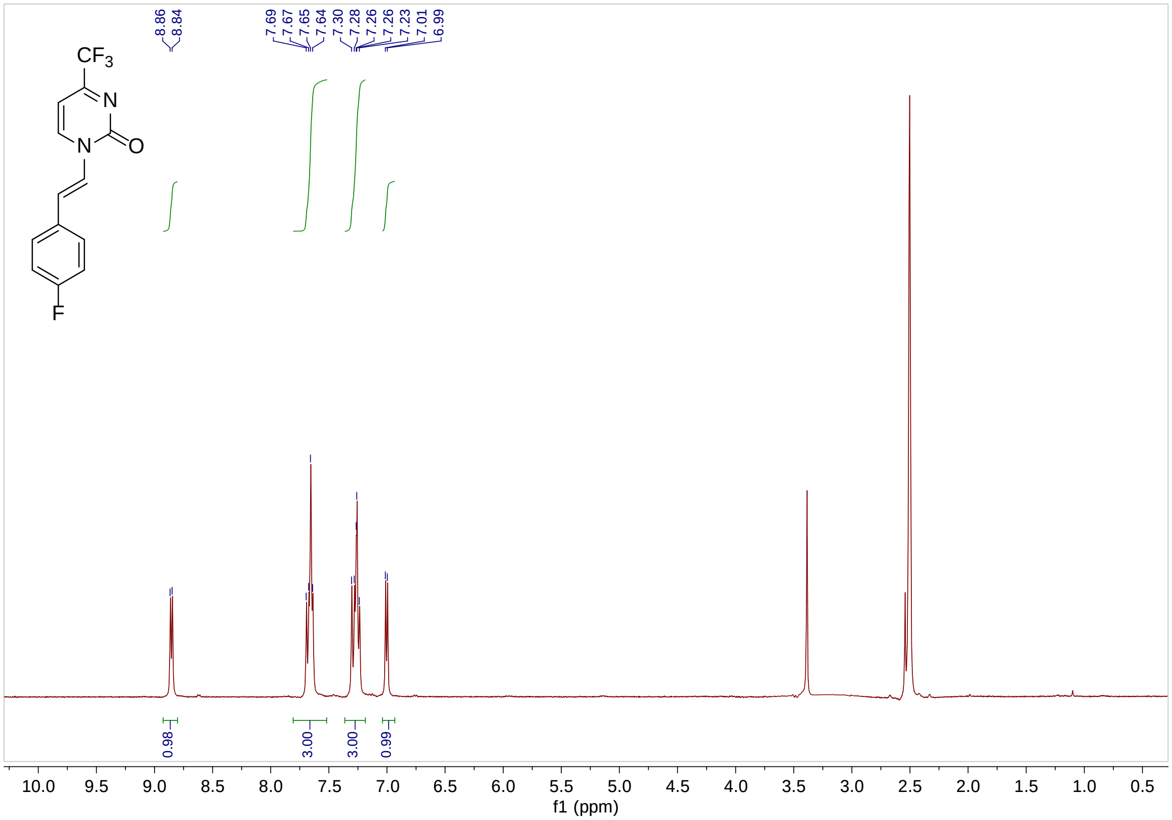

^{13}C NMR (125 MHz, DMSO-d_6):

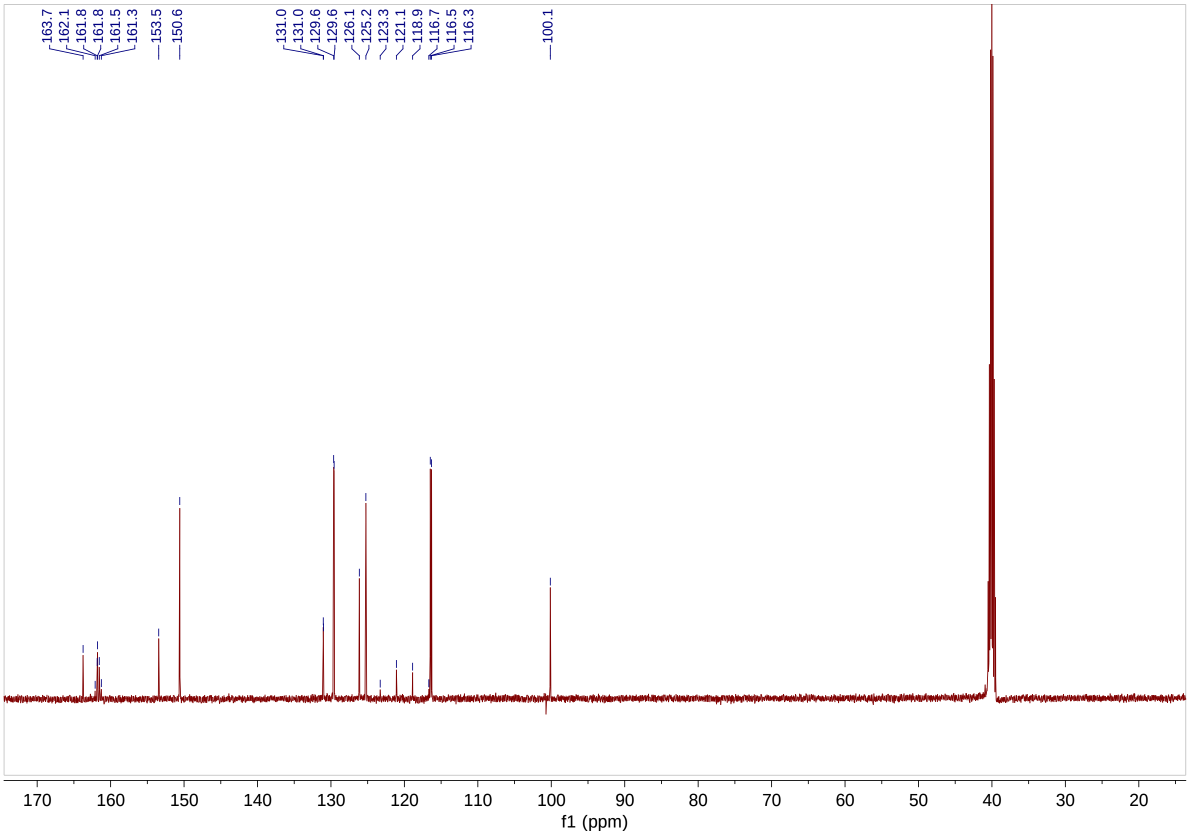

(*E*)-1-(4-Methoxystyryl)-4-(trifluoromethyl)pyrimidin-2(1*H*)-one (5e)

^{1}H NMR (400 MHz, DMSO-*d*$_6$):

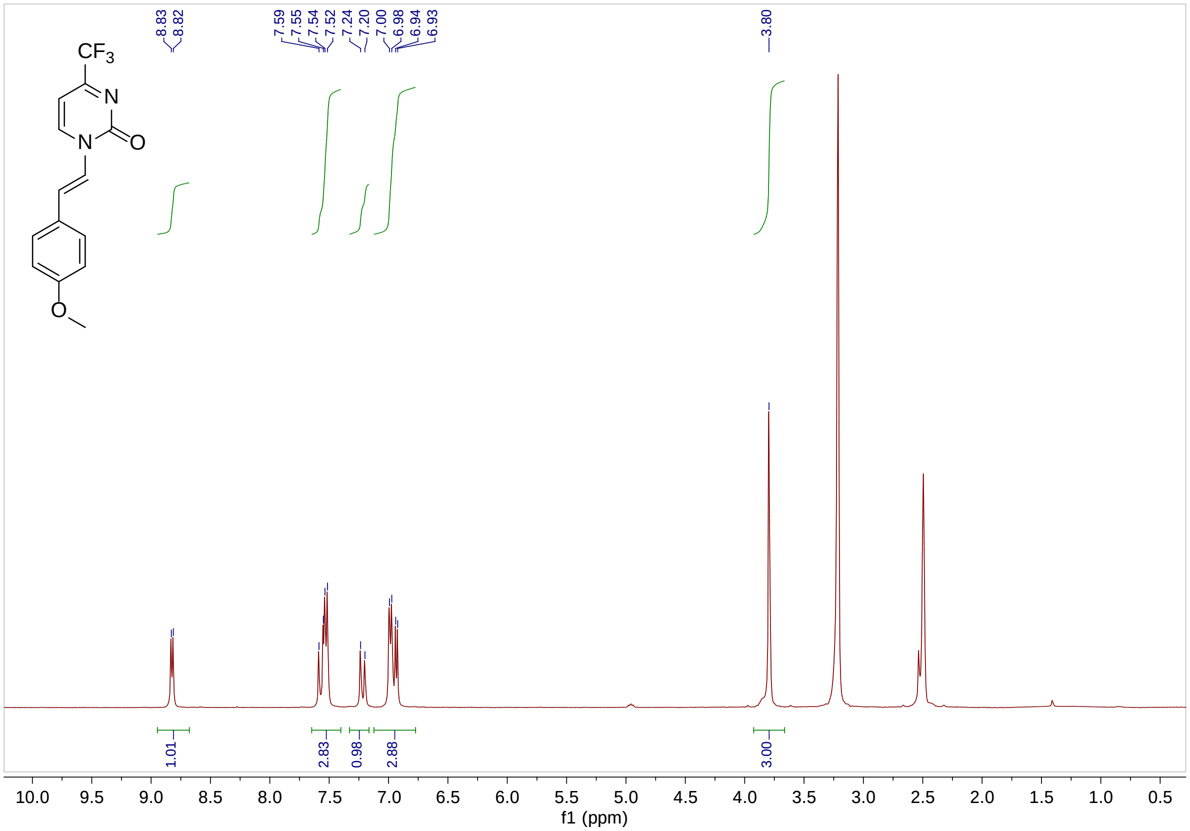

^{13}C NMR (125 MHz, DMSO-*d*$_6$):

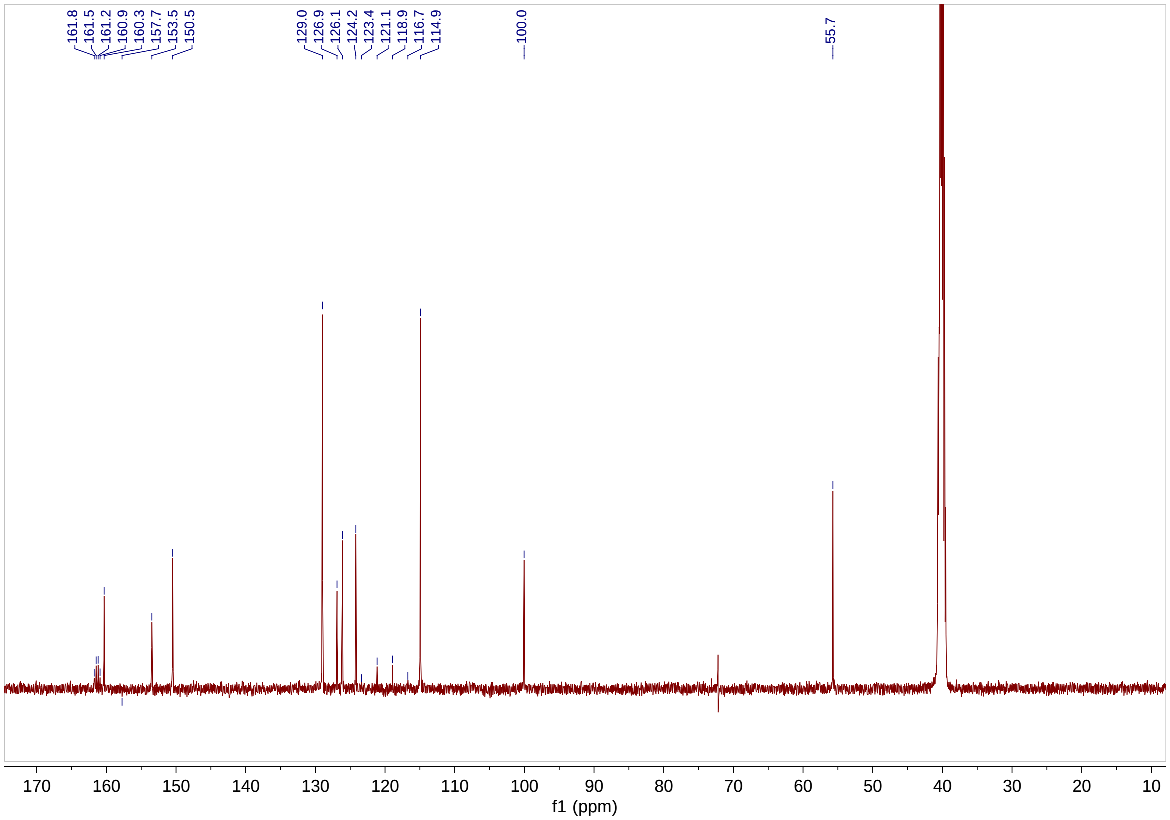

4-(Trifluoromethyl)-1-vinylpyrimidin-2(1*H*)-one (5f)

^{1}H NMR (400 MHz, DMSO-d_6):

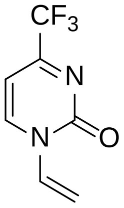

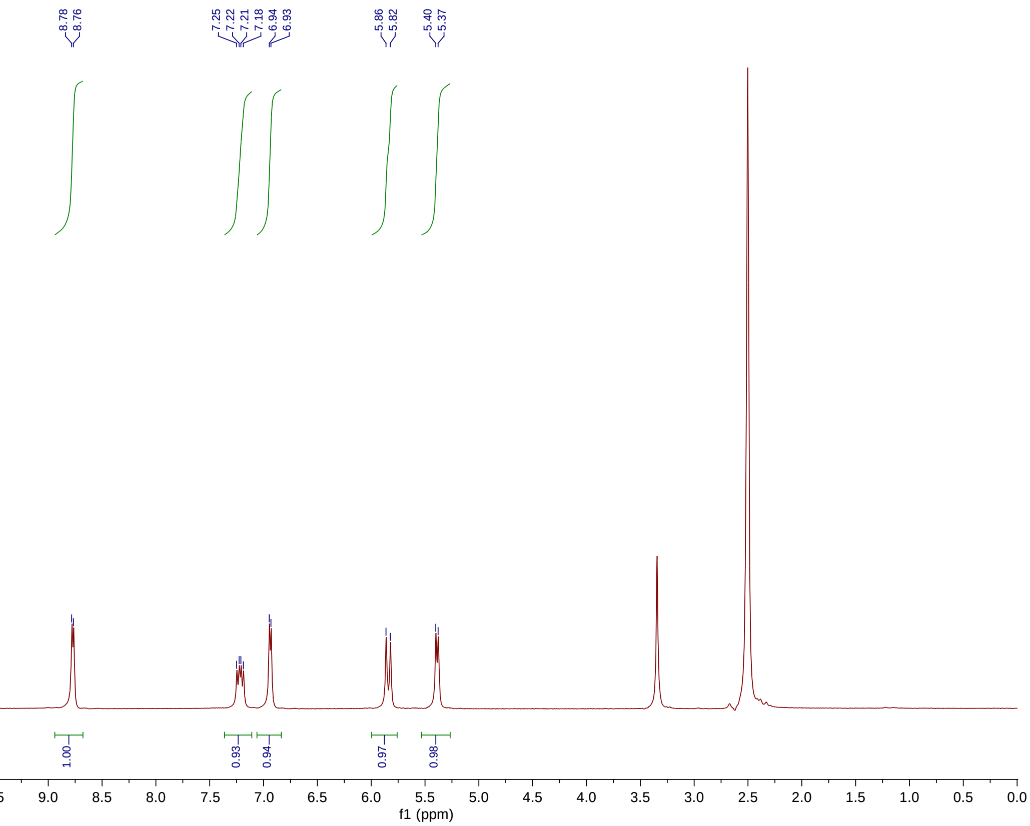

^{13}C NMR (125 MHz, DMSO-d_6):

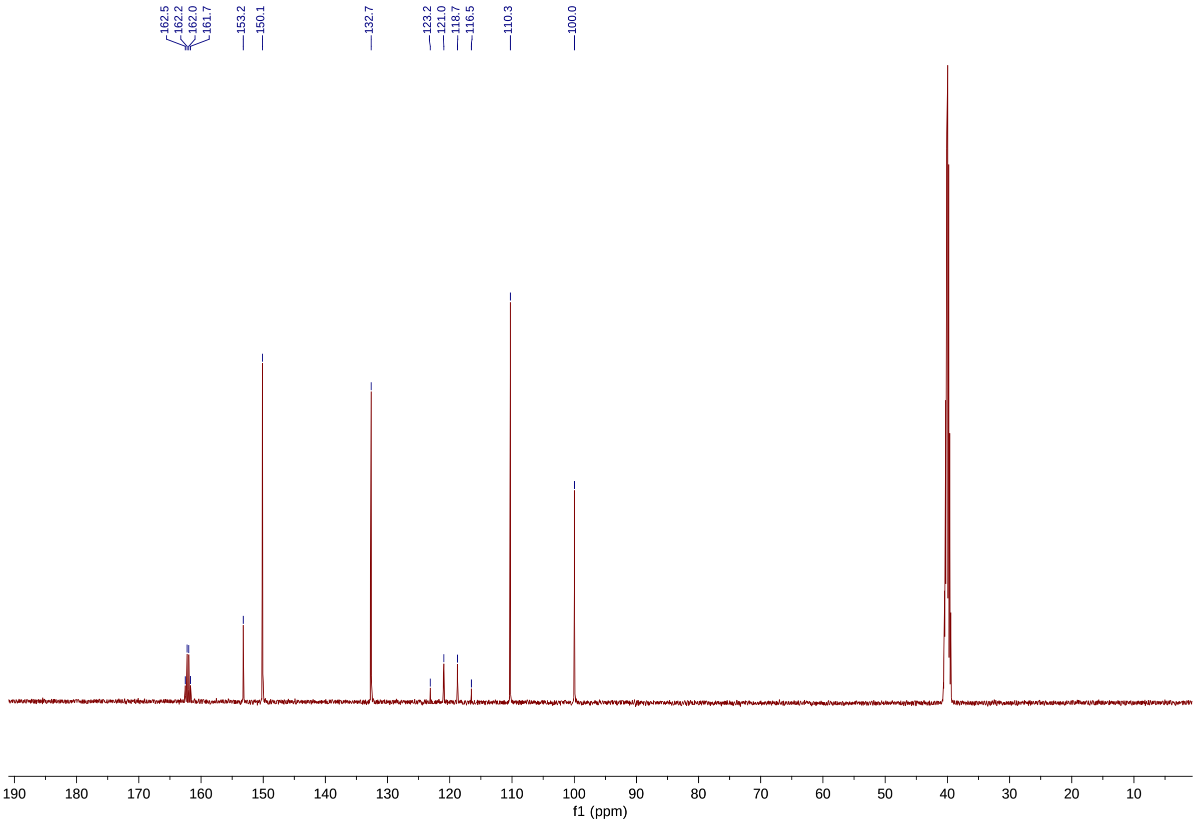

(E)-1-(Prop-1-en-1-yl)-4-(trifluoromethyl)pyrimidin-2(1*H*)-one (5g)

^{1}H NMR (400 MHz, DMSO-d_6):

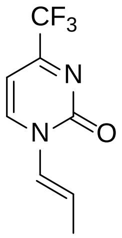

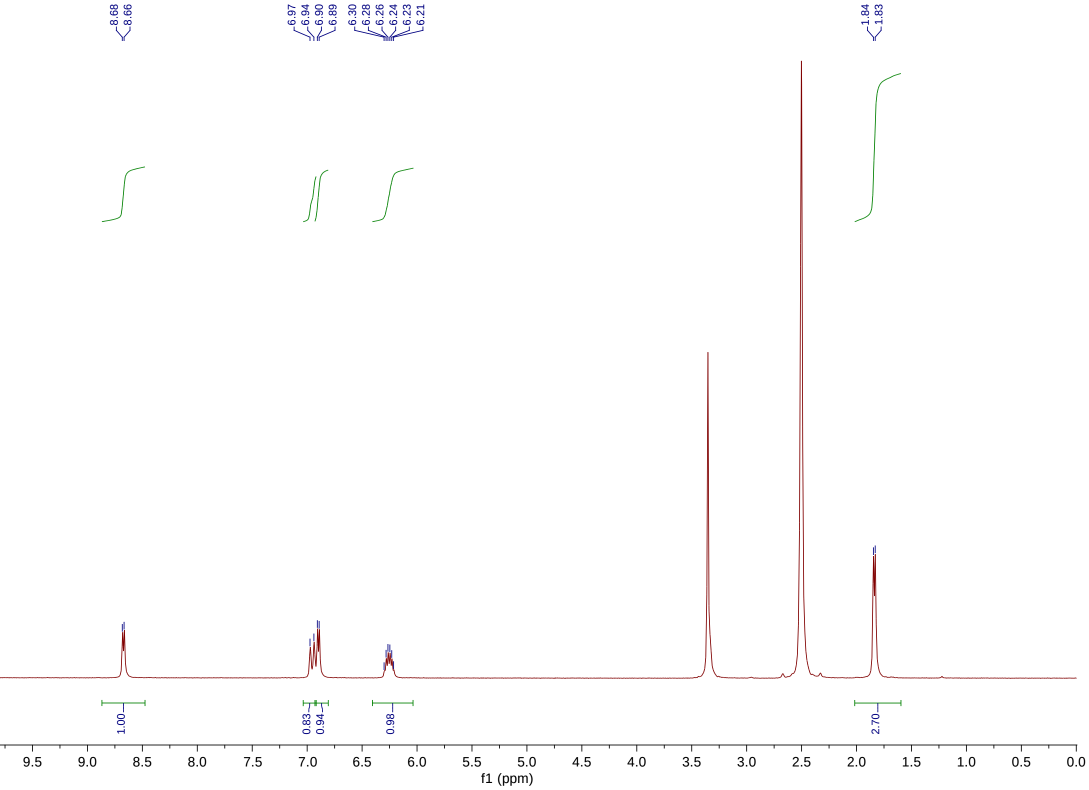

^{13}C NMR (150 MHz, DMSO-d_6):

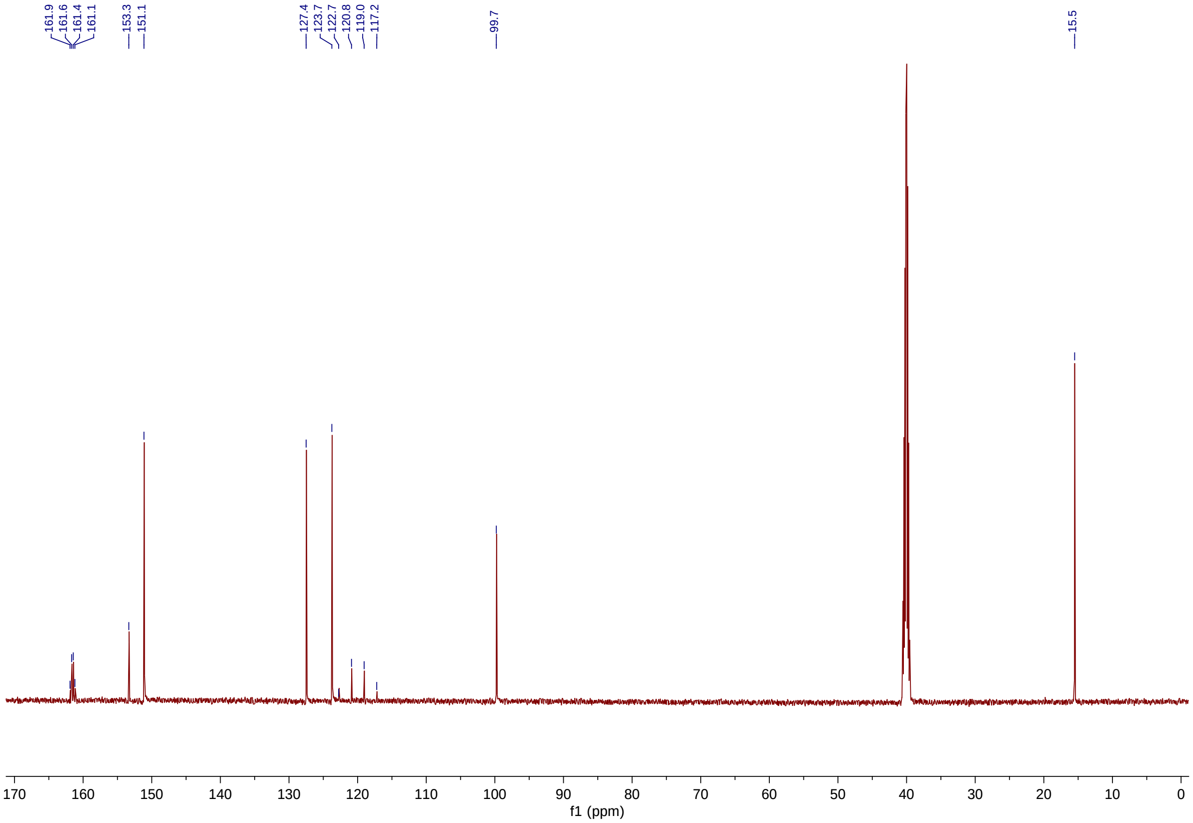

(E)-1-(2-Cyclopropylvinyl)-4-(trifluoromethyl)pyrimidin-2(1*H*)-one (5h)

^{1}H NMR (400 MHz, DMSO-d_6):

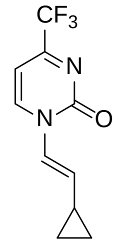

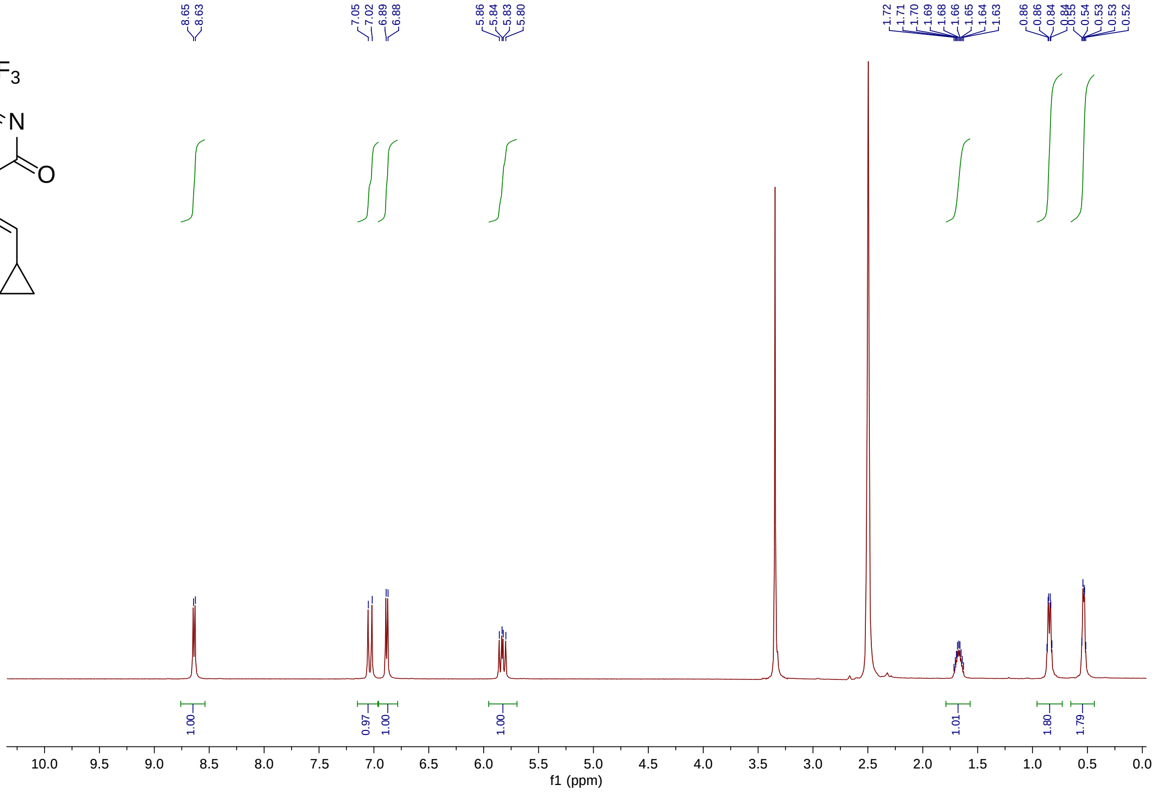

^{13}C NMR (150 MHz, DMSO-d_6):

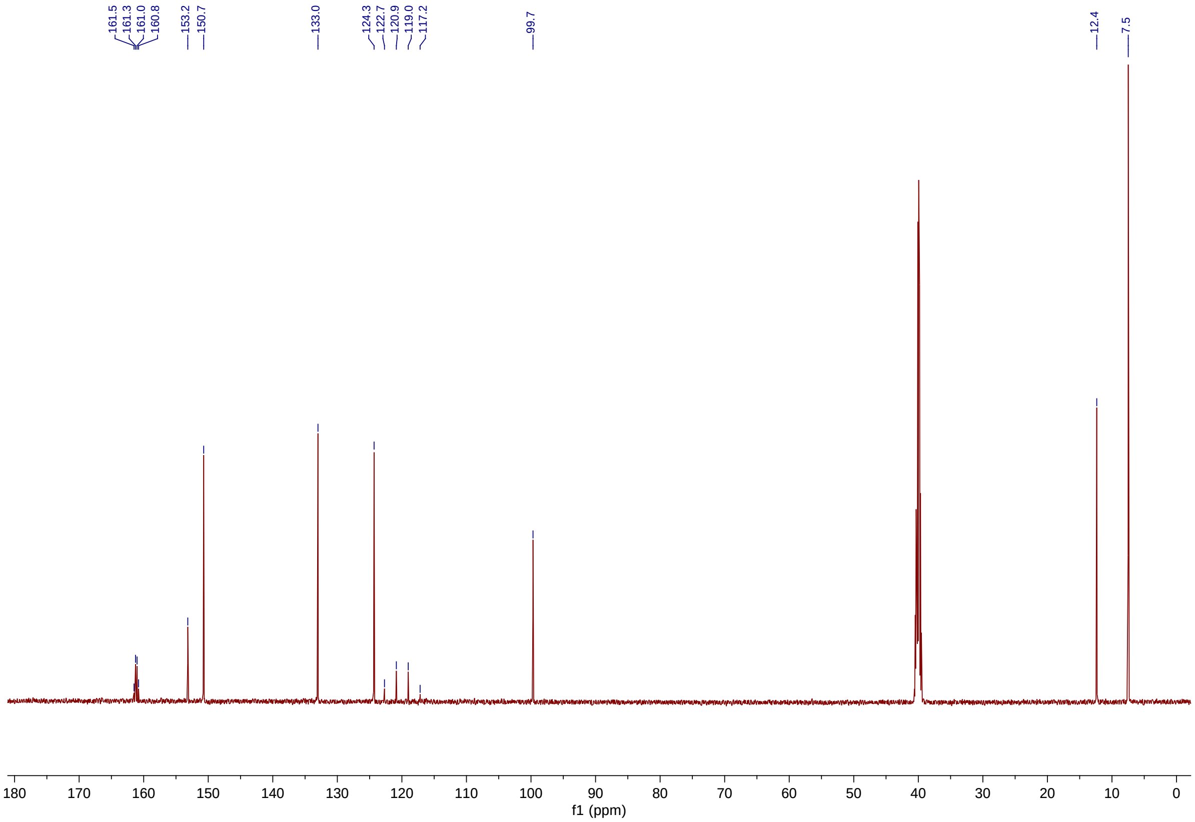

1,1'-(2,4-bis(3-Methoxyphenyl)cyclobutane-1,3-diyl)bis(4-(trifluoromethyl)pyrimidin-2(1*H*)-one) (8)

^{1}H NMR (400 MHz, DMSO-*d*$_6$):

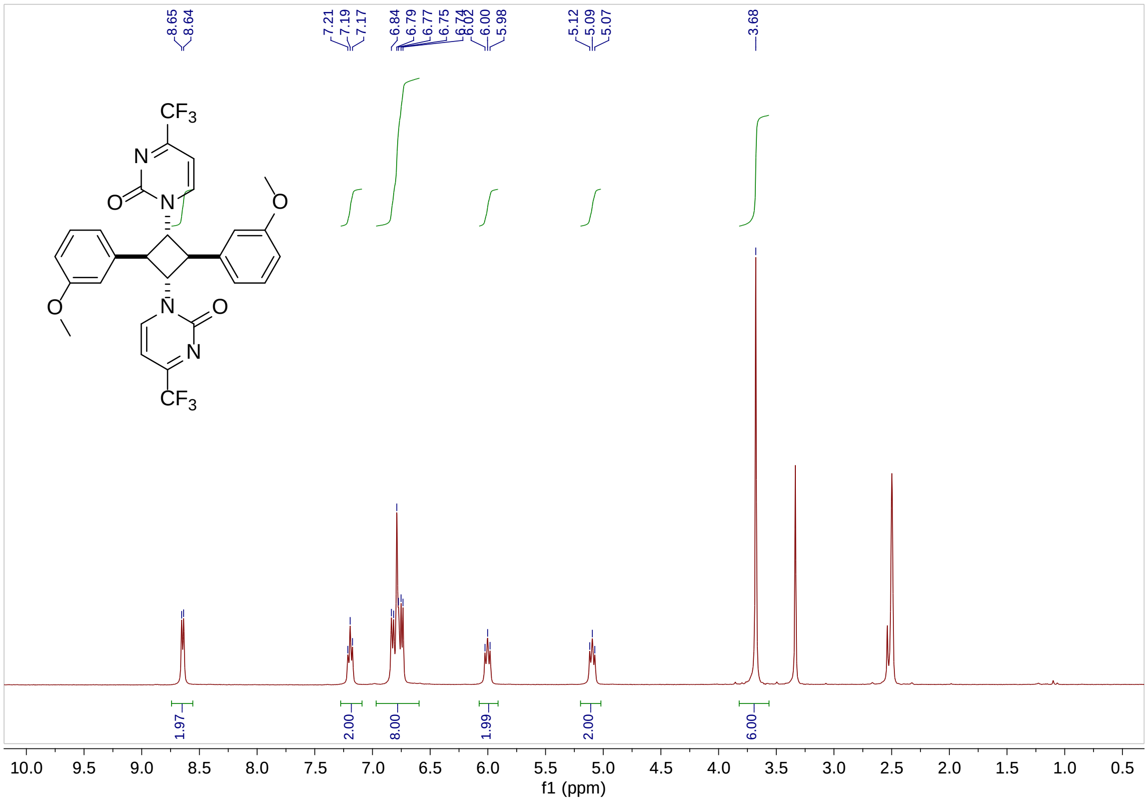

^{13}C NMR (125 MHz, DMSO-*d*$_6$):

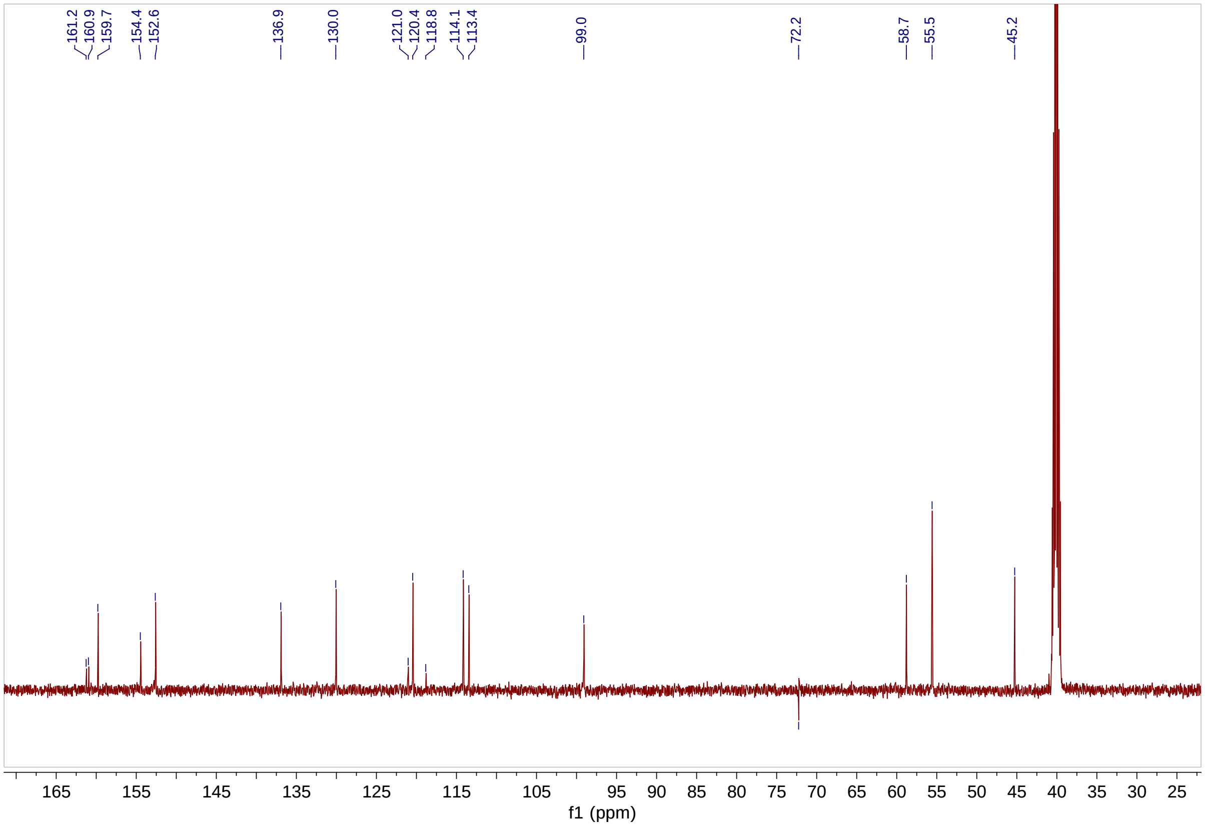

4-(Difluoromethyl)-1-phenylpyrimidin-2(1*H*)-one (9a)

^{1}H NMR (400 MHz, DMSO-d_6):

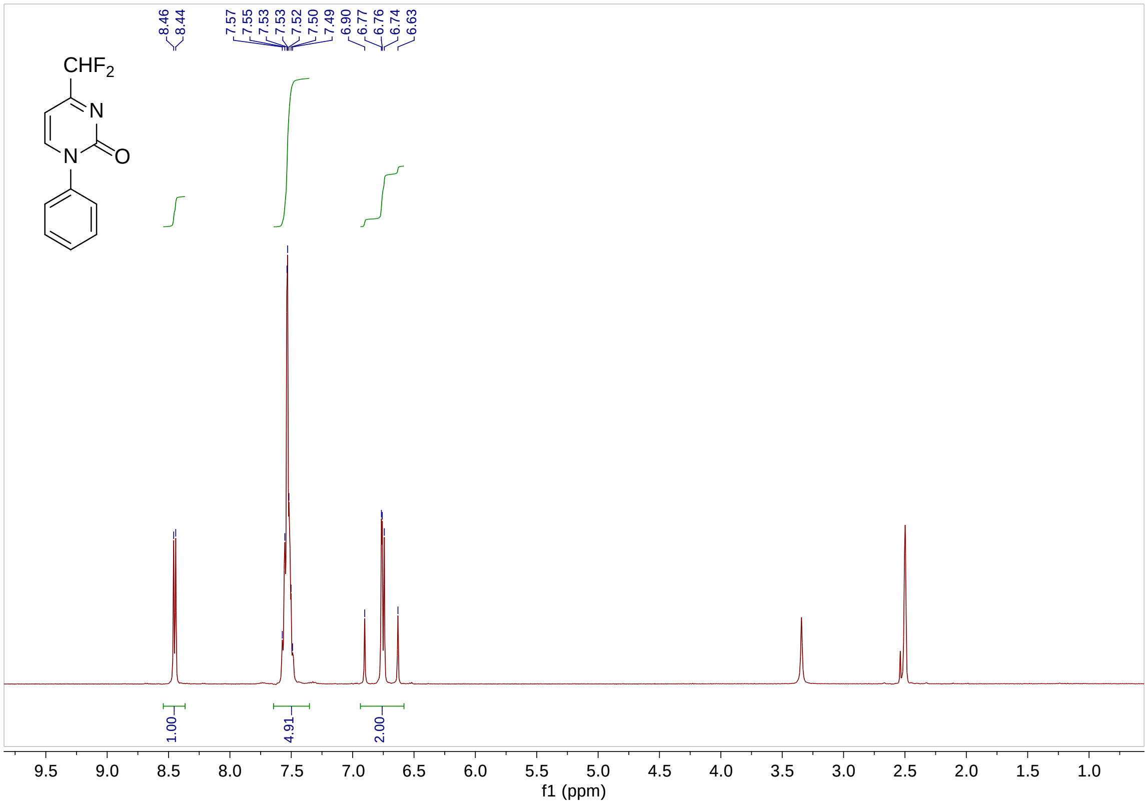

^{13}C NMR (125 MHz, DMSO-d_6):

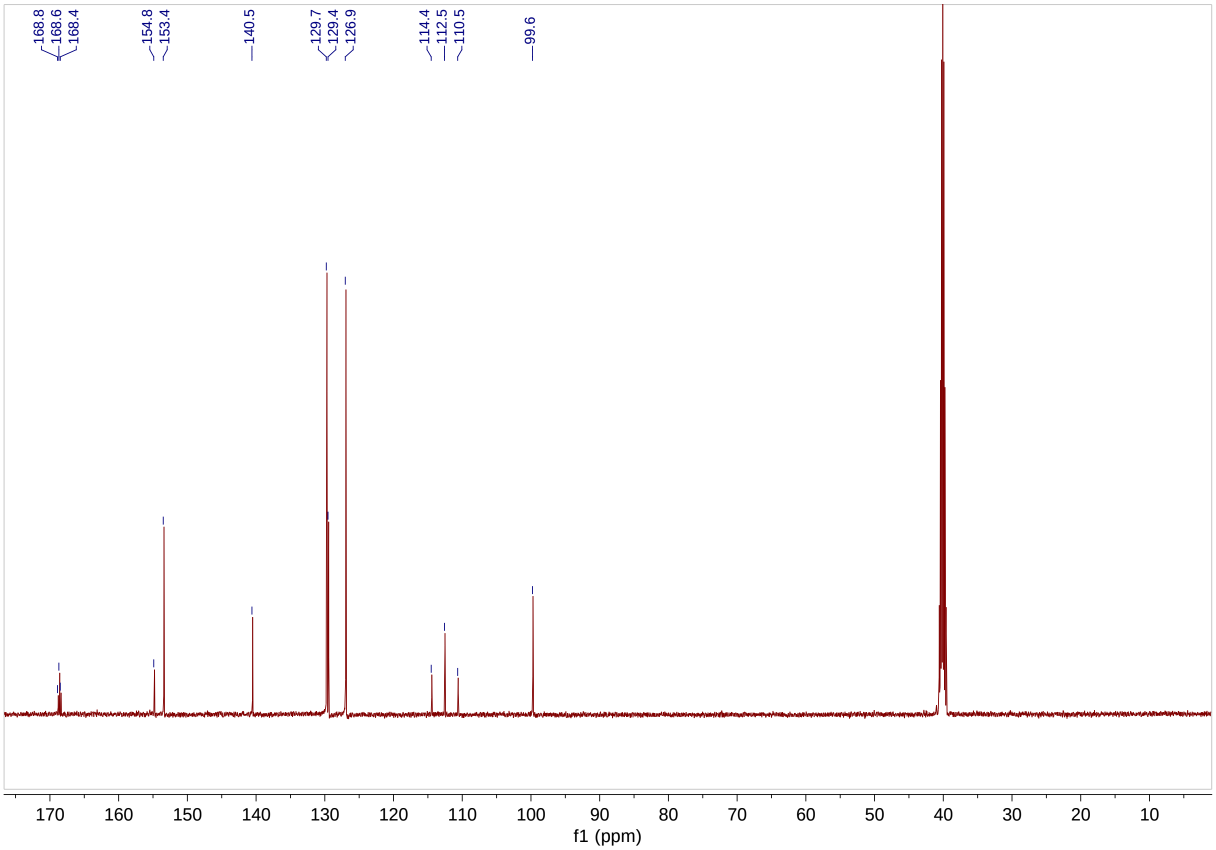

4-(Chlorodifluoromethyl)-1-phenylpyrimidin-2(1*H*)-one (9b)

^{1}H NMR (400 MHz, DMSO-d_6):

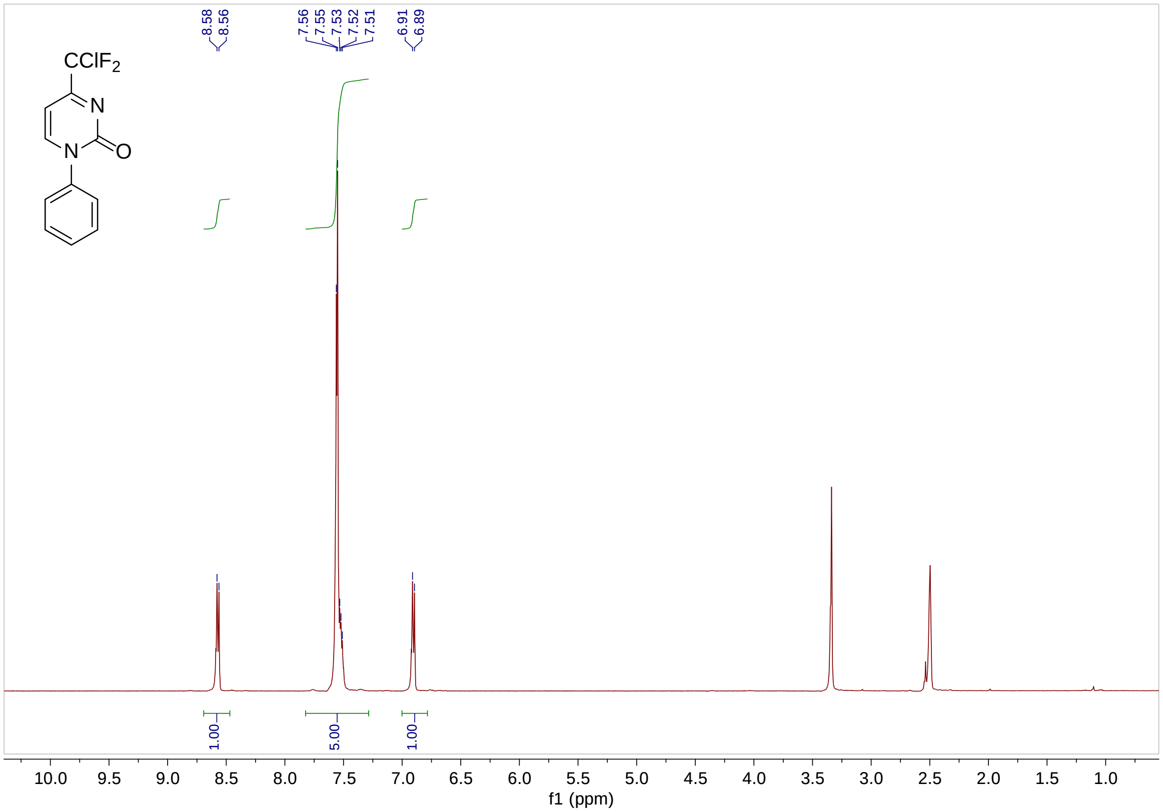

^{13}C NMR (125 MHz, DMSO-d_6):

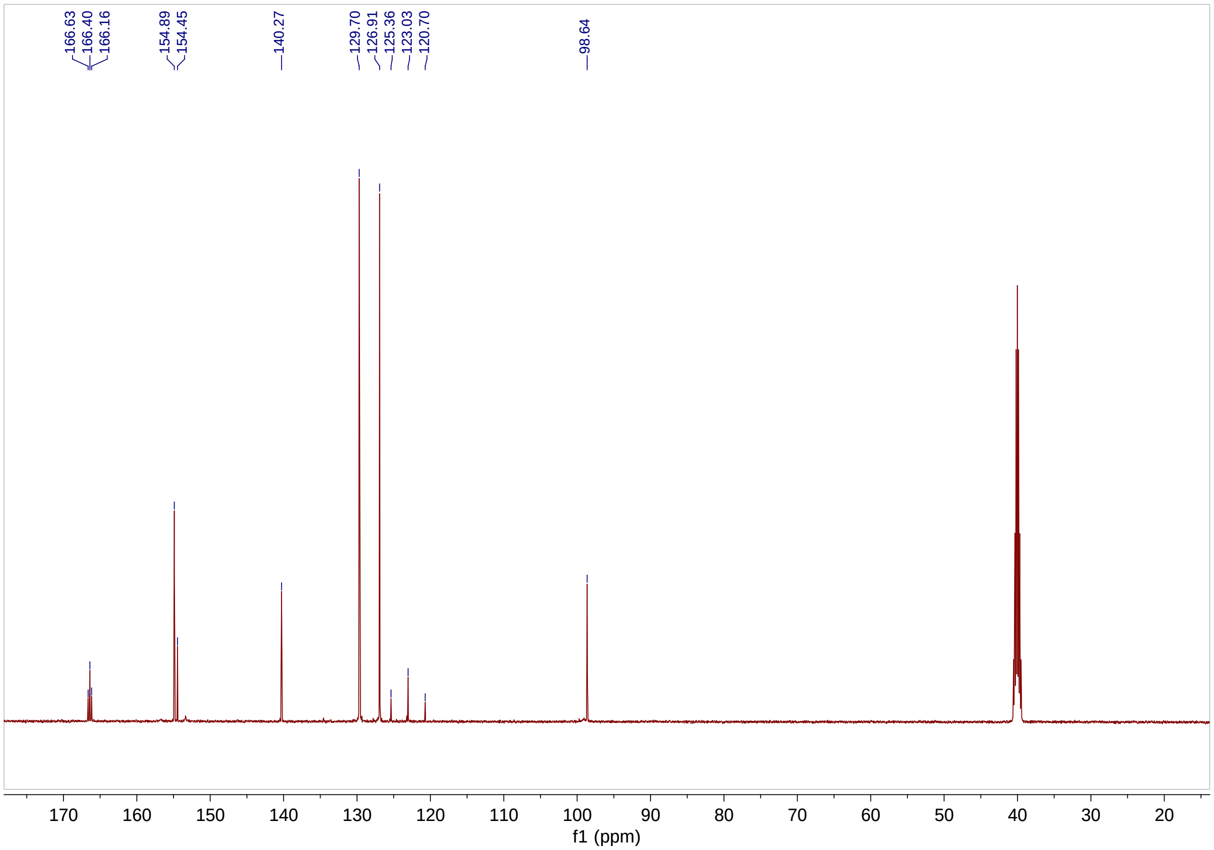

4-(Perfluoroethyl)-1-phenylpyrimidin-2(1*H*)-one (9c)

^{1}H NMR (400 MHz, DMSO-d_6):

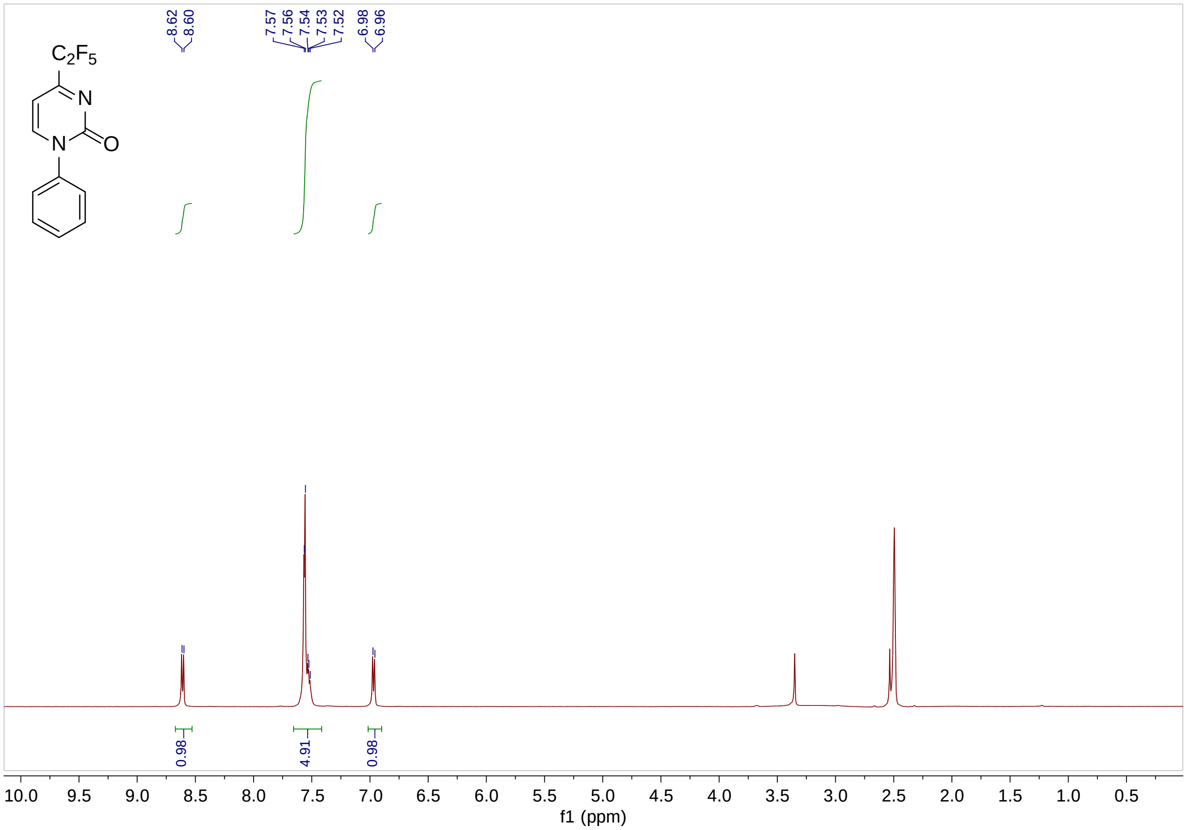

^{13}C NMR (125 MHz, DMSO-d_6):

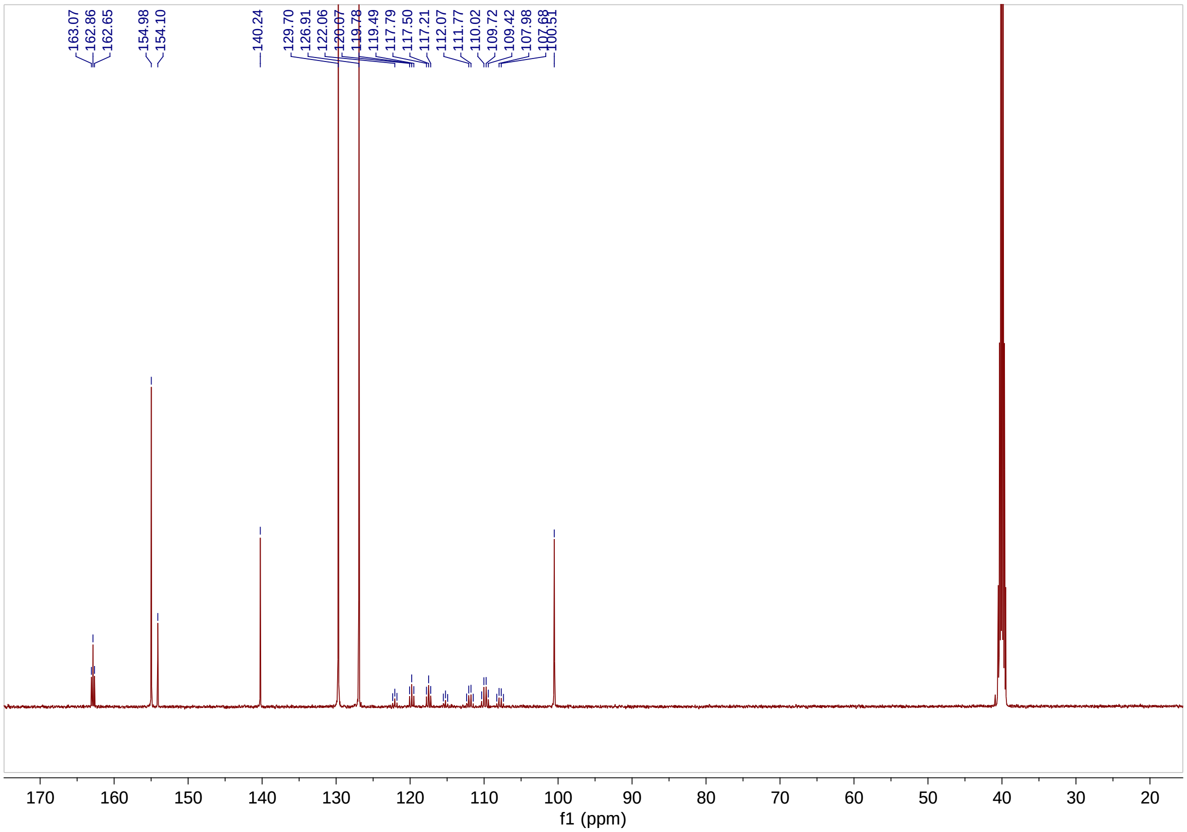

Ethyl 2-oxo-1-phenyl-4-(trifluoromethyl)-1,2-dihydropyrimidine-5-carboxylate (9f)

^{1}H NMR (400 MHz, DMSO-d_6):

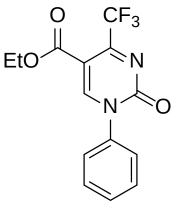

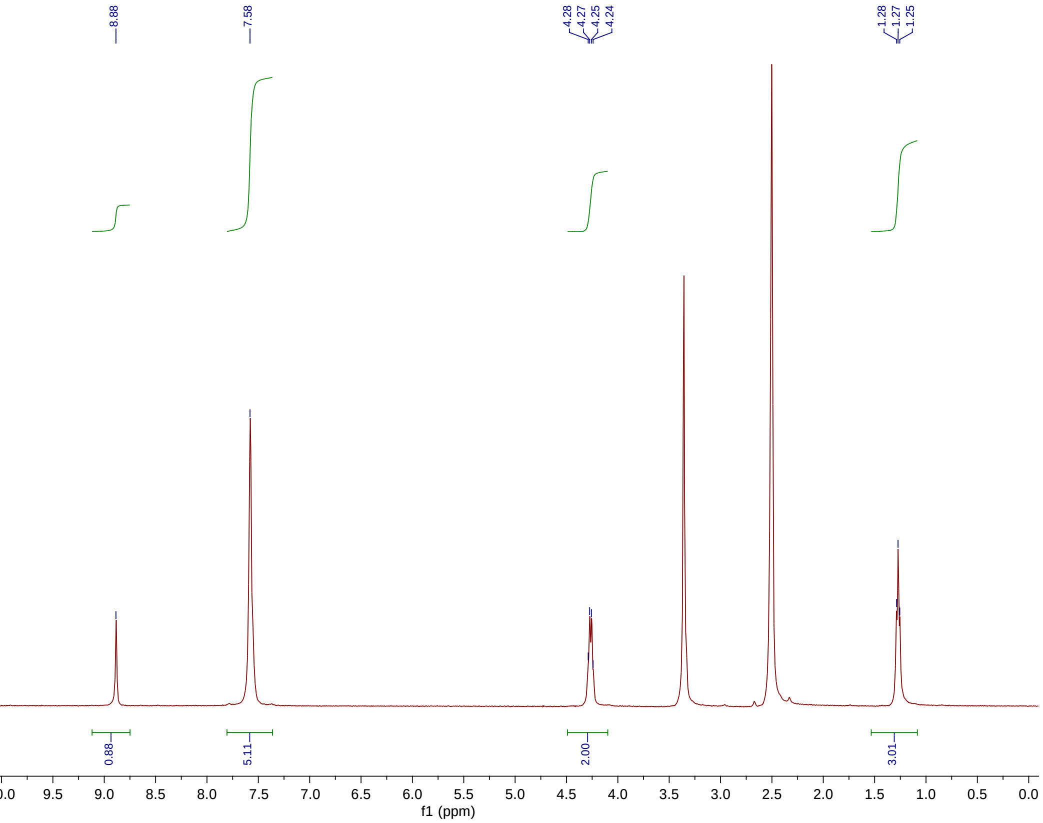

^{13}C NMR (125 MHz, DMSO-d_6):

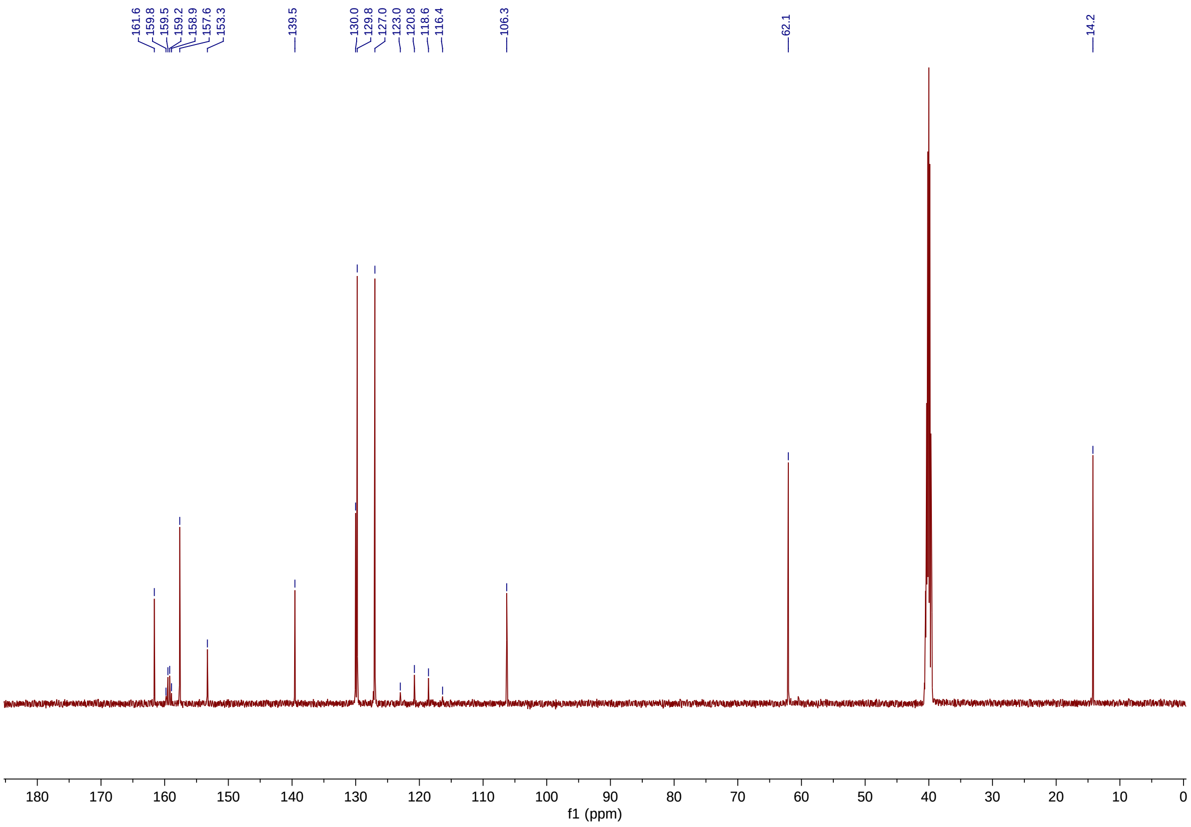

5-Bromo-1-phenyl-4-(trifluoromethyl)pyrimidin-2(1*H*)-one (9g)

^{1}H NMR (400 MHz, DMSO-d_6):

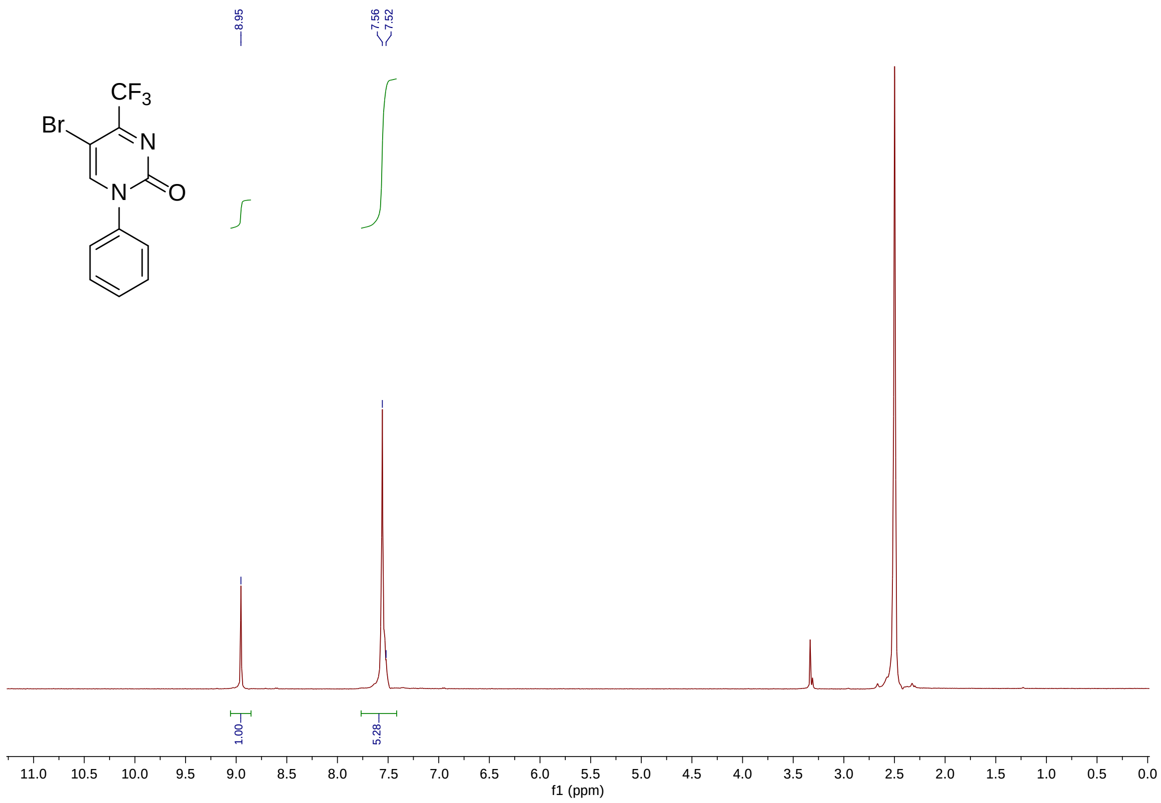

^{13}C NMR (125 MHz, DMSO-d_6):

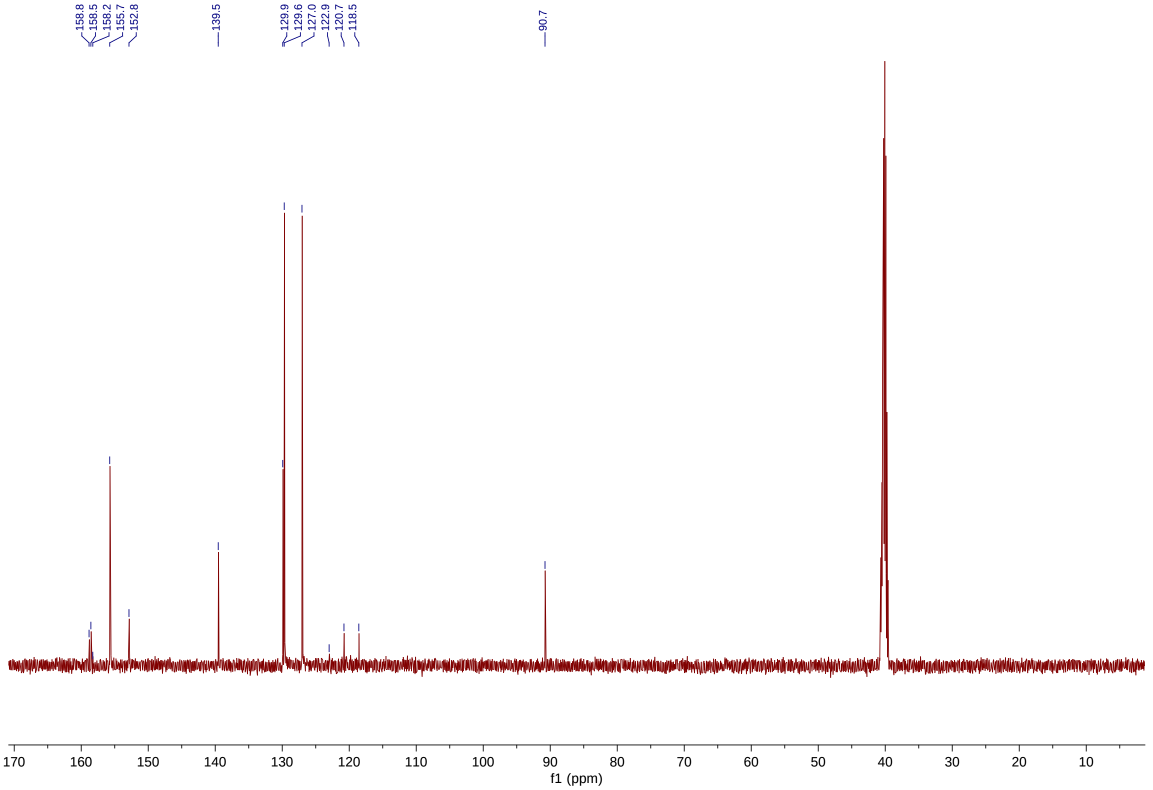

Supplement: File 1 — Experimental procedures, characterization data, copies of the 1H and 13C NMR spectra. [file Beilstein_J_Org_Chem-16-2304-s001.pdf]
